# Analysis of complete genome sequence and major surface antigens of *Neorickettsia helminthoeca*, causative agent of salmon poisoning disease

**DOI:** 10.1111/1751-7915.12731

**Published:** 2017-06-06

**Authors:** Mingqun Lin, Katherine Bachman, Zhihui Cheng, Sean C. Daugherty, Sushma Nagaraj, Naomi Sengamalay, Sandra Ott, Al Godinez, Luke J. Tallon, Lisa Sadzewicz, Claire Fraser, Julie C. Dunning Hotopp, Yasuko Rikihisa

**Affiliations:** ^1^ Department of Veterinary BiosciencesThe Ohio State University1925 Coffey RoadColumbusOH43210USA; ^2^ Institute for Genome SciencesUniversity of Maryland School of Medicine801 W. Baltimore StBaltimoreMD21201USA; ^3^ Department of MedicineUniversity of Maryland School of Medicine801 W. Baltimore StBaltimoreMD21201USA; ^4^ Department of Microbiology and ImmunologyUniversity of Maryland School of Medicine801 W. Baltimore StBaltimoreMD21201USA

## Abstract

*Neorickettsia helminthoeca*, a type species of the genus *Neorickettsia*, is an endosymbiont of digenetic trematodes of veterinary importance. Upon ingestion of salmonid fish parasitized with infected trematodes, canids develop salmon poisoning disease (SPD), an acute febrile illness that is particularly severe and often fatal in dogs without adequate treatment. We determined and analysed the complete genome sequence of *N. helminthoeca*: a single small circular chromosome of 884 232 bp encoding 774 potential proteins. *N. helminthoeca* is unable to synthesize lipopolysaccharides and most amino acids, but is capable of synthesizing vitamins, cofactors, nucleotides and bacterioferritin. *N. helminthoeca* is, however, distinct from majority of the family *Anaplasmataceae* to which it belongs, as it encodes nearly all enzymes required for peptidoglycan biosynthesis, suggesting its structural hardiness and inflammatory potential. Using sera from dogs that were experimentally infected by feeding with parasitized fish or naturally infected in southern California, Western blot analysis revealed that among five predicted *N. helminthoeca* outer membrane proteins, P51 and strain‐variable surface antigen were uniformly recognized. Our finding will help understanding pathogenesis, prevalence of *N. helminthoeca* infection among trematodes, canids and potentially other animals in nature to develop effective SPD diagnostic and preventive measures. Recent progresses in large‐scale genome sequencing have been uncovering broad distribution of *Neorickettsia* spp., the comparative genomics will facilitate understanding of biology and the natural history of these elusive environmental bacteria.

## Introduction

Salmon poisoning disease (SPD), an acute and often‐fatal illness in wild and domestic canids, was first discovered in the 1800s when early settlers in Pacific Northwest noted their dogs becoming ill following ingestion of salmon (Philip, 1955). In 1950, a bacterial pathogen was implicated as the causative agent of SPD and named *Neorickettsia helminthoeca*, due to its biological similarity to the members of the family Rickettsiaceae and the novel invertebrate/helminth vector (Cordy and Gorham, 1950; Philip, 1955). *N. helminthoeca* exists in all life stages of the fluke *Nanophyetus salmincola* (Bennington and Pratt, 1960; Schlegel *et al*., 1968), which has a complicated digenetic life cycle involving both pleurocid freshwater snails (*Oxytrema silicula*) and salmonid fish as intermediate hosts (Millemann and Knapp, 1970; Headley *et al*., 2011). Due to the limited geographic range of the vector and intermediate hosts, the distribution of SPD was thought to be limited to the northern Pacific coast. However, SPD cases have been confirmed in southern California (this study; Veterinary Practice News, 2009), Vancouver Island, Canada (Booth *et al*., 1984), and Maringa, Brazil, using immunohistochemical, histopathological and molecular diagnostic techniques (Table 1; Headley *et al*., 2004, 2006, 2011), although the vector and life cycle in these regions remain to be identified. The expansion of the geographic distribution of SPD where *N. salmincola* has not been documented suggests the potential adaptation of this organism to other trematode vectors.

**Table 1:** Biological characteristics of *Neorickettsia* species

Species	Vertebrate host	Invertebrate vector/host^a^	*In vivo*‐infected mammalian cells	Diseases & symptoms	Geographic distribution
*N. helminthoeca*	Canidae	Digenetic trematode *Nanophyetus salmincola* in snails (*Oxytrema silicula*) and fish (salmonid)	Monocytes and Macrophages	Salmon Poisoning Disease (pyrexia, anorexia, ocular discharge, weight loss, lethargy and dehydration, > 90% mortality)	California, Washington, Oregon, Idaho, Canada, Brazil
*N. risticii*	Horse, Bat	Digenetic trematode *Acanthatrium oregonense* in snails (*Elimia virginica*) and aquatic insects (caddisflies, mayflies)	Monocytes, Macrophages, intestinal epithelial cells and mast cells	Potomac horse fever (fever, depression, anorexia, dehydration, watery diarrhoea, laminitis and/or abortion, ~9% fatality)	USA, Canada, Brazil, Uruguay
*N. sennetsu*	Human	Unknown trematodes in snails and grey mullet fish	Monocytes and Macrophages	Sennetsu neorickettsiosis (fever, fatigue, general malaise and lymphadenopathy)	Japan, South‐East Asia

**a.** Transmission mode: all *Neorickettsia* spp. are transstadially and vertically transmitted through generations of trematodes.

While there is a large range of definitive hosts for the trematode, *N. helminthoeca* causes severe SPD in members of the Canidae family including dogs, foxes and coyotes (Cordy and Gorham, 1950; Philip *et al*., 1954a,b; Philip, 1955; Foreyt *et al*., 1987). Dogs most commonly acquire SPD when they eat raw or undercooked salmonid fish containing encysted trematodes infected with *N. helminthoeca*. Upon ingestion, the metacercariae stage of the trematode matures in the intestinal lumen for 5–8 days and releases the bacteria to be picked up by monocytes and macrophages in the intestinal wall. The exact mechanism of bacterial entry into these cells is not known, but morphological studies demonstrate the organism existing as clusters termed morulae or singly within a host cell‐derived membrane vacuole in the cytoplasm of the canine host cell (Rikihisa *et al*., 1991). *N. helminthoeca‐*infected cells travel throughout the circulation and accumulate in the thoracic and abdominal lymph nodes with the mesenteric and ileocecal lymph nodes being most commonly affected (Philip *et al*., 1954a; Philip, 1955; Headley *et al*., 2011). Symptoms begin with pyrexia (39.8–40.9°C) that persists for 6–7 days and anorexia (Rikihisa *et al*., 1991). Dogs progress to vomiting and diarrhoea that may or may not contain blood 4–6 days following development of a fever. Other symptoms include ocular discharge, weight loss, lethargy and dehydration. If left untreated, death occurs 2–10 days after development of symptoms (Philip, 1955). Current therapies for SPD include fluid therapy, blood transfusions for haemorrhagic diarrhoea, anti‐helminthic praziquantel and oral doxycycline or intravenous oxytetracycline. Affected individuals produce specific immunity to SPD following recovery from the disease (Philip *et al*., 1954a; Philip, 1955).

**Figure 1:**
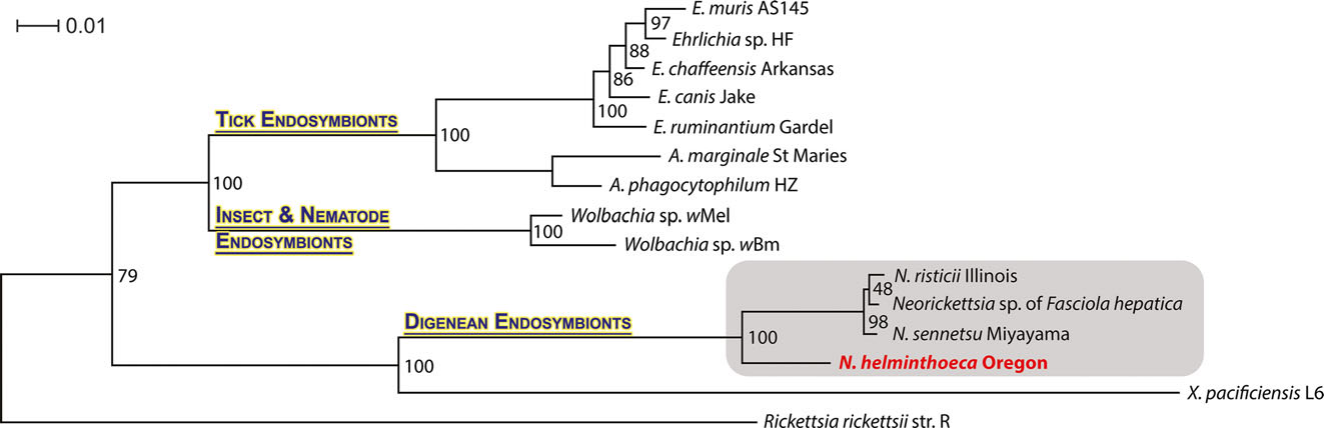
Phylogenetic tree of the family Anaplasmataceae. 16S rRNA sequences of members of the family Anaplasmataceae were aligned using ClustalW, a phylogenetic tree was built using raxml, and the tree was visualized with Dendroscope as described in the ‘Experimental procedures’. Grey box highlights *Neorickettsia* species. GenBank Accession numbers and locus tag numbers for the 16S rRNA sequences are *N. helminthoeca* Oregon, NZ_CP007481/NHE_RS00195; *N. risticii* Illinois, NC_013009.1/NRI_RS00185; *N. sennetsu* Miyayama, NC_007798.1/NSE_RS00200; *A. phagocytophilum* HZ, NC_007797.1/APH_RS03965; *A. marginale* Florida, NC_012026.1/AMF_RS06130; *E. chaffeensis* Arkansas, NC_007799.1/ECH_RS03785; *E. canis* Jake, NC_007354.1/ECAJ_RS00995; *E. ruminantium* Welgevonden, NC_005295.2/ERUM_RS01035; *E. muris* AS145, NC_023063.1/MR76_RS00900; *Ehrlichia* sp. HF, NZ_CP007474.1/EHF_RS03625; *Wolbachia pipientis* wMel, NC_002978.6/WD_RS05540; *Wolbachia* endosymbiont of *Brugia malayi*, NC_006833.1/WBM_RS02885; *Rickettsia rickettsii* str. R, L36217; *Neorickettsia* Endobacterium of *Fasciola hepatica*, LNGI01000001/AS219_00180; *Candidatus* ‘Xenolissoclinum pacificiensis L6’, AXCJ01000001/P857_926.

*Neorickettsia* species are obligatory intracellular α‐proteobacteria that belong to the family Anaplasmataceae in the order Rickettsiales (Rikihisa *et al*., 2005). *Neorickettsia* spp. are the deepest branching lineage in the family Anaplasmataceae, whereas *Anaplasma* and *Ehrlichia* are sister genera that share a common ancestor with *Wolbachia* spp. (Fig. 1; Pretzman *et al*., 1995; Wen *et al*., 1995, 1996). The branching pattern suggests that the speciation of *N. helminthoeca* occurred earlier than the speciation of *N. risticii* and *N. sennetsu*. These findings and many other molecular phylogenetic analyses (Anderson *et al*., 1992; Wen *et al*., 1995, 1996; Rikihisa *et al*., 1997) led to the drastic reclassification of the family Anaplasmataceae (Dumler *et al*., 2001).

Currently, only three pathogenic species of *Neorickettsia*, namely *N. helminthoeca* (type species), *N. sennetsu* (agent of human Sennetsu fever) and *N. risticii* (agent of Potomac horse fever) have been culture isolated and characterized in sufficient details with documented biological and medical significance (Table 1; Rikihisa *et al*., 1991, 2005). All of them are known to transmit from trematodes to monocytes/macrophages of mammals (dogs, humans and horses, respectively) and cause severe, sometimes fatal illnesses (Table 1; Rikihisa *et al*., 2005). In addition, the *Stellantochasmus falcatus* (SF) agent, which is closely related to *N. risticii*, was culture isolated from *S. falcatus* fluke encysting the grey mullet fish in Japan (Wen *et al*., 1996) and from fish in Oregon (Rikihisa *et al*., 2004). The initial 16S rRNA gene sequence‐based phylogenetic analysis of *N. helminthoeca* revealed that the divergence of 16S rRNA sequences is around 5% between *N. helminthoeca* and *N. risticii* or *N. sennetsu,* whereas it is only 0.7% between *N. risticii* and *N. sennetsu*.

As endosymbionts of digenetic trematodes (parasitic flatworms or flukes), *Neorickettsia* species are abundant in nature and have been identified throughout the life cycle of the trematodes and the hosts of trematodes including the essential first intermediate host of snails, the second intermediate hosts such as fish and aquatic insects and the definitive hosts such as mammals and birds wherein the trematodes sexually reproduce fertilized eggs (Cordy and Gorham, 1950; Philip *et al*., 1954a,b; Philip, 1955; Foreyt *et al*., 1987; Gibson *et al*., 2005; Rikihisa *et al*., 2005; Gibson and Rikihisa, 2008; Greiman *et al*., 2016). Recent reports revealed more than 10 new genotypes of *Neorickettsia* in divergent digenean families throughout the world, including Asia, Africa, Australia, Americas and even Antarctica (Ward *et al*., 2009; Tkach *et al*., 2012; Greiman *et al*., 2014, 2017), suggesting a global distribution of *Neorickettsia* spp. Notably, a *Neorickettsia* sp. was found in the medically important trematode *Fasciola hepatica* (the liver fluke, fasciolosis disease agent) isolated from a sheep in Oregon US (McNulty *et al*., 2017). In addition, a related new species named *Candidatus* ‘Xenolissoclinum pacificiensis L6’ was identified in the ascidian tunicate *Lissoclinum patella*, a marine chordate animal at the coast of Papua New Guinea (Kwan and Schmidt, 2013), implicating even boarder distribution of *Neorickettsia‐*like bacteria among diverse invertebrates. To date, the complete genome sequences have been determined only for *N. sennetsu* (Dunning Hotopp *et al*., 2006) and *N. risticii* (Lin *et al*., 2009), and almost complete genome sequences were obtained for *Neorickettsia* endobacterium of *F. hepatica* (*NFh*) and *Candidatus* ‘X. pacificiensis’ (Kwan and Schmidt, 2013; McNulty *et al*., 2017). The phylogenetic analysis based on 16S rRNA gene sequences suggests that *NFh* shares > 99% identity with *N. risticii* and *N. sennetsu*, while *Candidatus* ‘X. pacificiensis’ is distantly related to *Neorickettsia* spp. (Fig. 1). Genomic comparisons indicated that approximately 97% of the predicted proteins (721 of 744) of *NFh* showed top matches to *N. risticii* or *N. sennetsu*, while 22 unique proteins of *NFh* were hypothetical proteins without functional annotations (McNulty *et al*., 2017).

Because the mortality rate of SPD is > 90% without rapid antibiotic treatment (Philip, 1955; Rikihisa *et al*., 1991), the current inefficient diagnostic method (faecal examination for parasite eggs and/or Romanowsky staining of lymph node aspirates), and the expansion of the geographic distribution of SPD, there remains a need for better understanding of *N. helminthoeca* and development of a simple and rapid serodiagnostic approach. In this study, we sought to (i) determine the complete genome of *N. helminthoeca* and compare with closely related *N. risticii* and *N. sennetsu* genomes, (ii) determine, clone and purify putative immunodominant major outer membrane proteins (OMPs), and (iii) test immunoreactivity of these recombinant OMPs using sera from dogs that were experimentally or naturally infected with *N. helminthoeca*.

## Results and discussion

### General features of the genome

**Figure 2:**
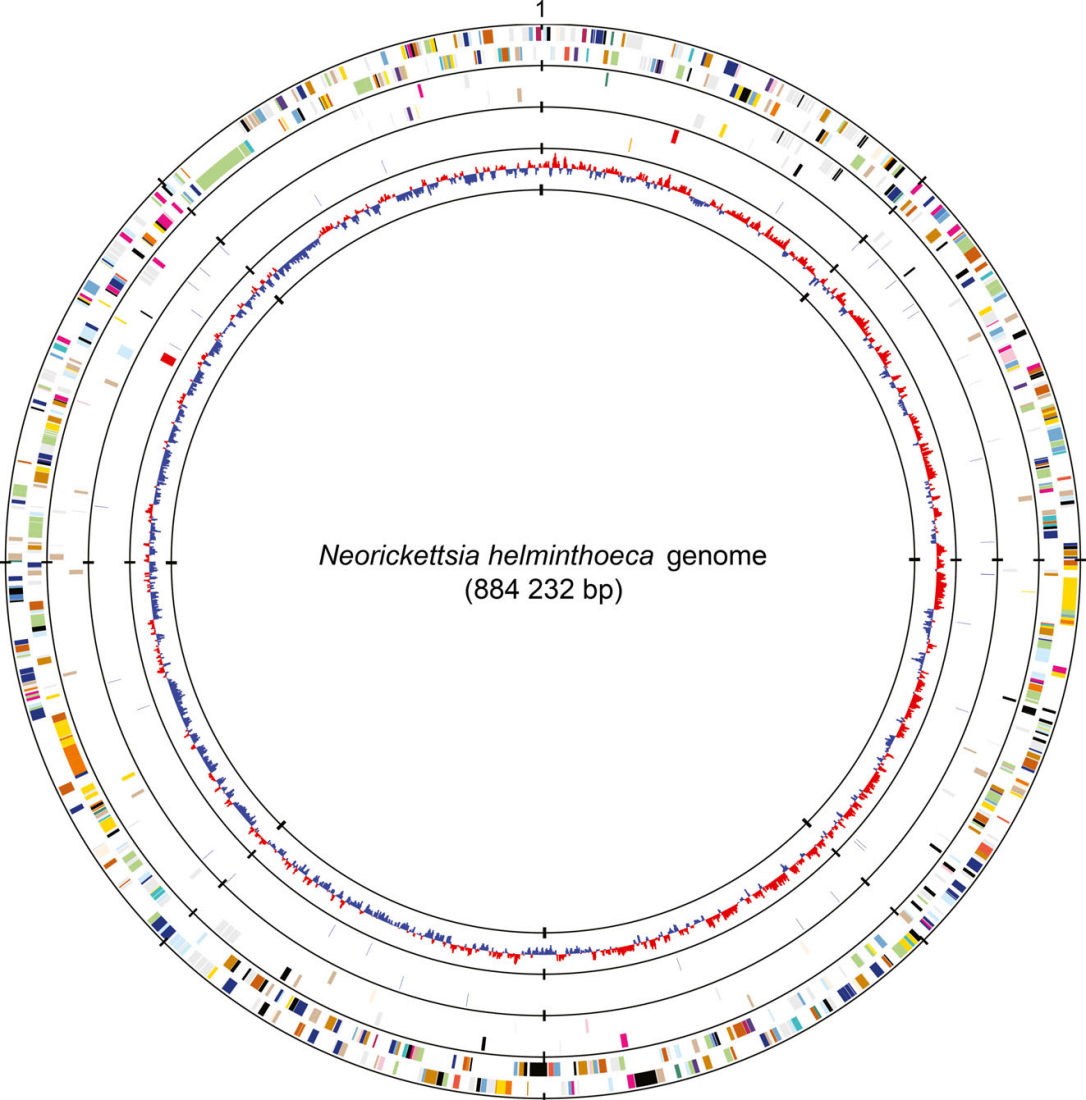
Circular representation of the genome of *N. helminthoeca*. From outside to inside, the first circle represents predicted protein‐coding sequences (ORFs) on the plus and minus strands respectively. The second circle represents the unique ORFs of *N. helminthoeca* in the three‐way comparison with *N. risticii* and *N. sennetsu*. Colours indicate the functional role categories of ORFs – dark grey: hypothetical proteins or proteins with unknown functions; gold: amino acid and protein biosynthesis; sky blue: purines, pyrimidines, nucleosides and nucleotides; cyan: fatty acid and phospholipid metabolism; light blue: biosynthesis of cofactors, prosthetic groups and carriers; aquamarine: central intermediary metabolism; royal blue: energy metabolism; pink: transport and binding proteins; dark orange: DNA metabolism and transcription; pale green: protein fate; tomato: regulatory functions and signal transduction; peach puff: cell envelope; pink: cellular processes; maroon: mobile and extrachromosomal element functions. The third circle represent RNA genes, including tRNAs (blue), rRNAs (red) and ncRNAs (orange). The fourth circle represents GC‐skew values [(G‐C)/(G+C)] with a windows size of 500 bp and a step size of 250 bp.

The genome of *N. helminthoeca* Oregon consists of a single double‐stranded circular chromosome spanning 884 232 bp, which is similar to those of *N. risticii* (Lin *et al*., 2009) and *N. sennetsu* (Dunning Hotopp *et al*., 2006; Table 2), and smaller than those of other members in the family Anaplasmataceae (approximately 1.0–1.5 Mbp; Dunning Hotopp *et al*., 2006). G+C content of *N. helminthoeca* genome is 41.7% (Table 2), which is similar to those of other *Neorickettsia* and *Anaplasma* spp., but greater than those (approximately 30%) of *Ehrlichia* spp. and *Wolbachia* spp. (Dunning Hotopp *et al*., 2006). The replication origin of *N. helminthoeca* (Fig. 2) was predicted based on one of the GC‐skew shift points, and the region between *hemE* (uroporphyrinogen decarboxylase, NHE_RS00005) and an uncharacterized phage protein (NHE_RS04160) as described in *N. risticii* (Lin *et al*., 2009), *N. sennetsu* (Dunning Hotopp *et al*., 2006) and other members in the family Anaplasmataceae (Ioannidis *et al*., 2007).

**Table 2:** Genome properties of *Neorickettsia* spp

Strains^a^	NHO	NRI	NSE
RefSeq	NZ_CP007481.1	NC_013009.1	NC_007798.1
Size (bp)	884 232	879 977	859 006
GC (%)	41.7	41.3	41.1
Protein	774	760	754
tRNA	33	33	33
rRNA	3	3	3
Other RNA	1	2	3
Pseudogene	16	11	2
Total Gene	827	808	795
Average gene length	865	842	803
Per cent Coding^b^	87.1	87.0	89.3
Assigned functions	548	534	540
Unknown functions	226 (29.2%)	226 (29.7%)	214 (28.4%)

**a.** Abbreviations: NHO, *N. helminthoeca* Oregon (data obtained from in this study); NRI, *N. risticii* Illinois (Lin *et al*., 2009); NSE, *N. sennetsu* Miyayama (Dunning Hotopp *et al*., 2006).

**b.** Percent coding includes tRNA, rRNA, small RNA and all protein‐coding genes.

The *N. helminthoeca* genome encodes one copy each of the 5S, 16S and 23S rRNA genes, which are separated in two loci with the 5S and 23S rRNA genes forming an operon (Fig. 2, red bars in third circle from outside) as in other sequenced members in the family Anaplasmataceae (Massung *et al*., 2002; Dunning Hotopp *et al*., 2006). Thirty‐three tRNA genes are identified, which include cognates for all 20 amino acids (Table 2). The numbers of tRNA genes are identical to other *Neorickettsia* spp., and similar to other members in the family Anaplasmataceae (Dunning Hotopp *et al*., 2006; Lin *et al*., 2009), or other bacteria with a single *rrn* operon (Lee *et al*., 2009).

With 827 protein‐ and RNA‐coding genes (Fig. 2, Table 2), *N. helminthoeca* has a smaller number of predicted genes as compared to other members in the family Anaplasmataceae, including *Ehrlichia*,* Anaplasma* and *Wolbachia* endosymbionts of insects or nematodes, each of which have around 1000 or more genes (Crossman, 2006; Dunning Hotopp *et al*., 2006; Lin *et al*., 2009). Among the 774 predicted protein‐coding open reading frames (ORFs), 548 genes are assigned with probable functions based on sequence similarity searches. Approximately 29% of the predicted ORFs (226 genes) in the genome are annotated as hypothetical proteins, either with conserved domains or of unknown functions (Table 3).

**Table 3:** Role category breakdown of protein‐coding genes in *Neorickettsia* species

Role category	NHO	NSE	NRI	Conserved^a^	Unique in NHO^a^
Amino acid biosynthesis	12	9	9	9	3
Biosynthesis of cofactor and vitamin	62	63	64	60	1
Cell envelope	45	31	31	28	17^b^
Cellular processes	43	36	36	37	3
Central intermediary metabolism	8	5	5	5	3
DNA metabolism	33	36	37	33	
Energy metabolism	76	74	76	74	
Fatty acid and phospholipid metabolism	22	22	22	22	
Mobile elements	4	4	4	4	
Protein fate	87	86	88	86	
Protein synthesis	107	104	105	102	2
Nucleotide biosynthesis	37	36	36	36	
Regulatory functions	10	10	10	10	
Signal transduction	5	5	5	5	
Transcription	23	23	24	22	
Transport and binding proteins	50	45	45	44	5
Unknown functions	226	226	214	160	55
Total Proteins^c^	774	760	754	668	89
Total Assigned Functions:	548	534	540	525	

**a.** Proteins conserved among three *Neorickettsia* spp. and specific to *N. helminthoeca* are based on 3‐way comparison analysis by BlastP (*E* < e^−10^).

**b.** *N. helminthoeca* encodes nearly complete pathways for peptidoglycan biosynthesis.

**c.** Certain proteins are assigned to multiple role categories.

### Comparison of genomic contents among *Neorickettsia* species

Previous studies have shown that *Anaplasma* spp. and *Ehrlichia* spp. have a single large‐scale symmetrical inversion (X‐alignment) near the replication origin, which is possibly mediated by duplicated *rho* genes (Dunning Hotopp *et al*., 2006; Frutos *et al*., 2007; Nene and Kole, 2009). In addition, *Anaplasma* and *Wolbachia* spp. have extensive genomic rearrangement throughout the genome (Wu *et al*., 2004; Dunning Hotopp *et al*., 2006). However, the synteny is highly conserved and such genomic rearrangements or a large‐scale inversion is not detected among *N. helminthoeca*,* N. sennetsu* and *N. risticii* (Fig. S1), and *rho* is not duplicated in three sequenced *Neorickettsia* spp. In agreement with the 16S rRNA divergence (Fig. 1), *N. helminthoeca* exhibits multiple synteny divergences from *N. risticii* and *N. sennetsu* (Fig. S1).

**Figure 3:**
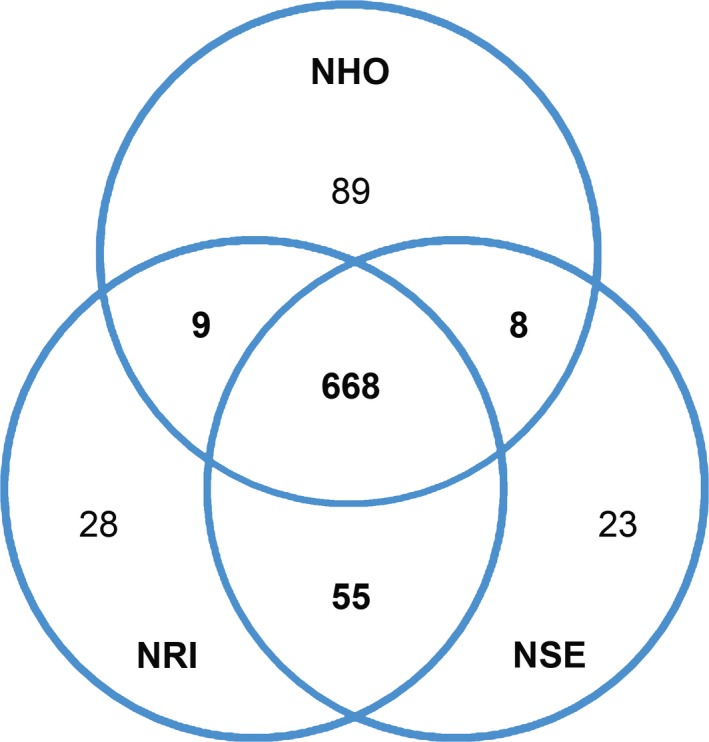
Numbers of protein orthologues shared among *Neorickettsia* spp. A Venn diagram was constructed showing the comparison of conserved and unique genes between *Neorickettsia* spp. as determined by reciprocal blastp algorithm (*E* < e^−10^). Numbers within the intersections of different circles indicate orthologue clusters shared by two or three organisms. Species indicated in the diagram are abbreviated as follows: *N. helminthoeca* (NHO), *N. sennetsu* (NSE), *N. risticii* (NRI).

To compare the genomic contents among *Neorickettsia* spp., two‐ and three‐way comparisons were performed using reciprocal blastp algorithm with *E*‐value < 1e^−10^, and homologous protein clusters were constructed. Three‐way comparison among *Neorickettsia* spp. showed that > 86% (668 of total 774 protein‐coding ORFs) of *N. helminthoeca* proteins are conserved with *N. risticii* and *N. sennetsu* (Table 3 and Table S1). The vast majority (> 78%, 525/668 ORFs) of these conserved proteins are associated with housekeeping functions and likely essential for *Neorickettsia* survival (Table 3). Two‐way comparisons revealed that *N. risticii* and *N. sennetsu* share an additional 55 conserved proteins, whereas *N. helminthoeca* shares very limited numbers of orthologues (< 10 proteins) with *N. risticii* or *N. sennetsu* (Fig. 3). The result of the two‐way and three‐way comparisons is consistent with the relationship of the species revealed through 16S rRNA‐based phylogeny and whole‐genome synteny analysis.

The three *Neorickettsia* spp. are transmitted by distinct trematodes and cause severe diseases at high mortality in different mammalian hosts (Table 1; Cordes *et al*., 1986; Dutta *et al*., 1988; Rikihisa *et al*., 1991, 2004, 2005; Gibson and Rikihisa, 2008; Lin *et al*., 2009). We, therefore, analysed the species‐specific genes based on the two‐ and three‐way comparisons. There are 89 species‐specific proteins in *N. helminthoeca* as compared to 28 and 23 in *N. risticii* and *N. sennetsu* respectively (Tables S2–S4). Of the genes unique to *N. helminthoeca*, more than half of them (50/89 ORFs) are hypothetical proteins without assigned functions (Table S2). Among the *N. helminthoeca*‐specific proteins with assigned functions, ~38% (15/39 ORFs) are involved in peptidoglycan biosynthesis that are absent in *N. risticii* and *N. sennetsu* (Table S2 and Fig. 5), and six proteins are categorized as transporters for iron and other substrates (Table S2). The genomic loci encoding these unique ORFs are distributed throughout *N. helminthoeca* genome and not clustered in certain islands (Fig. 2, second circle from outside). Blast searches using these *N. helminthoeca*‐specific proteins against NCBI protein database excluding *Neorickettsia* spp. showed that only 29 of them match to proteins in other genera, and the majority of them (19, 65.5%) belong to α‐proteobacteria (Table S2). However, whether these proteins are the results of horizontal gene transfer or mutations/deletions from the ancestors of *Neorickettsia* spp. remains to be determined.

### Metabolism

**Figure 4:**
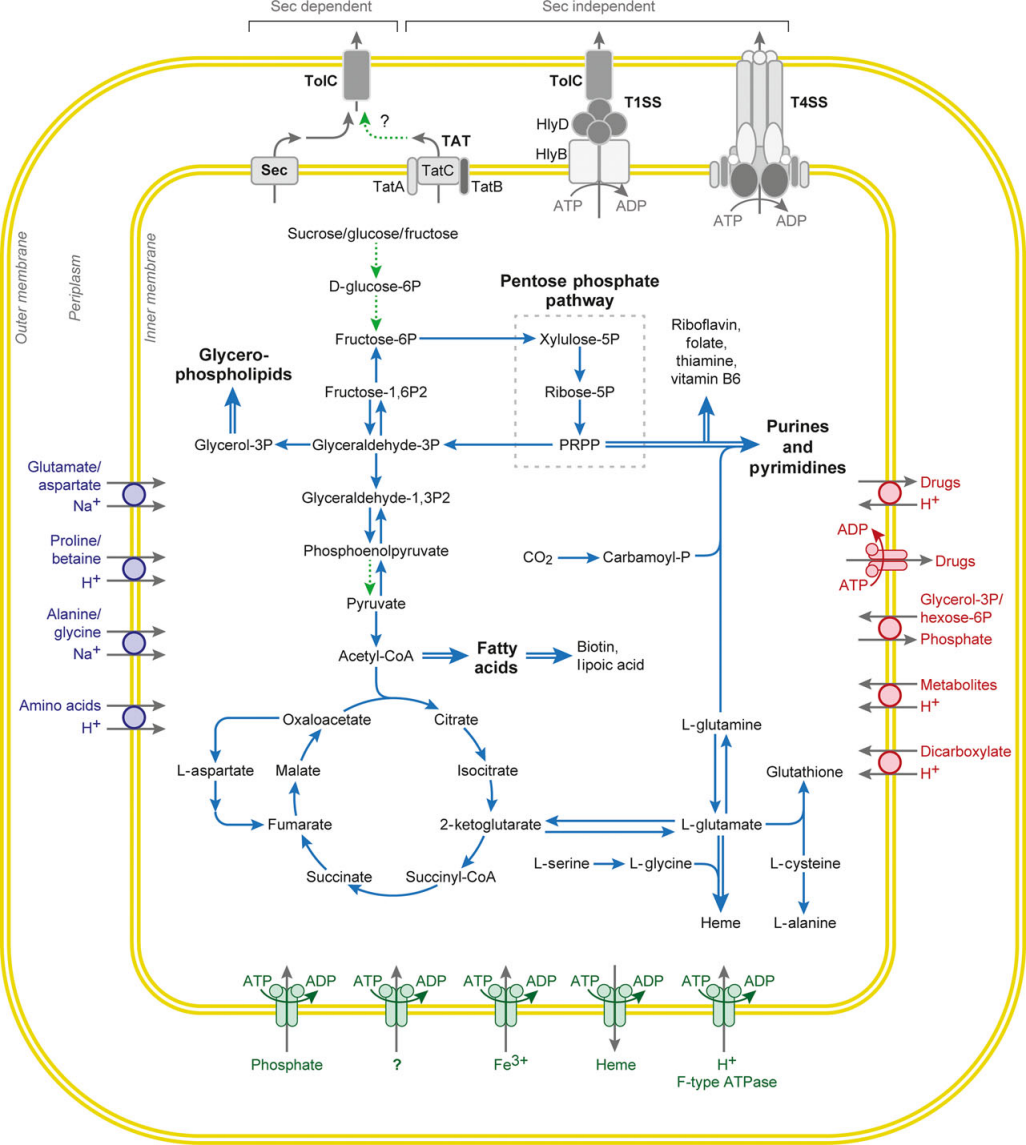
Major metabolic pathways and secretion systems of *N. helminthoeca*. *N. helminthoeca* encodes pathways for aerobic respiration, including the tricarboxylic acid (TCA) cycle and the electron transport chain, but it is unable to use glucose, fructose, or fatty acids directly as a carbon or energy source. *N. helminthoeca* can synthesize very limited amino acids, but can synthesize most vitamins/cofactors, fatty acids and certain phospholipids, and encodes complete pathways for *de novo* purine and pyrimidine biosynthesis. Putative transporters were analysed by TransAAP (http://www.membranetransport.org/), and secretion systems were drawn as described in Results. Solid lines, pathways present; dashed lines, pathways absent; double lines, multiple steps involved. Graph was modified from KEGG pathways, Dunning Hotopp *et al*. (2006) and Gillespie *et al*. (2015).

Except for peptidoglycan biosynthesis, most metabolic pathways, transcription, translation and regulatory functions, are highly conserved in *N. helminthoeca* compared to *N. sennetsu* and *N. risticii* (summarized in Fig. 4, Tables 3 and S1; Dunning Hotopp *et al*., 2006; Lin *et al*., 2009).

#### Central metabolic pathways

Analysis of the metabolic pathways based on Kyoto Encyclopedia of Genes and Genomes (KEGG, http://www.kegg.jp) and BioCyc (http://biocyc.org/) indicates that, similar to other members in the family Anaplasmataceae, *N. helminthoeca* encodes pathways for aerobic respiration, including the tricarboxylic acid (TCA) cycle and the electron transport chain, but it is unable to use glucose, fructose, or fatty acids directly as a carbon or energy source, as essential enzymes for the utilization of these substrates, such as hexokinases, the first enzyme in the glycolysis pathway that converts glucose to glucose 6‐phosphate, and pyruvate kinase that converts phosphoenolpyruvate to pyruvate, are not identified (Fig. 4). It is likely that *N. helminthoeca* can synthesize ATP from glutamine as *N. risticii*,* N. sennetsu*, or *E. chaffeensis* does (Weiss *et al*., 1989; Cheng *et al*., 2014), as it encodes carbamoyl phosphate synthase (carA/B, NHE_RS00875/NHE_RS02090) and bifunctional glutamate synthase β subunit/2‐polyprenylphenol hydroxylase (GS/PH, NHE_RS02780). These enzymes can convert glutamine to ammonia and glutamate (Fig. 4), and glutamate can be further converted by glutamate dehydrogenase (NHE_RS02165) to 2‐ketoglutarate, which enters the TCA cycle for energy production.

#### Amino acids, nucleotides, fatty acids and cofactor biosynthesis

Like other *Neorickettsia*,* Ehrlichia* and *Anaplasma* spp. (Dunning Hotopp *et al*., 2006; Lin *et al*., 2009), *N. helminthoeca* synthesizes very limited amino acids including alanine, aspartate, glycine, glutamate and glutamine (Fig. 4 and Table S5). As they are converted from other amino acids or metabolic intermediates, *N. helminthoeca* must transport most amino acids from its host as discussed further below (Table S7). However, as other members of the family Anaplasmataceae, analysis of KEGG pathways showed that most enzymes are identified for the biosynthesis of fatty acids and certain phospholipids, including phosphatidylglycerol, phosphatidylserine, phosphatidylethanolamine and myo‐inositol‐phosphates (Fig. 4).

Similar to all other sequenced members of Anaplasmataceae (Dunning Hotopp *et al*., 2006), *N. helminthoeca* encodes a non‐oxidative pentose‐phosphate pathway that utilizes glyceraldehyde‐3‐phosphate to produce pentose for nucleotide and cofactor biosynthesis. Accordingly, *N. helminthoeca* encodes complete pathways for *de novo* purine and pyrimidine biosynthesis and is capable of synthesizing most vitamins or cofactors, such as biotin, folate, FAD, NAD and protoheme (Fig. 4 and Table S6). Overall, *N. helminthoeca* encodes large number of genes involved in the biosynthesis of cofactors, vitamins and nucleotides (17.2%, 133 of total 774 protein‐coding ORFs), similar to other members of Anaplasmataceae like *Ehrlichia* (13.4%, 149/1115 ORFs in *E. chaffeensis*), *Anaplasma* (10.6%, 145/1370 ORFs in *A. phagocytophilum*; Dunning Hotopp *et al*., 2006) and *Wolbachia* endosymbionts of the insects or nematodes (9.4%, 120/1271 in *Wolbachia pipientis* wMel; Foster *et al*., 2005; Brownlie *et al*., 2009). Unlike tick‐borne members in the family Anaplasmataceae (*Ehrlichia* and *Anaplasma* spp.), *Neorickettsia* spp. are maintained throughout the life cycle of the trematodes (Greiman *et al*., 2016; Fig. 1). The presence of these biosynthesis pathways suggests that *N. helminthoeca* do not need to compete with the host for the essential vitamins and nucleotides, which is likely beneficial for their survival especially in invertebrate hosts.

#### Transporters and porins

To compensate for the incomplete biosynthesis or metabolic pathways, the *N. helminthoeca* genome encodes several orthologues involved in cytoplasmic membrane transport systems that can supply the necessary amino acids, metabolites and ions, as analysed by TransAAP (Transporter Automatic Annotation Pipeline, http://www.membranetransport.org/; Fig. 4 and Table S7; Ren and Paulsen, 2007; Ren *et al*., 2007). Transporters for acetyl‐CoA involved in many metabolic pathways and glycerol‐3‐phosphate in phospholipid biosynthesis are identified in *N. helminthoeca* genome (Table S7). Transport systems for phosphates (pstA/B/C/S), cations, anions, organic ions and multidrug resistance pumps are also present (Table S7). Putative amino acid transporters for alanine, glycine, proline and dicarboxylate amino acids (glutamate or aspartate family) can be found (Table S7). However, as very few amino acids can be synthesized in *N. helminthoeca*, more transporters are required; it is possible that some ATP‐binding cassette (ABC)‐type transporters with no assigned functions or porins discussed below could act as transporters for amino acids as well as metabolites for protein synthesis and energy production. Orthologues of most identified transporters are conserved in *N. risticii* and *N. sennetsu* genomes (Tables S1 and S7), except for few *N. helminthoeca*‐specific transporters listed in Table S2. Unlike *Rickettsia* spp. (Winkler, 1976), but similar to all other sequenced members of the Family Anaplasmataceae, *N. helminthoeca* does not encode translocases for ATP (ATP:ADP antiporters) or NADH, so it likely relies on its own ATP production or encodes unique ATP acquisition mechanisms.

Gram‐negative bacteria also express porins spanning their outer membranes that enable the transport of hydrophilic and large molecules, such as amino acids, sugars and other nutrients (Nikaido, 2003). Similar to other members of the Anaplasmataceae that have limited capabilities of amino acids biosynthesis, intermediary metabolism and glycolysis, nutrient uptake in these bacteria necessitates pores or channels in the bacterial outer membrane (Huang *et al*., 2007; Kumagai *et al*., 2008; Gibson *et al*., 2010). Previous studies have determined that the major outer membrane proteins, including *A. phagocytophilum* P44s (Huang *et al*., 2007), *E. chaffeensis* P28/OMP‐1F (Kumagai *et al*., 2008) and *N. sennetsu* P51 (Gibson *et al*., 2010), possess porin activities as determined by a proteoliposome swelling assay, which allow the diffusion of l‐glutamine, the monosaccharides arabinose and glucose, the disaccharide sucrose and even the tetrasaccharide stachyose. *N. helminthoeca* encodes a P51 protein (NHE_RS00965) that shares 60% amino acid sequence similarity with *N. sennetsu* P51 protein (Fig. 6A). Prediction of the two‐dimensional structure of *N. helminthoeca* P51 using PRED‐TMBB (http://biophysics.biol.uoa.gr/PRED-TMBB/; Bagos *et al*., 2004) showed that P51 protein contains 18 transmembrane domains with a discrimination value of 2.949 (Fig. S2), suggesting that it is a β‐barrel protein localized to the outer membrane similar to *N. sennetsu* P51 (Gibson *et al*., 2010). Therefore, it is likely that *N. helminthoeca* P51 can function as a porin for nutrient uptake from the host.

#### DNA, RNA, protein synthesis and DNA repair

*Neorickettsia helminthoeca* encodes proteins necessary for DNA replication, RNA synthesis and degradation and ribosomal proteins. Although *N. helminthoeca* encodes proteins required for homologous recombination, including RecA/RecF (but not RecBCD) pathways (Lin *et al*., 2006) and RuvABC complexes for Holliday junction recombination as other members of the family Anaplasmataceae (Table S8), it has the least amount of enzymes involved in DNA repair compared to other members of the family Anaplasmataceae including *N. sennetsu* and *N. risticii* (seven in *N. helminthoeca* vs. nine in *N. sennetsu*, 12 in *E. chaffeensis* and 13 in *A. phagocytophilum*, Table S8; Dunning Hotopp *et al*., 2006; Lin *et al*., 2009). *N. helminthoeca* lacks most genes required for mismatch repair, nucleotide excision repair (NER, such as *uvrABC* for UV‐induced DNA damage), various glycosylases for base excision repair (BER) and DNA photolyases, which is an alternative mechanism to repair UV‐damaged DNA identified in *E. chaffeensis*,* A. phagocytophilum* and *N. risticii* (Dunning Hotopp *et al*., 2006; Lin *et al*., 2009).

### Pathogenesis

Although SPD was recognized more than two centuries ago, the causative agent *N. helminthoeca* was only stably cultured in a canine cell line in 1990 (Rikihisa *et al*., 1991), and there is little information available regarding the molecular determinants of *N. helminthoeca* to invade and cause severe disease in canine hosts. Here, we analysed genes and pathways that are potentially involved in *N. helminthoeca* pathogenesis, including protein secretion systems, two‐component/one‐component regulatory systems, *N. helminthoeca‐*specific genes and putative membrane proteins or lipoproteins.

#### Protein secretion systems

Two major pathways exist to secrete proteins across the cytoplasmic membrane in bacteria. The general *Sec*retion route, termed Sec‐pathway, catalyses the transmembrane translocation of proteins in their unfolded conformation, whereupon they fold into their native structure at the *trans*‐side of the membrane (Natale *et al*., 2008). All major components for the Sec‐dependent pathway are identified, including signal recognition particle (SRP) protein, SRP‐docking protein FtsY, the cytosolic protein‐export chaperone SecB, peripheral associated ATP‐dependent motor protein SecA, membrane‐embedded protein conducting channel SecYEG, periplasmic protein YajC that involved in preprotein translocase activity and the membrane complex SecDF that enhances proton motive force (Fig. 4 and summarized under role category ‘Protein fate’ in Table S1). In addition, common chaperones are identified in *N. helminthoeca* genome, including *groEL*,* groES*,* dnaK*,* dnaJ*,* hscA/B*,* grpE* and *htpG* (summarized under role category ‘Protein fate’ in Table S1).

*T*win‐*a*rginine *t*ranslocation (Tat)‐pathway, which consists of the TatA, TatB and TatC proteins, can transport folded proteins across the bacterial cytoplasmic membrane by recognizing N‐terminal signal peptides harbouring a distinctive twin‐arginine motif (Lee *et al*., 2006; Sargent *et al*., 2006). All genes encoding Tat apparatus are identified in the *N. helminthoeca* genome (*tatA*/NHE_RS02000, *tatB*/NHE_RS02160 and *tatC*/NHE_RS00490; Fig. 4 and Table S6; Gillespie *et al*., 2015). However, despite the presence of Tat system, no protein substrate containing a putative Tat signal peptide can be identified in *N. helminthoeca* using both TAT‐FIND (http://www.cbs.dtu.dk/services/TatP/; Bendtsen *et al*., 2005) and PRED‐TAT (http://www.compgen.org/tools/PRED-TAT) algorithms (Bagos *et al*., 2010). Gillespie *et al*. (2015) reported only a single Tat substrate (PetA) in *Rickettsia* and suggested that could be due to the substantial differences in signal peptides of Tat substrates in the obligate intracellular bacteria.

Extracellular secretion of various virulence factors across the bacterial cell envelope is one of the major mechanisms by which pathogenic bacteria alter host cell functions, thus enhancing survival of the bacteria and damaging hosts. At least six distinct extracellular protein secretion systems, referred to as type I‐VI secretion systems (T1SS‐T6SS; Papanikou *et al*., 2007; Costa *et al*., 2015), have been classified in Gram‐negative bacteria that secrete effector molecules across two lipid bilayers and the periplasm. Except for T2SS, all double‐membrane‐spanning secretion systems (T1SS, T3SS, T4SS and T6SS) use a one‐step mechanism to transport substrates directly from the bacterial cytoplasm into the extracellular space or into a target cell (Costa *et al*., 2015). Bioinfomatic analysis shows that, similar to all other sequenced members of the family Anaplasmataceae, *N. helminthoeca* genome encodes both T1SS and T4SS for secretion of proteins across the membranes, but it lacks homologs of T2SS, T3SS, T5SS or T6SS components (Fig. 4; Henderson *et al*., 2004; Cianciotto, 2005; Bingle *et al*., 2008). T1SS, a Sec‐independent ATP‐driven ABC transporter system that bypasses the periplasm, is capable of transporting target proteins carrying a C‐terminal uncleaved secretion signal across both inner and outer membranes and into the extracellular medium (Delepelaire, 2004). All of the three components of T1SS, including an inner membrane ATP‐binding cassette (ABC) transporter HlyB (NHE_RS00175), a periplasmic membrane fusion protein (MFP) HlyD (NHE_RS04020) and an outer membrane channel protein TolC (NHE_RS03400) are identified in the *N. helminthoeca* genome (Fig. 4, Table S1 and S6). A previous study reported that several tandem repeat proteins (TRP120, TRP47 and TRP32/VLPT) are T1SS substrates of *E. chaffeensis* using an *E. coli* T1SS surrogate system (Wakeel *et al*., 2011). Current analysis using the T‐REKS algorism (Jorda and Kajava, 2009) identified several tandem repeat‐containing proteins (not homologous to *E. chaffeensis* TRPs) like VirB6 and SSAs in all three sequenced *Neorickettsia*; however, whether these proteins are also secreted by T1SS is unknown (Table S9; Dunning Hotopp *et al*., 2006; Lin *et al*., 2009).

T4SS can translocate bacterial effector molecules into host cells, thus often plays a key role in pathogenesis of Gram‐negative host‐associated bacteria (Cascales and Christie, 2003; Backert and Meyer, 2006; Gillespie *et al*., 2010; Christie *et al*., 2014). In several intracellular bacteria including the family Anaplasmataceae such as *E. chaffeensis* and *A. phagocytophilum*, the T4SS is critical for survival and replication inside host cells, by inducing autophagy for nutrient acquisition and inhibition of host cell apoptosis (Niu *et al*., 2006; Niu *et al*., 2010; Liu *et al*., 2012; Niu *et al*., 2012; Lin *et al*., 2007; Lin *et al*., 2016). In the *N. helminthoeca* genome, we identified a T4SS encoded by *virB/D* genes distributed in four separate loci. The organization of *virB/D* gene clusters is conserved among *Neorickettsia* spp. as with other Anaplasmataceae, with duplicated genes of *virB4*,* virB8* and *virB9*, and multiple copies of *virB2* and *virB6* genes (Tables S1 and S6).

Subcellular fractionation and functional studies have demonstrated that VirB2 is the major pilus component of T4SS extracellular filaments (Cascales and Christie, 2003; Backert and Meyer, 2006). Our previous study has confirmed that *N. risticii* VirB2 was localized at the opposite poles on the bacterial surface (Lin *et al*., 2009), suggesting that VirB2 might serve as secretion channels for the T4SS apparatus like that of *Agrobacterium* (Cascales and Christie, 2003), and play critical roles in mediating the interaction with host cells. Analysis of *N. helminthoeca* genome reveals three copies of *virB2* upstream of *virB4*, whereas *N. risticii* and *N. sennetsu* encode two *virB2* genes (Table S6; Lin *et al*., 2009). Alignment of VirB2 protein sequences indicates that VirB2s of *Neorickettsia* spp. are closely related to those of other α‐proteobacteria like *Rickettsia*,* Agrobacterium* and *Caulobacter*, but are phylogenetically distinct from VirB2s of *E. chaffeensis* and *A. phagocytophilum* that form a separate clade (Fig. S3; Gillespie *et al*., 2009, 2010). The different numbers of *virB2* genes and distinct differences in phylogenetic trees of VirB2 from 16S rRNA gene suggest that *virB2* genes might undergo lineage‐specific mutations, duplications, or deletions (Gillespie *et al*., 2010).

#### Two‐component regulatory systems

Two‐component regulatory systems (TCRS) are signal transduction systems that allow bacteria to sense and respond rapidly to changing environmental conditions (Mitrophanov and Groisman, 2008; Wuichet *et al*., 2010). TCRS consists of a sensor histidine protein kinase that responds to specific signals, and a cognate response regulator. Phosphorylation of a response regulator by a cognate histidine kinase changes the biochemical properties of its output domain, which can participate in DNA binding and transcriptional control, perform enzymatic activities, bind RNA, or engage in protein–protein interactions (Gao *et al*., 2007). TCRS plays a key role in controlling virulence responses in a wide variety of bacterial pathogens (Dorman *et al*., 2001; Mitrophanov and Groisman, 2008), including *E. chaffeensis* and *A. phagocytophilum* in the family Anaplasmataceae, which encode three pairs of TCRS, including CckA/CtrA, PleC/PleD and NtrX/NtrY (Cheng *et al*., 2006; Cheng *et al*., 2011; Kumagai *et al*., 2006; Kumagai *et al*., 2011).

Computational analysis reveals that the three sequenced *Neorickettsia* spp. encode two pairs of TCRS: CckA/CtrA and PleC/PleD (Table S6). The histidine kinase CckA/response regulator CtrA pair, identified only in α‐proteobacteria, also have been demonstrated to coordinate multiple cell cycle events at the transcriptional level in *E. chaffeensis* to regulate bacterial developmental cycle (Cheng *et al*., 2011). Different from *Ehrlichia* and *Anaplasma*, the three *Neorickettsia* spp. encode two copies of PleC histidine kinase (NHE_RS00035/NHE_RS02255, Tables S1 and S6) and a one‐component signal transduction protein, an EAL domain protein (NHE_RS01830; Fig. S4; Ulrich and Zhulin, 2007; Lin *et al*., 2009; Romling, 2009; Ulrich and Zhulin, 2010; Lai *et al*., 2009). The response regulator PleD (NHE_RS02155) can function as diguanyl cyclase that produces cyclic diguanylate (c‐di‐GMP) to regulate cell surface adhesiveness like biofilm or extracellular matrix formation (Tischler and Camilli, 2004), whereas EAL domain protein can function as a diguanylate phosphodiesterase (PDE) that converts c‐di‐GMP to GMP. They likely function synergistically to regulate surface adhesiveness of *Neorickettsia*, resulting much smaller morulae sizes and more dispersed bacterial colonies compared to *Ehrlichia* and *Anaplasma* (Rikihisa, 1991a). In addition, *Neorickettsia* spp. do not encode genes for NtrY/NtrX, which are thought to be involved in nitrogen metabolism and regulation of nitrogen fixation genes like *glnA* that encodes a glutamine synthase as in *E. chaffeensis* (Cheng *et al*., 2014). Despite this, *N. helminthoeca* encodes GlnA (NHE_RS01490) and ABC dicarboxylate amino acid transporters (NHE_RS00770) that are predicted to take up glutamine (Table S7) similar to *E. chaffeensis* (Cheng *et al*., 2014), suggesting regulation of nitrogen metabolism in *Neorickettsia* spp. is different from *Ehrlichia* and *Anaplasma* spp.

#### One‐component regulatory systems and transcriptional regulations

One‐component regulatory systems consist of a single protein containing both input and output domains, but lack the phospho‐transfer domains of TCRS, and carry out signalling events in prokaryotes (Ulrich *et al*., 2005; Ulrich and Zhulin, 2007, 2010). This study found that compared to *Ehrlichia* and *Anaplasma*, the three *Neorickettsia* spp. encode more proteins in one‐component systems (indicated by asterisks in Fig. S4, based on Microbial Signal Transduction Database at http://mistdb.com; Ulrich *et al*., 2005). Other than an EAL domain protein described above and an HD domain containing deoxyguanosinetriphosphate triphosphohydrolase protein (NHE_RS01895), most one‐component regulatory systems of *N. helminthoeca* as well as *N. risticii and N. sennetsu* are predicted to be DNA‐binding transcriptional regulators (Fig. S4, Table S1).

Perhaps due to the relatively homoeostatic intracellular environment of the eukaryotic host cells, members of the order Rickettsiales and Chlamydiaceae have a small number of transcriptional regulators. *N. helminthoeca* as all other members of the family Anaplasmataceae encodes only two sigma factors: the essential RNA polymerase sigma‐70 factor (RpoD, NHE_RS01300) responsible for most RNA synthesis in exponentially growing cells, and sigma‐32 factor (RpoH, NHE_RS01445) responsible for expression from heat‐shock promoters.

*Neorickettsia helminthoeca* encodes a putative transcriptional regulator NhxR (*N. helminthoeca* expression regulator), a 12.5‐kDa DNA‐binding protein (NHE_RS00155) that has 90% amino acid identity with *N. risticii* NrxR (NRI_RS00145) and *N. sennetsu* NsxR (NSE_RS00160). NhxR homologues, *A. phagocytophilum* ApxR and *E. chaffeensis* EcxR have shown to regulate the expression of P44 outer membrane proteins and the T4SS respectively (Wang *et al*., 2007b,a; Cheng *et al*., 2008). The other putative transcriptional regulator Tr1 (NHE_RS00915) is homologous to *A. phagocytophilum* and *E. chaffeensis* Tr1, which is regulated by ApxR in *A. phagocytophilum* and located at the upstream of the tandem genes encoding the major outer membrane proteins (OMPs), like Omp‐1/Msp‐2/P44 expression loci in *A. phagocytophilum* (Lin *et al*., 2004) or P28/Omp‐1 gene clusters in *E. chaffeensis* (Ohashi *et al*., 2001; Wang *et al*., 2007a; Rikihisa, 2010). However, Tr1 in *N. helminthoeca*,* N. risticii*, or *N. sennetsu* is not located at upstream of any of genes encoding the major OMPs of *N. helminthoeca* including P51, SSA or NSPs (Table 4).

**Table 4:** Putative outer membrane proteins of *Neorickettsia helminthoeca* a

Locus ID	Gene symbol	Protein name	MW (kDa)
*Molecular Characterization*			
NHE_RS00965	*p51*	P51 Gram‐negative porin family protein	51.6
NHE_RS03715	*nsp1*	*Neorickettsia* surface protein 1	27.6
NHE_RS03720	*nsp2*	*Neorickettsia* surface protein 2	33.7
NHE_RS03725	*nsp3*	*Neorickettsia* surface protein 3	24.7
NHE_RS03855	*ssa*	Strain‐specific surface antigen	35.5
*pSort‐B Prediction*			
NHE_RS00040		Conserved hypothetical protein	46.3
NHE_RS01885		Conserved hypothetical protein	73.4
NHE_RS03040	*yaeT*	Outer membrane protein assembly complex, YaeT protein	82.9
NHE_RS03940	*bamD*	BamD lipoprotein	26.5

**a.** Location of outer member proteins is predicted by the pSort‐B algorithm (http://psort.org/psortb). Other putative OMPs (P51, NSP1/2/3 and SSA) are determined by homology searches to *N. risticii* and *N. sennetsu* protein database using blastp.

The present study identified several other *N. helminthoeca* DNA‐binding regulators, which are conserved in *N. risticii* and *N. sennetsu* (Fig. S4 and Table S1; Lin *et al*., 2009). These proteins include (i) a putative transcriptional regulator (NHE_RS02120) containing a helix–turn–helix motif and a peptidase S24 LexA‐like family domain that are likely involved in the SOS response leading to the repair of single‐stranded DNA, (ii) a DNA‐binding protein with a putative transposase domain (NHE_RS04205), (iii) a transcriptional regulator of the MerR (mercuric resistance operon regulator) family (NHE_RS01200), and (iv) an Rrf2 family transcriptional regulator with aminotransferase class V domain (NHE_RS01260; Fig. S4). Functions of any of them remain to be studied.

#### Ankyrin domain proteins

Ankyrin repeat domains (Ank), found predominantly in eukaryotic proteins, are known to mediate protein‐protein interactions involved in a multitude of host processes, including cytoskeletal motility, tumour suppression and transcriptional regulation (Bennett and Baines, 2001; Mosavi *et al*., 2004). Compared to free‐living bacteria, Ank proteins are enriched in facultative and obligate intracellular bacteria of eukaryotes (Jernigan and Bordenstein, 2014). Several studies have shown that the ankyrin repeat‐containing protein AnkA of *A. phagocytophilum* is secreted into host cells by the T4SS and plays an important role in facilitating intracellular infection by activating the Abl‐1 protein tyrosine kinase, interacting with the host tyrosine phosphatase SHP‐1, or regulation of host cell transcription (IJdo *et al*., 2007; Lin *et al*., 2007; Garcia‐Garcia *et al*., 2009). In *E. chaffeensis*, AnkA homologue Ank200 is translocated into the host cell nucleus through a T1SS‐dependent manner and binds to Alu elements and numerous host proteins (Zhu *et al*., 2009; Wakeel *et al*., 2011). Four ankyrin repeat‐containing proteins were identified in the *N. helminthoeca* genome (four in *N. risticii* and three in *N. sennetsu*; Table S6). Phylogenetic analysis indicated that *N. helminthoeca* encodes one Ank protein (NHE_RS00105) that is clustered with *E. chaffeensis* T1SS substrate Ank200 (11.6% amino acid similarities; Wakeel *et al*., 2011) and less related to *A. phagocytophilum* T4SS substrate AnkA (8.6% amino acid similarities; Lin *et al*., 2007; Fig. S5). However, whether any of these ankyrin repeat‐containing proteins of *Neorickettsia* spp. can be secreted into host cytoplasm by the T1SS or T4SS and regulate host cell functions remain to be determined.

#### Iron uptake and storage

Iron is an essential element for almost all living organisms and serves as a cofactor in key metabolic processes including energy generation, electron transport and DNA synthesis (Skaar, 2010). This study found that the three *Neorickettsia* spp., *E. chaffeensis* and *A. phagocytophilum* encode proteins for iron transport across inner membranes, including periplasmic Fe^3+^‐binding protein FbpA (NHE_RS00045), cytoplasmic membrane permease component FbpB (NHE_RS01265) and cytoplasmic ABC transporter FbpC (PotC, NHE_RS01995; Table S1). However, homologues to known bacterial siderophore and outer membrane receptors for iron or chelated iron are not identified in these bacteria, suggesting that they might use a unique system to bind and uptake iron from their host. Infection of *N. risticii*,* N. sennetsu* and *E. chaffeensis*, but not *A. phagocytophilum*, are inhibited by an intracellular labile iron chelator deferoxamine (Park and Rikihisa, 1992; Barnewall and Rikihisa, 1994; Barnewall *et al*., 1999), suggesting that these bacteria may utilize different iron‐uptake system to obtain iron from the host. Unlike *E. chaffeensis* and *A. phagocytophilum*, current analysis found that the three *Neorickettsia* spp. encode a bacterioferritin (NHE_RS01470; Table S1, under role category ‘Transport and binding proteins’), which can capture soluble but potentially toxic Fe^2+^ by compartmentalizing it in the form of a bioavailable ferric mineral inside the protein's hollow cavity. In the family Anaplasmataceae, bacterioferritin is also found in the *Wolbachia* endosymbiont of insects or nematode (Kremer *et al*., 2009). This could be due to differences in their life cycle and invertebrate host: the entire life cycles of *Neorickettsia* and *Wolbachia* spp. are within trematodes, insects, or nematodes with limited labile iron pools, whereas *Ehrlichia* and *Anaplasma* live within mammalian blood cells and tick vectors fed on blood rich in iron (Fig. 1).

### Cell wall components

#### Lipopolysaccharide and peptidoglycan

*N. helminthoeca* lacks all genes encoding lipopolysaccharide (LPS) biosynthesis pathway including lipid A (the core component of LPS) as other sequenced members of the family Anaplasmataceae (Lin and Rikihisa, 2003; Dunning Hotopp *et al*., 2006; Lin *et al*., 2009), including the recently sequenced *NFh* (McNulty *et al*., 2017). Although few genes involved in LPS biosynthesis were identified in the draft genome of *Candidatus* ‘X. pacificiensis’, it was not expected to possess a functional LPS biosynthesis pathway (Kwan and Schmidt, 2013).

**Figure 5:**
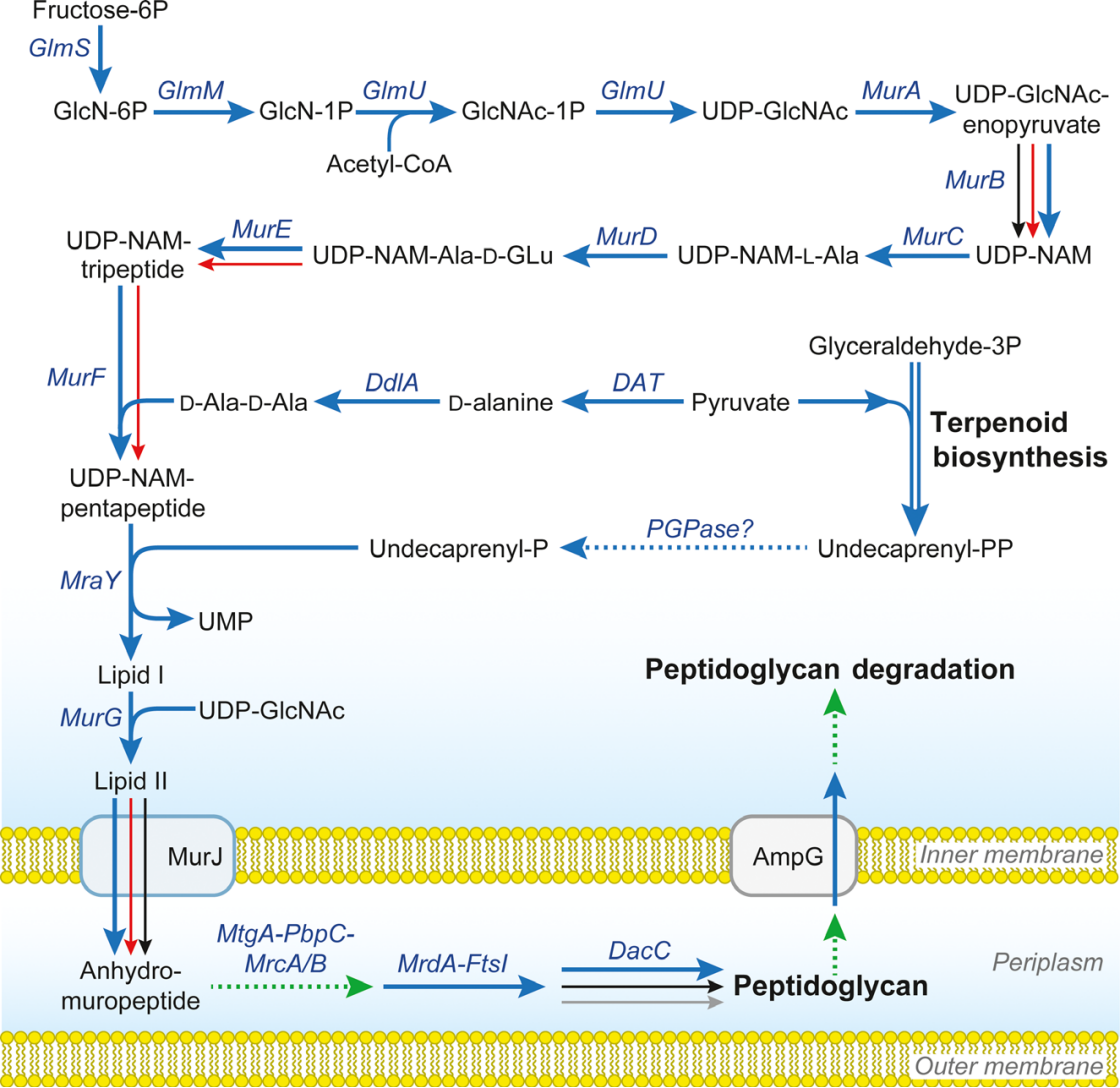
Genes involved in peptidoglycan biosynthesis in selected members of the family Anaplasmataceae. Biosynthesis pathways of peptidoglycan for *N. helminthoeca*,* N. risticii*,* N. sennetsu*,* E. chaffeensis*,* E. ruminantium*,* A. phagocytophilum*,* A. marginale* and *Wolbachia* wMel endosymbiont of *Drosophila melanogaster* were downloaded from KEGG database (http://www.genome.jp) and analysed. *N. helminthoeca*,* A. marginale* and *Wolbachia* wMel encode nearly all genes for peptidoglycan biosynthesis pathways (blue arrows), except that *A. marginale* and *Wolbachia* wMel lacks genes for the biosynthesis of d‐Ala‐d‐Ala. In addition, all members in the family Anaplasmataceae encode terpenoid biosynthesis pathways like isopentenyl‐, farnesyl‐ and geranyl‐diphosphate; however, only *Neorickettsia* and *Wolbachia* spp. encode undecaprenyl diphosphate (Und‐PP) synthase (UppS) to produce Und‐PP. *N. helminthoeca* encodes two PGPases (NHE_RS00895 and NHE_RS01205) that might produce Und‐P from Und‐PP. Genes present in *N. risticii* and *N. sennetsu*, red arrows; *A. phagocytophilum*, black arrows; *E. chaffeensis* and *E. ruminantium*, grey arrow. Dashed green lines, genes absent in all bacteria analysed; dashed blue line, potential pathway present. Diagram was modified from KEGG pathways and Gillespie *et al*. (2010). Abbreviations: GlcN, d‐Glucosamine; GlcNAc, N‐Acetyl‐α‐d‐glucosamine; UDP‐NAM, UDP‐N‐acetylmuramate; Undecaprenyl‐PP (Und‐PP), di‐trans, poly‐cis‐undecaprenyl diphosphate; *m*DAP, meso‐2,6‐diaminopimelate; UDP‐NAM‐Tripeptide, UDP‐NAM‐l‐Ala‐d‐Glu‐*m*DAP; UDP‐NAM‐Pentapeptide, UDP‐NAM‐l‐Ala‐d‐Glu‐*m*DAP‐d‐Ala‐d‐Ala; Lipid I, Und‐PP‐NAM‐l‐Ala‐d‐Glu‐*m*DAP‐d‐Ala‐d‐Ala; Lipid II, Und‐PP‐NAM‐(GlcNAc)‐l‐Ala‐d‐Glu‐*m*DAP‐d‐Ala‐d‐Ala; DAT, d‐alanine transaminase; PGPase, phosphatidylglycerophosphatase.

Interestingly, nearly all genes involved in peptidoglycan biosynthesis are identified in *N. helminthoeca*,* A. marginale* and *Wolbachia* wMel (endosymbiont of insect *Drosophila melanogaster*) or wBm (endosymbiont of nematode *Brugia malayi*) in the family Anaplasmataceae. On the contrary, only a very limited numbers of genes in peptidoglycan biosynthesis are present in the genomes of *N. risticii*,* N. sennetsu*,* E. chaffeensis*,* E. ruminantium* and *A. phagocytophilum* (Fig. 5). This suggests that the ancestors of the family Anaplasmataceae have undergone independent but parallel loss of the peptidoglycan biosynthetic genes and genome reduction.

Analysis of *N. helminthoeca* genome suggests that it can perform *de novo* synthesis of d‐Ala‐d‐Ala from pyruvate, meso‐2,6‐diaminopimelate (*m*DAP) from l‐Asp and undecaprenyl diphosphate (Und‐PP) through terpenoid biosynthesis pathways (isopentenyl‐ and farnesyl‐diphosphate). Although undecaprenyl diphosphatase like *E. coli* phosphatidylglycerophosphatase B (PGPase B, PgpB) homologue was not found in *N. helminthoeca*,* N. helminthoeca* encodes two putative PgpA superfamily proteins (NHE_RS00895 and NHE_RS01205) that might function as PGPases to produce Und‐P from Und‐PP. A flippase (MurJ, NHE_RS02395) that transports anhydromuropeptide into periplasm was also identified in *N. helminthoeca* (Fig. 5).

The incorporation of anhydromuropeptide subunits into the murein sacculus requires multiple enzymes like MtgA, MrcA/B, FtsI (PbpB), PbpC, MrdA (Pbp2), MrdB, DacF, Pal, MreB/C (Vollmer and Bertsche, 2008; Gillespie *et al*., 2010); however, only three genes encoding MrdA, FtsI (PbpB) and DacC were identified in *N. helminthoeca* (Fig. 5). In addition, except for an AmpG permease (NHE_RS03475) that can transport components of peptidoglycan into the cytoplasm, *N. helminthoeca* lacks all necessary enzymes required for the degradation and recycling of peptidoglycan, including lytic transglycosylases (LTs), AmpD, AnmK, LdcA, Mpl, YcjI/G, NagA/B/K/Z, PepD and MurQ (Gillespie *et al*., 2010). Furthermore, the T4SS usually encodes specialized LTs that hydrolyse and facilitate the local disruption of peptidoglycan, allowing for efficient transporter assembly across the entire cell envelope (Mushegian *et al*., 1996). For example, a specialized LT *virB1* homologue (*rvhB1*) was identified in *Rickettsia* spp. that encode pathways for biosynthesis and degradation of peptidoglycan; however, *virB1* homologue was not identified in *N. helminthoeca* and other members of the family Anaplasmataceae (Gillespie *et al*., 2010). Our previous electron microscopy showed that only two layers (outer and inner) of membranes and no thickening of the inner or outer leaflet of the outer membrane were present in *N. helminthoeca* (Rikihisa *et al*., 1991), suggesting that *N. helminthoeca* might not possess a peptidoglycan layer.

However, it is possible that *N. helminthoeca* can still produce precursors or components of peptidoglycan. As several peptidoglycan components are potent stimulants for innate immunity and antimicrobial responses in host immune defensive cells (Dziarski, 2003; Guan and Mariuzza, 2007; Sukhithasri *et al*., 2013), the presence of these components in *N. helminthoeca* could elicit antimicrobial and inflammatory activities in leucocytes and may account for the high acute mortality of SPD (Philip, 1955; Rikihisa *et al*., 1991) compared to less severe or chronic infections caused by other *Neorickettsia*,* Ehrlichia*, or *Anaplasma* spp. that lack peptidoglycan biosynthesis genes.

#### Lipoproteins and putative outer membrane proteins

Our previous study indicates that *E. chaffeensis* expresses mature lipoproteins on the bacterial surface, which induced delayed‐type hypersensitivity reaction in dogs (Huang *et al*., 2008). This study found *N. helminthoeca*, like other sequenced members of the family Anaplasmataceae, encodes all three lipoprotein‐processing enzymes (Lgt, LspA and Lnt; Table S10; Gupta and Wu, 1991; Paetzel *et al*., 2002). Computational analysis with LipoP 1.0 (http://www.cbs.dtu.dk/services/LipoP; Juncker *et al*., 2003) identified thirteen putative lipoproteins in *N. helminthoeca* (Table S10), which may also be involved in pathogenesis and immune response in infected canids as in *E. chaffeensis* (Huang *et al*., 2008). Homologues of several *N. helminthoeca* lipoproteins are also identified as lipoproteins in *N. risticii*, including OmpA, CBS domain protein and VirB6 family proteins (Table S1 and S10; Lin *et al*., 2009).

**Figure 6:**
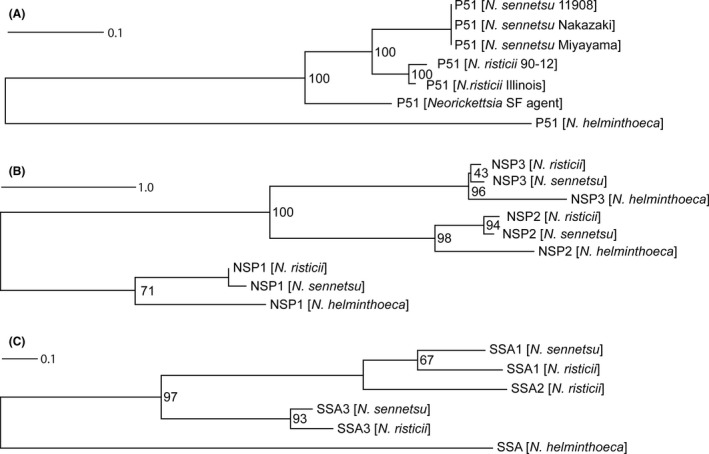
Phylogenetic tree of putative outer membrane proteins in *Neorickettsia* spp. The amino acid sequences of putative OMPs (P51, NSPs and SSAs) from *N. helminthoeca, N. risticii* and *N. sennetsu* were aligned with clustalw, the phylogenetic tree was built using raxml, and the tree was visualized with Dendroscope as described in the ‘Experimental procedures’. *N. helminthoeca* encodes P51, NSP1/2/3 and one copy of SSA (closest to SSA3), while *ssa2* gene of *N. sennetsu* is degenerated. For all three putative OMP groups (P51, NSPs, SSAs), *N. helminthoeca* OMPs forms a separate clade from those of *N. risticii* and *N. sennetsu*. GenBank Accession numbers: P51 proteins – *N. helminthoeca* Oregon, WP_051579521; *N. sennetsu* Miyayama, WP_011451642; *N. sennetsu* strain 11908, AAL79561; *N. sennetsu* Nakazaki, AAR23990; *N. risticii* Illinois, WP_015816118; *N. risticii* strain 90‐12, AAB46982; *Neorickettsia* sp. SF agent, AAR23988. NSP Proteins: *N. helminthoeca* Oregon – NSP1, WP_038560103; NSP2, WP_038560106; NSP3, WP_038560109; *N. sennetsu* Miyayama – NSP1, WP_011452245; NSP2, WP_011452246; NSP3, WP_011452248; *N. risticii* Illinois – NSP1, WP_015816683; NSP2, WP_015816684; NSP3, WP_015816686. SSA Proteins: *N. helminthoeca* Oregon – SSA, WP_038560160; *N. sennetsu* Miyayama – SSA1, WP_011452276; SSA3, WP_011452279; *N. risticii* Illinois – SSA1, WP_015816716; SSA2, WP_015816703; SSA3, WP_015816717.

Computational analysis using the pSort‐B algorithm predicted only four outer membrane proteins, two of which (BamD lipoprotein and beta‐barrel OMP BamA, also called Omp85/YaeT), are part of the beta‐barrel assembly machinery (BAM) and essential for the folding and insertion of outer membrane proteins of Gram‐negative bacteria (Surana *et al*., 2004; Table 4). Unlike *Ehrlichia* and *Anaplasma* spp. that encode a diverse members of the OMP‐1/P28/MSP2/P44 outer member superfamily proteins (Pfam01617), *Neorickettsia* spp. encode only one group of putative outer surface proteins that falls into this PFAM family (Dunning Hotopp *et al*., 2006). This group of proteins consists of three *N. helminthoeca* surface proteins (NSP1/2/3), which are approximately 30 kDa in mass and likely surface‐exposed based on their similarities to *Ehrlichia* P28/Omp‐1 (Ohashi *et al*., 1998a, 2001), *A. phagocytophilum* P44 (Zhi *et al*., 1998) and *N. risticii*/*N. sennetsu* NSPs (Gibson *et al*., 2010, 2011; Fig. 6B and Table 4).

In addition to NSP family OMPs, several studies have identified additional sets of potential surface proteins in other *Neorickettsia* spp., which include a 51‐kDa protein (P51) and *Neorickettsia* strain‐specific antigens (SSA; Biswas *et al*., 1998; Vemulapalli *et al*., 1998; Rikihisa *et al*., 2004; Lin *et al*., 2009; Gibson *et al*., 2010, 2011). P51 belongs to an orthologue cluster (cluster 409) that exists in all Rickettsiales (Dunning Hotopp *et al*., 2006), and is highly conserved among all sequenced *Neorickettsia* spp. including *N. helminthoeca* (NHE_RS00965) and the SF agent (Rikihisa *et al*., 2004; Fig. 6A). Previous studies have shown that P51 is the major antigenic protein recognized in horses with Potomac horse fever, and an immunofluorescence assay (IFA) using anti‐P51 antibody on non‐permeabilized *N. risticii* organisms showed a ring‐like labelling pattern surrounding the bacteria, indicating that P51 is a surface‐exposed antigen (Gibson and Rikihisa, 2008). P51 of *N. sennetsu* was demonstrated as a porin (Gibson *et al*., 2010). Phylogeny estimation (Fig. 6A), SignalP prediction (http://www.cbs.dtu.dk/services/SignalP/) and two‐dimensional structures (Fig. S2) suggests that similar to P51 of *N. sennetsu* and *N. risticii*,* N. helminthoeca* P51 is likely a β‐barrel protein localized to the outer membrane.

Strain‐specific antigens (SSAs), proteins of ~50 kDa with extensive intramolecular repeats, have been reported to be a protective antigen of *N. risticii* against homologous challenge (Biswas *et al*., 1998; Dutta *et al*., 1998). Unlike *N. risticii* or *N. sennetsu* that encodes two to three tandem genes of non‐identical SSAs, *N. helminthoeca* only encodes one SSA protein (NHE_RS03855, 35 kDa; Fig. 6C, Table 4 and S9). Phylogenetic analysis reveals that the SSA family proteins in *N. sennetsu* and *N. risticii* likely expanded following divergence from *N. helminthoeca*, but prior to the divergence of *N. risticii* and *N. sennetsu* (Fig. 6C). Sequence analysis also identified several intramolecular tandem repeats in *N. helminthoeca* P51 and SSA proteins (Table S9), suggesting that they might play important roles in pathogenesis and pathogen–host interactions (Citti and Wise, 1995; Smith *et al*., 1996).

#### Immunoreactivities of putative outer membrane proteins

Except for *Candidatus* ‘X. pacificiensis’ that maintains many genes involved in flagella assembly like hook, ring and rod (Kwan and Schmidt, 2013), all members of the family Anaplasmataceae lack LPS, capsule, flagella, or common pili (Dunning Hotopp *et al*., 2006). In agreement with our previous electron microscope images (Rikihisa *et al*., 1991), analysis of *N. helminthoeca* genome indicates that it did not produce a type 4 pili. Therefore, outer membrane proteins play critical roles in bacterium–host cell interactions and induce strong humoral immune responses (Rikihisa *et al*., 1992, 1994; Zhi *et al*., 1998; Rikihisa *et al*., 2004; Ohashi *et al*., 1998b; Gibson *et al*., 2011). Analysis of infection‐induced immune reactions to outer membrane proteins provide tools to determine prevalence of *N. helminthoeca* exposure/infection among various species of animals and provide a groundwork for developing novel rapid immunodiagnostic methods and protective vaccines for SPD.

**Figure 7:**
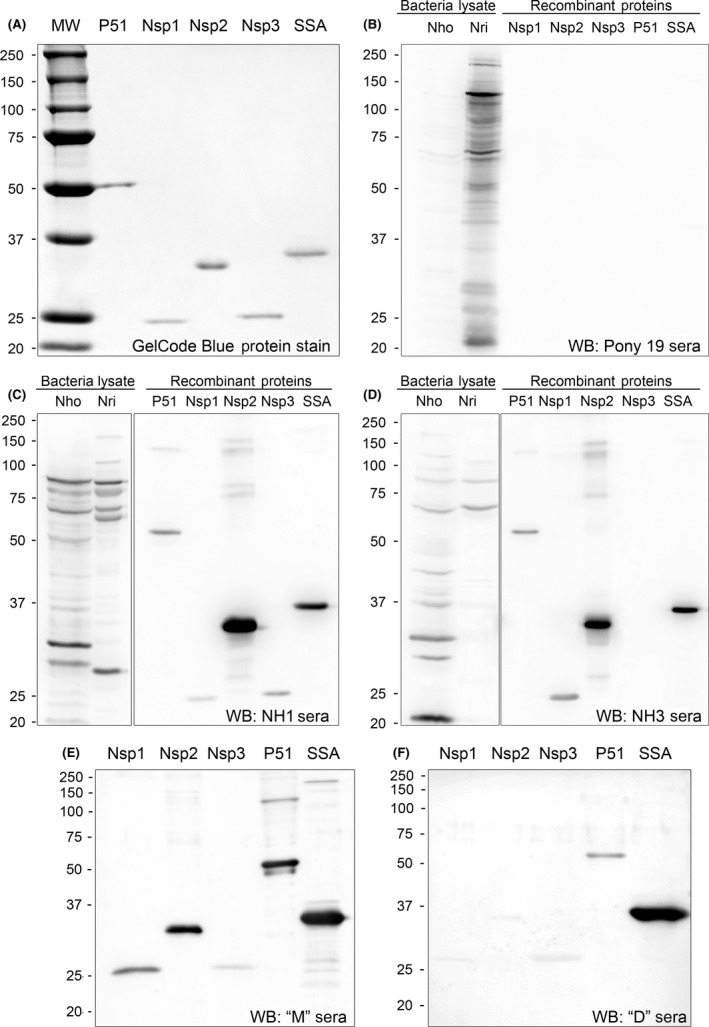
Expression and immunoreactivities of *N. helminthoeca* putative outer membrane proteins. P51, NSPs and SSA proteins were cloned into pET33(+) expression vector and recombinant proteins were purified from transformed *E. coli* BL21(DE3) strain. The size and purity of these recombinant proteins were verified by GelCode blue protein stain (A). *N. helminthoeca* (70% infected DH82 cells) and *N. risticii* (90%‐infected P388D1) from 2× T175 flasks were purified by sonication and filtration through 5‐μm filters. ~50 μg each of bacterial lysates from *N. risticii* (Nri) and *N. helminthoeca* (Nho) and ~20 μg of purified recombinant outer membrane proteins of *N. helminthoeca* were subjected to Western blot analysis and probed with (B) Pony 19 sera against *N. risticii* from experimentally infected pony (1/400 dilution), (C and D) NH1 and NH3 sera against *N. helminthoeca* from the experimentally infected dogs or (E and F) clinical dog sera from southern California that were positive for *N. helminthoeca* infection by PCR or IFA. Bands were visualized by ECL. The molecular size of the recombinant proteins are P51, 51.6 kDa; SSA, 33.7 kDa; NSP1, 27.7 kDa; NSP2, 32.2 kDa; NSP3, 23.7 kDa.

To elucidate immune reactions of SPD dog sera to P51, NSP1/2/3 and SSA, these proteins were cloned into the pET‐33b(+) expression vector, and recombinant proteins were purified from transformed *E. coli* (Fig. 7A). The immunoreactivities of these surface proteins were analysed using defined *N. helminthoeca* IFA‐positive dog sera (Rikihisa *et al*., 1991). Western blot analysis results showed that P51, NSP1/2/3 and SSA proteins were recognized by antisera from NH1 and NH3 dogs experimentally infected with *N. helminthoeca* by feeding trematodes‐parasitized fish and seroconverted (IFA titres of 1:640 and 1:1280, respectively, using *N. helminthoeca*‐infected DH82 cells as the antigen; Rikihisa *et al*., 1991), with NSP2 and SSA as the strongest sero‐reactive antigens (Fig. 7C,D). In addition, *N. helminthoeca*‐positive dog sera from naturally infected dogs from southern California recognized P51 and SSA and weakly against NPS3, whereas NSP1 and NSP2 were only detected by ‘M’ sera (Fig. 7E,F). As a control, antisera from the horse experimentally infected with *N. risticii* did not react with any of these membrane proteins from *N. helminthoeca* (Fig. 7B). These data indicate that *N. helminthoeca* OMPs including P51, SSA and NSPs can be recognized by the immune system of *N. helminthoeca‐*infected dogs.

Our previous study showed that sera from *N. helminthoeca*‐infected dogs, *N. sennetsu*‐infected horse, *N. risticii*‐infected horses, or *E. canis*‐infected dogs cross‐reacted with other species but with at least 16‐fold lower than those for homologous antigens by immunofluorescence assay (Rikihisa, 1991b; Rikihisa *et al*., 1991). This study also showed that approximately 78–80 kDa and 64 kDa proteins were the major antigens shared by *N. helminthoeca*,* N. risticii*,* N. sennetsu* and *E. canis* (Rikihisa, 1991b; Fig. 7B–D). These cross‐reactive antigens were likely more conserved heat‐shock proteins or molecular chaperones, and their molecular weights were different from predicted outer membrane proteins of *N. helminthoeca* analysed in the current study (from 23 to 51 kDa). Therefore, in current Western blotting with the dilution of sera at 1:400, horse sera against *N. risticii* recognized none of *N. helminthoeca* OMPs (Fig. 7B), whereas dog sera against *N. helminthoeca* only detected proteins at ~64 and 80‐kD from *N. risticii* (Fig. 7C,D), suggesting that these recombinant OMPs could be used for specific diagnosis of *N. helminthoeca*‐infected dogs.

## Conclusion and discussion

Despite of expansion of DNA sequences of *Neorickettsia* spp. in various trematode species worldwide, biology and natural history have been best studied in *N. helminthoeca*, the type species of the genus *Neorickettsia*. In this study, we determined and analysed the complete genome sequence of *N. helminthoeca*, providing a valuable resource necessary for understanding the metabolism of *N. helminthoeca* and its digenean host associations, the evolution and phylogeny among *Neorickettsia* spp., potential virulence factors of *N. helminthoeca*, pathogenic mechanisms of SPD and environmental spreading of *N. helminthoeca* and trematodes infection in nature. Comparative genomics data of three *Neorickettsia* spp. of known biological significance is expected to help elucidating biology of other *Neorickettsia* spp. in the environment.

As SPD progression is rapid, and the case fatality rate is quite high, prevention and early diagnosis of SPD are critical. The serological assay based on defined outer membrane protein antigens is simple, consistent, specific, objective and convenient, thus helps generating epidemiological information on *N. helminthoeca* exposure among various wild and domestic animals to raise awareness of SPD. Similar to bats that are the definitive hosts of *Acanthatrium oregonense* trematodes, the vector of *N. risticii* transmission (Gibson *et al*., 2005; Gibson and Rikihisa, 2008), the definitive hosts of *N. helminthoeca*‐infected trematodes in nature are likely asymptomatic, but have antibodies against *N. helminthoeca*.

Furthermore, these recombinant proteins can be applied to develop a simple and rapid serodiagnostic test for SPD in dogs in the future. The limitation of the assay is, as in any other serologic assays, false negative results at early stages of infection and in immunosuppressed dogs. Future steps necessary for the test to become applicable for clinical diagnosis are to determine sensitivity and specificity of the test using a larger number of well‐defined canine specimens from broader geographic regions. For this and understanding the pathogenesis and canine immune responses in SPD, culture isolation of additional *N. helminthoeca* strains is desirable. Further characterization of the antigenic surface proteins of *N. helminthoeca* could provide valuable candidates for the development of rapid, sensitive and specific serodiagnostic approaches or preventive vaccines for SPD.

## Experimental procedures

### Organisms culture, bacteria purification and DNA preparation

*Neorickettsia helminthoeca* Oregon strain, which was previously isolated from dog NH1 fed with fluke *N. salmincola*‐infested salmon kidneys (Rikihisa *et al*., 1991), was cultured in DH82 cells from the frozen cell stock in Dulbecco's minimal essential medium supplemented with 10% fetal bovine serum and 2 mM l‐glutamine. Cultures were incubated at 37°C under 5% CO_2_ in a humidified atmosphere. To purify host cell‐free bacteria for genome sequencing, infected cells (> 95% infection) were harvested and Dounce homogenized in SPK buffer (0.2 M sucrose and 0.05 M potassium phosphate, pH 7.4). Lysed cells were centrifuged at 500× g and 700× g to remove unbroken cells and nuclei, filtered through 5.0‐ and 2.7‐μm syringe filters and centrifuged at 10 000× g to pellet host cell‐free bacteria. Genomic DNA was purified using a Genomic‐tip 20/G (QIAGEN, Valencia, CA, USA) according to manufacturer's instructions, and host DNA contamination was verified to be < 0.1% by PCR using specific primers targeting *N. helminthoeca* 16S rRNA gene and canine G3PDH DNA.

### Sequencing and annotation

Indexed Illumina mate pair libraries were prepared following the mate pair library v2 sample preparation guide (Illumina, San Diego, CA, USA), with two modifications. First, the shearing was performed with the Covaris E210 (Covaris, Wobad, MA, USA). The DNA was purified using enzymatic reactions and the size selection of the library was performed with AMPure XT beads (Beckman Coulter Genomics, Danvers, MA, USA).

Illumina non‐Truseq paired‐end genomic DNA libraries were constructed using the KAPA library preparation kit (Kapa Biosystems, Woburn, MA, USA). DNA was fragmented with the Covaris E210. Then libraries were prepared using a modified version of manufacturer's protocol. The DNA was purified using enzymatic reactions and the size selection of the library was performed with AMPure XT beads (Beckman Coulter Genomics, Danvers, MA, USA). For indexed samples, the PCR amplification step was performed with primers containing a six nucleotide index sequence.

Concentration and fragment size of libraries were determined using the DNA High Sensitivity Assay on the LabChip GX (Perkin‐Elmer, Waltham, MA, USA) and qPCR using the KAPA Library Quantification Kit (Complete, Universal; Kapa Biosystems, Woburn, MA, USA). The mate pair library was sequenced on an Illumina HiSeq 2500 (Illumina, San Diego, CA, USA) while the paired‐end library was sequenced on an Illumina MiSeq (Illumina, San Diego, CA, USA).

DNA samples for PacBio sequencing were sheared to 8 kbp using the Covaris gTube (Woburn, MA, USA). Sequencing libraries were constructed and prepared for sequencing using the DNA Template Prep Kit 2.0 (3–10 kbp) and the DNA/Polymerase Binding Kit 2.0 (Pacific Biosciences, Menlo Park, CA, USA). Libraries were loaded onto v2 SMRT Cells and sequenced with the DNA Sequencing Kit 2.0 (Pacific Biosciences).

Five assemblies were generated with various combinations of the data and assembly algorithms: (i) celera assembler v7.0 of only PacBio data, (ii) celera assembler v7.0 of PacBio data with correction using Illumina paired‐end data, (iii) HGAP assembly of only PacBio data, (iv) MaSuRCA 1.9.2 assembly of Illumina paired‐end data subsampled to 50× coverage, and (v) MaSuRCA 1.9.2 assembly of Illumina paired‐end data subsampled to 80× coverage. The first assembly was the optimal assembly, namely the one generated with celera assembler v7.0 with only the PacBio data. The data set was subsampled to ~22× coverage of the longest reads using an 8 kbp minimum read length cut‐off, with the remainder of the reads used for the error correction step. The resulting single‐contig assembly totalled ~89.4 Kbp with 41.68% GC‐content. The genome was trimmed to remove overlapping sequences, oriented, circularized and rotated to the predicted origin of replication. Annotation for this finalized genome assembly was generated using the IGS prokaryotic annotation pipeline (Galens *et al*., 2011) and deposited in GenBank (accession number NZ_CP007481.1).

### Bioinformatic analysis

The 16S rRNA, NSP, P51 and SSA proteins were aligned with their *Neorickettsia* orthologues using clustalw (Thompson *et al*., 1994) as implemented in bioedit 7.2.5 (Hall, 1999) resulting in 1522 nt, 326 aa, 516 aa and 578 aa alignments respectively. A phylogenetic tree was inferred from the 16S rRNA alignment using raxml v.7.3.0 (Stamatakis *et al*., 2005) with the GTRGAMMA model, specifically ‘RAxMLHPC ‐f a ‐m GTRGAMMA ‐p 12345 ‐x 12345 ‐N autoMRE ‐n T20’. The MRE‐based bootstopping criterion was not met, resulting in the use of 1000 bootstraps. For the protein alignments, the best‐fit model of amino acid substitution was determined for each alignment separately with prottest 3.2 (Darriba *et al*., 2011), with all 15 models of protein evolution tested in addition to the +G parameter. WAG+G was determined to be the best model for NSP and SSA while JTT was determined to be the best model for P51. Phylogenetic trees were inferred from the NSP and SSA alignments using raxml v.7.3.0 (Stamatakis *et al*., 2005) with the best model, specifically ‘RAxMLHPC ‐f a ‐m PROTGAMMAWAG ‐p 12345 ‐x 12345 ‐N autoMRE ‐n T20’. The MRE‐based bootstopping criterion was met at 350 replicates for NSP and SSA. Phylogenetic trees were inferred from the P51 alignment using raxml v.7.3.0 (Stamatakis *et al*., 2005) with the best model, specifically ‘RAxMLHPC ‐f a ‐m PROTCATJTT ‐p 12345 ‐x 12345 ‐N autoMRE ‐n T20’. The MRE‐based bootstopping criterion was met at 50 replicates for P51. All trees and bootstrap values were visualized in dendroscope v3.5.7 (http://dendroscope.org, Tübingen University, Tübingen, Germany).

The GC skew was calculated as (C‐G)/(C+G) in windows of 500 bp with step size of 250 bp along the chromosome. Synteny plots between *Neorickettsia* spp. were generated using MUMmer 3 program with default parameters (Delcher *et al*., 2002). Protein orthologue clusters among *Neorickettsia* spp., and *N. helminthoeca*‐specific genes compared to other related organisms were determined using reciprocal blastp with cut‐off scores of *E *<* *10^−10^.

Metabolic pathways and transporters were compared across genomes using (i) the orthologue clusters generated with reciprocal blastp, (ii) Genome Properties (Haft *et al*., 2005), (iii) TransportDB (Ren *et al*., 2007), (iv) Kyoto Encyclopedia of Genes and Genomes (KEGG, http://www.kegg.jp), and (v) Biocyc (Krieger *et al*., 2004). Signal peptides and membrane proteins were predicted using the pSort‐B algorithm (http://psort.org/psortb/;Yu *et al*., 2010), and lipoproteins were predicted by lipop 1.0 (http://www.cbs.dtu.dk/services/LipoP; Juncker *et al*., 2003).

### Cloning, expression and Western blot analysis of putative *N. helminthoeca* outer membrane proteins

Full‐length *p51*,* nsp*1/2/3 and *ssa* genes without the signal peptide sequence were PCR amplified from *N. helminthoeca* genomic DNA, using specific primers (Table S11) and cloned into the pET‐33b(+) vector (Novagen, Billerica, MA, USA). The plasmids were amplified by transformation into *Escherichia coli* PX5α cells (Protein Express, Cincinnati, OH, USA), and the inserts were confirmed by sequencing. The plasmids were transformed into *E. coli* BL21 (DE3; Protein Express), and the expression of recombinant proteins was induced with 1 mM isopropyl β‐d‐thiogalactopyranoside. *E. coli* was sonicated for a total of 5 min (15 s pulse with 45 s interval) on ice, and the pellet containing recombinant protein was washed with 1% Triton X‐100 in sodium phosphate buffer (SPB: 50 mM sodium phosphate, pH 8.0, 0.3 M NaCl). Recombinant proteins were denatured and solubilized with 6 M urea in SPB (for P51, SSA and NSP2/3), or 6M Guanidine HCl in SPB (for NSP1) at 4°C for 1 h. Proteins were purified on a HisPur Cobalt Affinity resin (Pierce, Rockford, IL, USA) and dialysed using Buffer A (50 mM KCl, 100 mM NaCl, 50 mM Tris‐HCl, pH 8.0) containing decreasing concentrations of urea (3 M, 1 M, then 0 M). Protein concentrations were determined by BCA assay (Pierce).

Bacterial lysates of purified *N. risticii* or *N. helminthoeca*, and recombinant NSP1/2/3, SSA and P51 were subjected to SDS‐polyacrylamide gel electrophoresis (SDS‐PAGE) and Western blot analysis as described previously (Lin *et al*., 2002). Gels were stained using GelCode Blue (Pierce), and the immunoreactivities of these recombinant proteins were determined by Western blot analysis using SPD dog sera against *N. helminthoeca* or horse anti‐*N. risticii* serum as a negative control at 1:400 dilutions. Defined SPD dog sera against *N. helminthoeca* were obtained from dogs orally fed by fluke *N. salmincola*‐infested salmon kidneys infected with *N. helminthoeca*, and sera collected at day 13 and 15 postexposure with IFA titres at 1:640 (NH1) and 1:1280 (NH3) respectively (Rikihisa *et al*., 1991). Clinical dog sera tested positive for *N. helminthoeca* infection were received from southern California (‘M’ sera – IFA titre 1:80, from Dana Point, CA in 2012; ‘D’ sera – PCR‐positive for *N. helminthoeca 16S rRNA* gene, from Aliso Viejo, CA in 2010). Horse anti‐*N. risticii* serum (Pony 19) was collected from a pony inoculated intravenously with *N. risticii*‐infected U‐937 cells (IFA titre 1:640; Rikihisa *et al*., 1988). Reacting bands were detected with Horseradish peroxidase (HRP)‐conjugated goat anti‐dog (KPL Gaithersburg, MD) or anti‐horse (Jackson Immuno Research, West Grove, PA) secondary antibodies and visualized with enhanced chemiluminescence (ECL) by incubating the membranes with LumiGLO™ chemiluminescent reagent (Pierce). Images were captured using an LAS3000 image documentation system (FUJIFILM Medical Systems USA, Stamford, CT, USA).

### GenBank Accession numbers and abbreviations of bacteria

*N. helminthoeca* Oregon (NHO), NZ_CP007481.1 (this study); *N. risticii* Illinois (NRI), NC_013009.1; *N. sennetsu* Miyayama (NSE), NC_007798.1; *A. phagocytophilum* HZ (APH), NC_007797.1; *A. marginale* Florida (AMA), NC_012026.1; *E. chaffeensis* Arkansas (ECH), NC_007799.1; *E. canis* Jake (ECA), NC_007354.1; *E. ruminantium* Welgevonden (ERU), NC_005295.2; *E. muris* AS145 (EMU), NC_023063.1; *Ehrlichia* sp. HF (EHF), NZ_CP007474.1; *Wolbachia pipientis* (*w*Mel, *Wolbachia* endosymbiont of *Drosophila melanoga*), NC_002978.6; *Wolbachia* endosymbiont of *Brugia malayi* (*w*Bm), NC_006833.1; *Neorickettsia* endobacterium of *Fasciola hepatica* (NFh), NZ_LNGI00000000; *Candidatus* Xenolissoclinum pacificiensis L6, AXCJ00000000.

## Conflict of interest

No potential conflicts of interest were disclosed.

## Supporting information


**Fig. S1.** Synteny plots between *Neorickettsia* spp.

**Fig. S2.** Secondary Structure of *N. helminthoeca* P51 Protein.

**Fig. S3.** Phylogenetic tree of VirB2 proteins in the family Anaplasmataceae and α‐proteobacteria.

**Fig. S4.** One‐component regulatory systems of *N. helminthoeca*.

**Fig. S5.** Phylogenetic analysis of AnkA or Ank200 homologous proteins in the family Anaplasmataceae.

**Table S1.** Ortholog clusters conserved among *N. helminthoeca*,* N. risticii* and *N. sennetsu* based on three‐way comparison analysis

**Table S2.** *N. helminthoeca*‐specific proteins compared to *N. sennetsu* and *N. risticii*

**Table S3.** *N. risticii*‐specific proteins compared to *N. helminthoeca* and *N. sennetsu*

**Table S4.** *N. sennetsu*‐specific proteins compared to *N. helminthoeca* and *N. risticii*

**Table S5.** Amino acid and cofactor biosynthesis in Family Anaplasmataceae

**Table S6.** Potential pathogenic genes in *Neorickettsia* species

**Table S7.** Putative Transporters of *N. helminthoeca*

**Table S8.** Genes involved in DNA repair and homologous recombination

**Table S9.** Proteins with tandem repeats in *N. helminthoeca*

**Table S10.** Lipoprotein‐processing enzymes and putative lipoproteins in *N. helminthoeca*

**Table S11.** Oligonucleotide primers used for cloning *N. helminthoeca* outer membrane proteins
