# Supplementary figures and images for: Analysis of complete genome sequence and major surface antigens of Neorickettsia helminthoeca, causative agent of salmon poisoning disease
Source: Microb Biotechnol. 2017 Jun 6;10(4):933–57. doi: 10.1111/1751-7915.12731 (PMC5481527; doi:10.1111/1751-7915.12731)

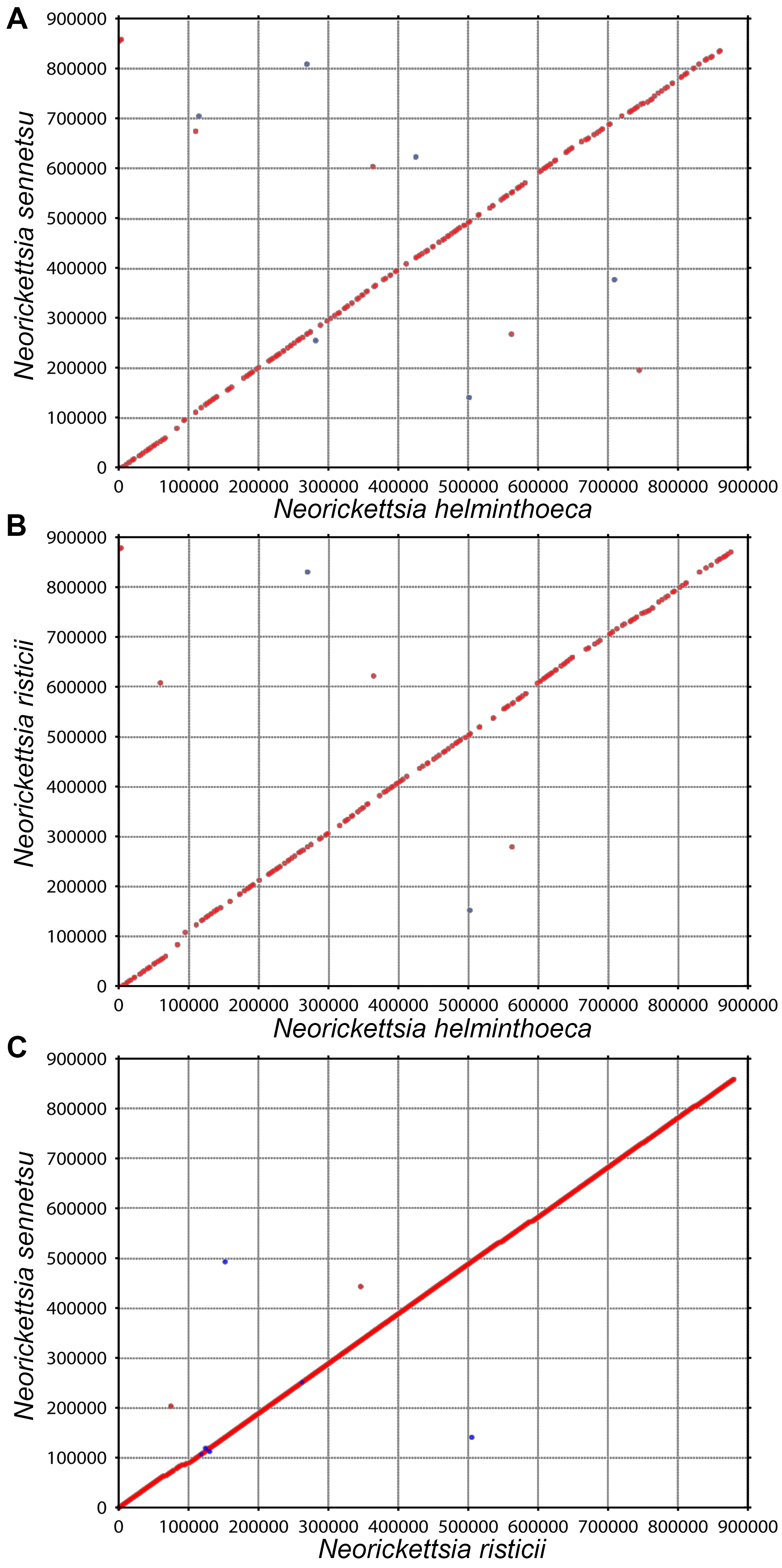

Supplement: Supplementary file 1 — Fig. S1. Synteny plots between Neorickettsia spp. [file MBT2-10-933-s001.tif]

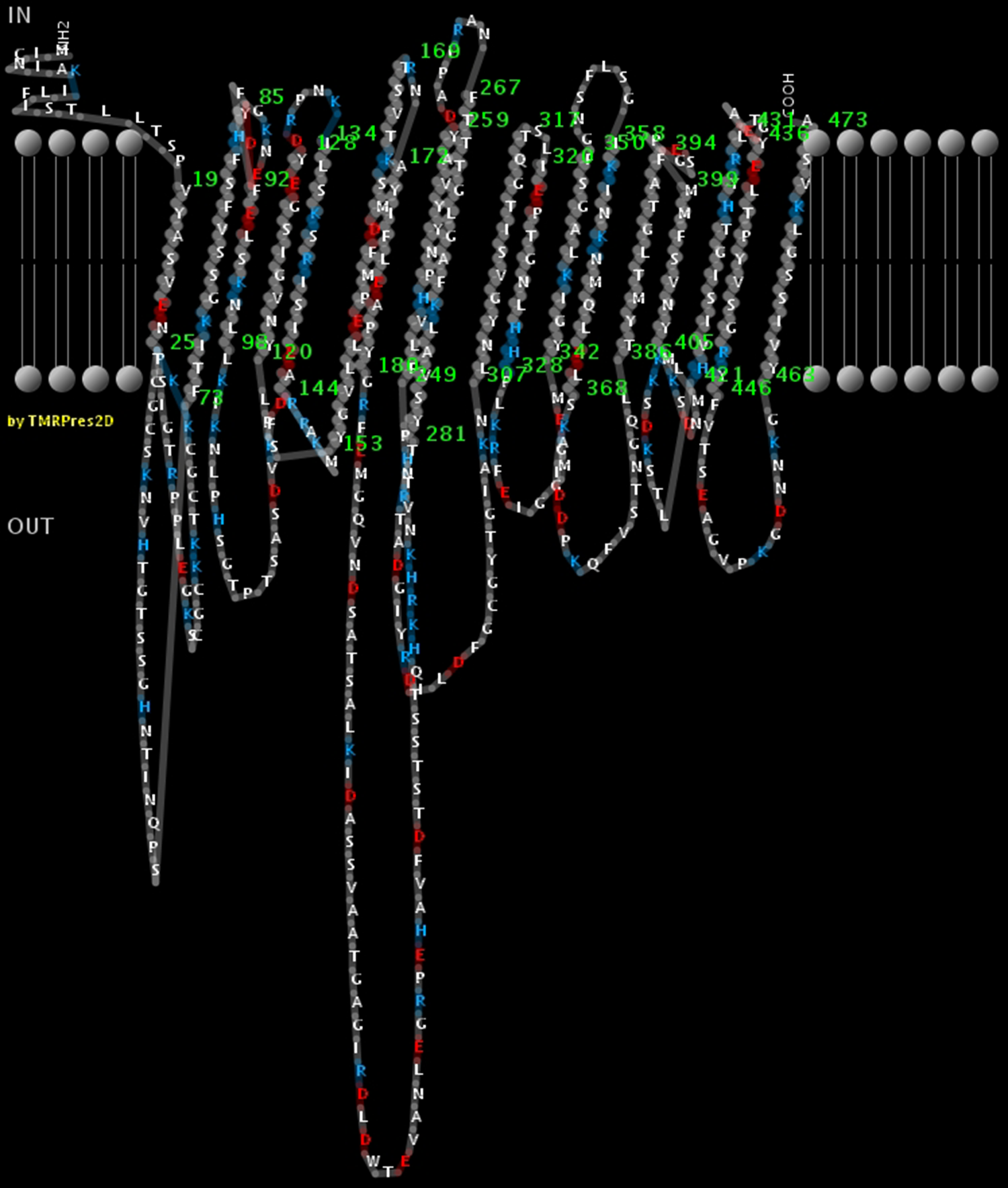

Supplement: Supplementary file 2 — Fig. S2. Secondary Structure of N. helminthoeca P51 Protein. [file MBT2-10-933-s002.tif]

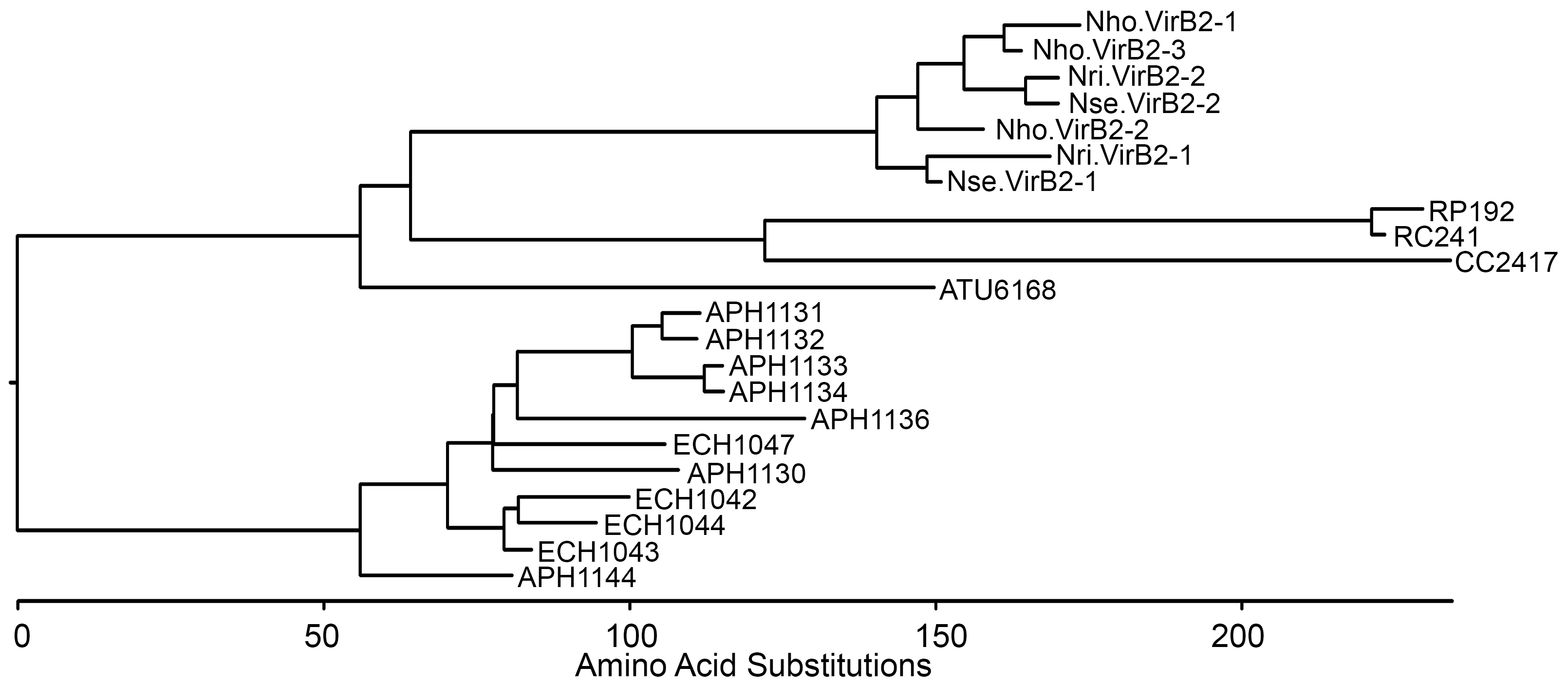

Supplement: Supplementary file 3 — Fig. S3. Phylogenetic tree of VirB2 proteins in the family Anaplasmataceae and α‐proteobacteria. [file MBT2-10-933-s003.tif]

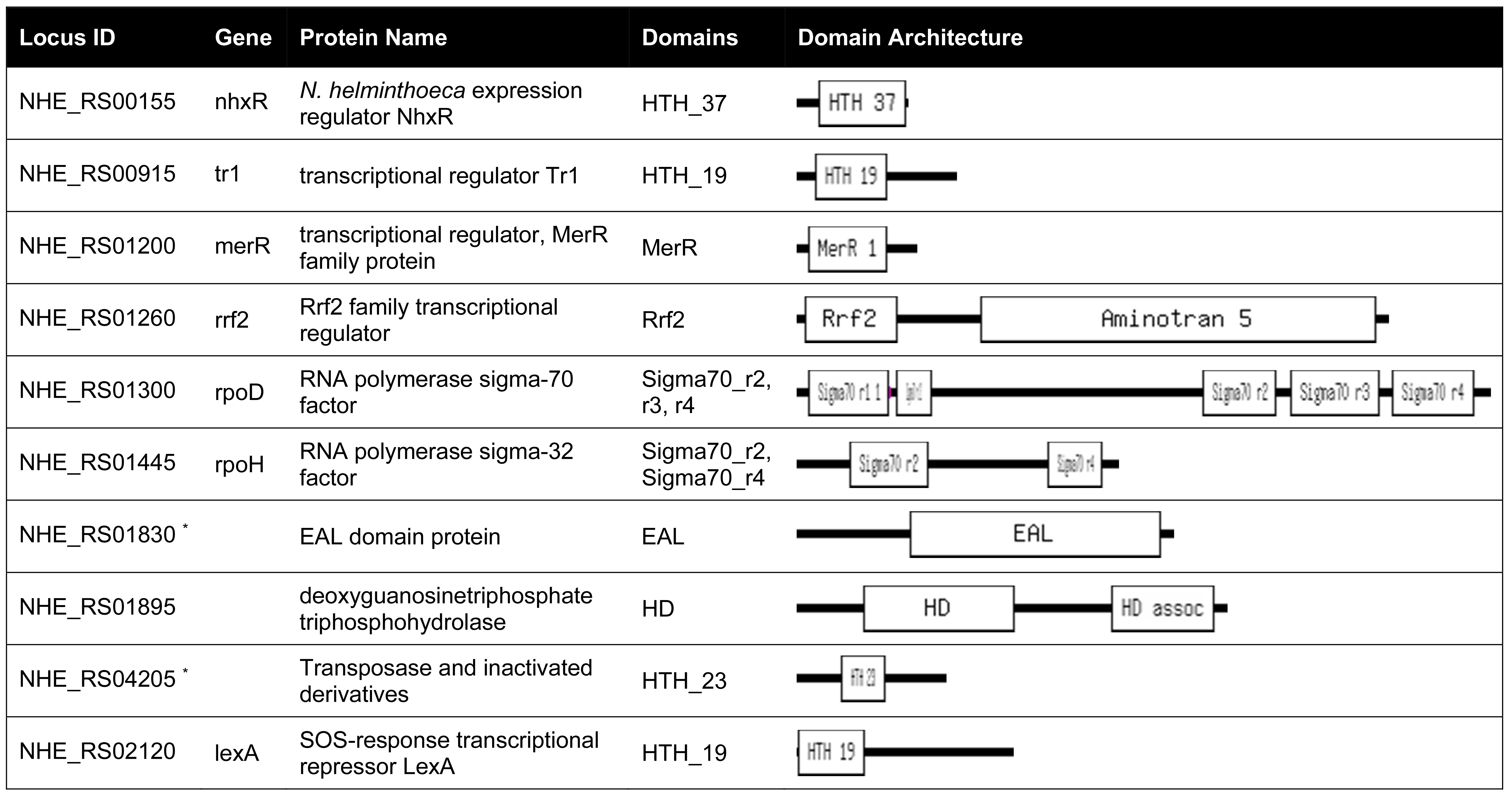

Supplement: Supplementary file 4 — Fig. S4. One‐component regulatory systems of N. helminthoeca. [file MBT2-10-933-s004.tif]

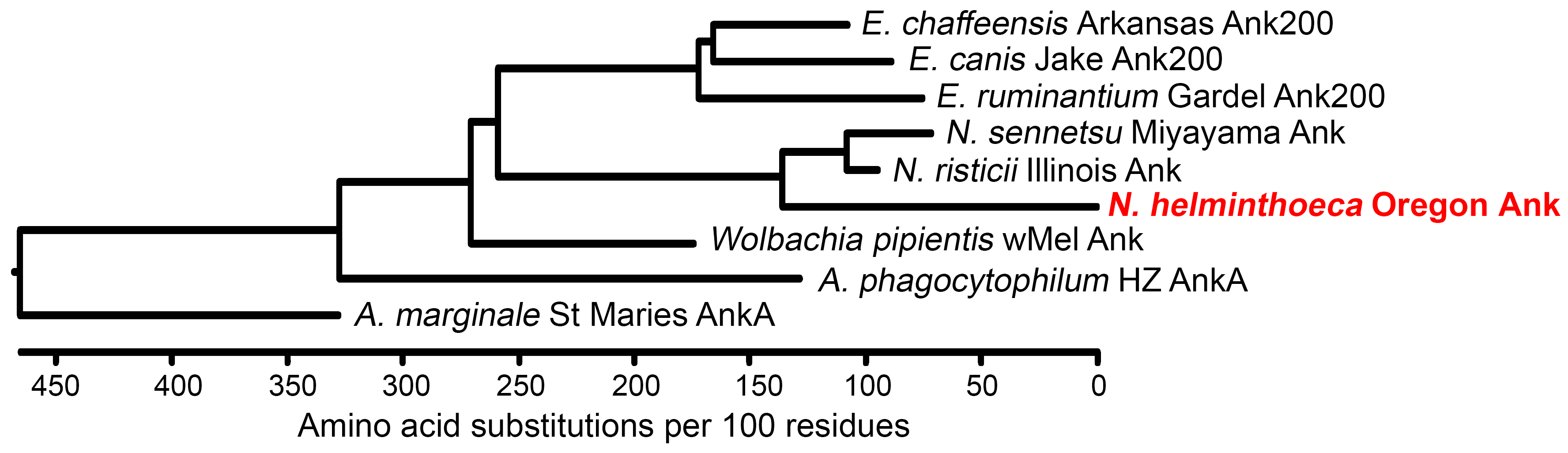

Supplement: Supplementary file 5 — Fig. S5. Phylogenetic analysis of AnkA or Ank200 homologous proteins in the family Anaplasmataceae. [file MBT2-10-933-s005.tif]
