# Supplementary material for: Analysis of complete genome sequence and major surface antigens of Neorickettsia helminthoeca, causative agent of salmon poisoning disease
Source: Microb Biotechnol. 2017 Jun 6;10(4):933–57. doi: 10.1111/1751-7915.12731 (PMC5481527; doi:10.1111/1751-7915.12731)
Supplement: Supplementary file 6 — Table S1. Ortholog clusters conserved among N. helminthoeca, N. risticii and N. sennetsu based on three‐way comparison analysis [file MBT2-10-933-s006.pdf]

Supplementary Table 1. Ortholog clusters conserved among *Neorickettsia helminthoeca*, *N. risticii*, and *N. sennetsu* based on three-way comparison analysis [1]

Ortholog Clusters	Protein Name	Role Category
**Amino acid biosynthesis**		
NSE_RS00705,NRI_RS00745,NHE_RS00695	putative 3-phosphoshikimate 1-carboxyvinyltransferase	Amino acid biosynthesis | Aromatic amino acid family
NSE_RS03830,NRI_RS03910,NHE_RS04025	AraM domain protein	Amino acid biosynthesis | Aromatic amino acid family
NSE_RS01045,NRI_RS01085,NHE_RS01045	aspartate-semialdehyde dehydrogenase	Amino acid biosynthesis | Aspartate family
NSE_RS03085,NRI_RS03170,NHE_RS03235	aspartate aminotransferase	Amino acid biosynthesis | Aspartate family
NSE_RS03785,NRI_RS03870,NHE_RS03980	dihydrodipicolinate synthase	Amino acid biosynthesis | Aspartate family
NSE_RS01455,NRI_RS01505,NHE_RS01490	glutamine synthetase, type I	Amino acid biosynthesis | Glutamate family
NSE_RS02825,NRI_RS02915,NHE_RS02945	glutamine synthetase domain protein	Amino acid biosynthesis | Glutamate family
NSE_RS02670,NRI_RS02760,NHE_RS02780	bifunctional glutamate synthase subunit beta/2-polyprenylphenol hydroxylase (GS/PH)	Amino acid biosynthesis | Glutamate family
NSE_RS00865,NRI_RS00905,NHE_RS00860	serine hydroxymethyltransferase	Amino acid biosynthesis | Serine family
**Biosynthesis of cofactors and prosthetic groups**		
NSE_RS02480,NRI_RS02540,NHE_RS02585	biotin synthase	Biosynthesis of cofactors, prosthetic groups, and carriers | Biotin
NSE_RS02485,NRI_RS02545,NHE_RS02590, NHE_RS03545,NRI_RS03445	8-amino-7-oxononanoate synthase	Biosynthesis of cofactors, prosthetic groups, and carriers | Biotin
NSE_RS02495,NRI_RS02555,NHE_RS02600	biotin biosynthesis protein BioC	Biosynthesis of cofactors, prosthetic groups, and carriers | Biotin
NSE_RS02505,NRI_RS02565,NHE_RS02610, NHE_RS03625,NRI_RS03545	adenosylmethionine-8-amino-7-oxononanoate aminotransferase	Biosynthesis of cofactors, prosthetic groups, and carriers | Biotin
NSE_RS03365,NRI_RS03445,NHE_RS03545, NRI_RS02545,NHE_RS02590	5-aminolevulinic acid synthase	Biosynthesis of cofactors, prosthetic groups, and carriers | Biotin
NSE_RS03465,NRI_RS03545,NHE_RS03625, NRI_RS02565,NHE_RS02610	acetylornithine aminotransferase	Biosynthesis of cofactors, prosthetic groups, and carriers | Biotin
NSE_RS01965,NRI_RS02005,NHE_RS02010	dihydropteroate synthase	Biosynthesis of cofactors, prosthetic groups, and carriers | Folic acid
NSE_RS02030,NRI_RS02075,NHE_RS02085	putative dihydroneopterin aldolase	Biosynthesis of cofactors, prosthetic groups, and carriers | Folic acid
NSE_RS02405,NRI_RS02460,NHE_RS02505	GTP cyclohydrolase I	Biosynthesis of cofactors, prosthetic groups, and carriers | Folic acid
NSE_RS02905,NRI_RS02995,NHE_RS03030	folylpolyglutamate synthase	Biosynthesis of cofactors, prosthetic groups, and carriers | Folic acid
NSE_RS03340,NRI_RS03420,NHE_RS03505	FolD bifunctional protein	Biosynthesis of cofactors, prosthetic groups, and carriers | Folic acid
NSE_RS00730,NRI_RS00765,NHE_RS00720	glutathione synthetase	Biosynthesis of cofactors, prosthetic groups, and carriers | Glutathione and analogs
NSE_RS01275,NRI_RS01320,NHE_RS01280	glutamate--cysteine ligase	Biosynthesis of cofactors, prosthetic groups, and carriers | Glutathione and analogs
NSE_RS01560,NRI_RS01610,NHE_RS01610	putative porphobilinogen deaminase	Biosynthesis of cofactors, prosthetic groups, and carriers | Heme, porphyrin, and cobalamin
NSE_RS01595,NRI_RS01645,NHE_RS01645	porphobilinogen synthase	Biosynthesis of cofactors, prosthetic groups, and carriers | Heme, porphyrin, and cobalamin

[1] Ortholog clusters were constructed using reciprocal BLASTP algorithm with E-value < $1e^{-10}$, and grouped by functional role categories. The protein name and role category of the designated ortholog cluster are based on those from *N. helminthoeca* genome.

NSE_RS01830,NRI_RS01870,NHE_RS01875	coproporphyrinogen III oxidase, aerobic	Biosynthesis of cofactors, prosthetic groups, and carriers | Heme, porphyrin, and cobalamin
NSE_RS02530,NRI_RS02590,NHE_RS02635	protoheme IX farnesyltransferase	Biosynthesis of cofactors, prosthetic groups, and carriers | Heme, porphyrin, and cobalamin
NSE_RS03200,NRI_RS03285,NHE_RS03360	ferrochelatase	Biosynthesis of cofactors, prosthetic groups, and carriers | Heme, porphyrin, and cobalamin
NSE_RS03950,NRI_RS04030,NHE_RS00005	uroporphyrinogen decarboxylase	Biosynthesis of cofactors, prosthetic groups, and carriers | Heme, porphyrin, and cobalamin
NSE_RS01340,NRI_RS01390,NHE_RS01350	lipoic acid synthetase	Biosynthesis of cofactors, prosthetic groups, and carriers | Lipoate
NSE_RS01405,NRI_RS01455,NHE_RS01435	Coq7 family protein	Biosynthesis of cofactors, prosthetic groups, and carriers | Menaquinone and ubiquinone
NSE_RS01555,NRI_RS01605,NHE_RS01605	ubiquinone biosynthesis hydroxylase, UbiH/UbiF/VisC/COQ6 family	Biosynthesis of cofactors, prosthetic groups, and carriers | Menaquinone and ubiquinone
NSE_RS02555,NRI_RS02615,NHE_RS02665	3-demethylubiquinone-9 3-methyltransferase	Biosynthesis of cofactors, prosthetic groups, and carriers | Menaquinone and ubiquinone
NSE_RS02585,NRI_RS02655,NHE_RS02705	putative ubiquinone biosynthesis protein	Biosynthesis of cofactors, prosthetic groups, and carriers | Menaquinone and ubiquinone
NSE_RS03275,NRI_RS03350,NHE_RS03435	4-hydroxybenzoate octaprenyltransferase	Biosynthesis of cofactors, prosthetic groups, and carriers | Menaquinone and ubiquinone
NSE_RS03150,NRI_RS03235,NHE_RS03305	molybdopterin biosynthesis protein MoeB	Biosynthesis of cofactors, prosthetic groups, and carriers | Molybdopterin
NSE_RS00525,NRI_RS00570,NHE_RS00520	2C-methyl-D-erythritol 2,4-cyclodiphosphate synthase	Biosynthesis of cofactors, prosthetic groups, and carriers | Other
NSE_RS00695,NRI_RS00735,NHE_RS00685	putative 2-C-methyl-D-erythritol 4-phosphate cytidylyltransferase	Biosynthesis of cofactors, prosthetic groups, and carriers | Other
NSE_RS00950,NRI_RS00990,NHE_RS00950, NRI_RS02905,NHE_RS02935	polyprenyl synthetase family protein	Biosynthesis of cofactors, prosthetic groups, and carriers | Other
NSE_RS01240,NRI_RS01285,NHE_RS01245	iron-sulfur cluster assembly accessory protein	Biosynthesis of cofactors, prosthetic groups, and carriers | Other
NSE_RS01245,NRI_RS01290,NHE_RS01250	FeS cluster assembly scaffold IscU	Biosynthesis of cofactors, prosthetic groups, and carriers | Other
NSE_RS01250,NRI_RS01295,NHE_RS01255	cysteine desulfurase	Biosynthesis of cofactors, prosthetic groups, and carriers | Other
NSE_RS01255,NRI_RS01300,NHE_RS01260	rrf2 family transcriptional regulator with aminotransferase, class V family protein	Biosynthesis of cofactors, prosthetic groups, and carriers | Other
NSE_RS01770,NRI_RS01810,NHE_RS01815	4-hydroxy-3-methylbut-2-enyl diphosphate reductase	Biosynthesis of cofactors, prosthetic groups, and carriers | Other
NSE_RS01790,NRI_RS01830,NHE_RS01835	1-deoxy-D-xylulose 5-phosphate reductoisomerase	Biosynthesis of cofactors, prosthetic groups, and carriers | Other
NSE_RS01845,NRI_RS01885,NHE_RS01890	putative iron-sulfur cluster assembly accessory protein	Biosynthesis of cofactors, prosthetic groups, and carriers | Other
NSE_RS02815,NRI_RS02905,NHE_RS02935, NRI_RS00990,NHE_RS00950	putative geranyltranstransferase	Biosynthesis of cofactors, prosthetic groups, and carriers | Other
NSE_RS02925,NRI_RS03015,NHE_RS03050	putative 4-diphosphocytidyl-2C-methyl-D-erythritol kinase	Biosynthesis of cofactors, prosthetic groups, and carriers | Other
NSE_RS03245,NRI_RS03325,NHE_RS03405	1-hydroxy-2-methyl-2-(E)-butenyl 4-diphosphate synthase	Biosynthesis of cofactors, prosthetic groups, and carriers | Other
NSE_RS01280,NRI_RS01325,NHE_RS01285	dephospho-CoA kinase	Biosynthesis of cofactors, prosthetic groups, and carriers | Pantothenate and coenzyme A
NSE_RS03960,NRI_RS04040,NHE_RS00015	pantetheine-phosphate adenylyltransferase	Biosynthesis of cofactors, prosthetic groups, and carriers | Pantothenate and coenzyme A
NSE_RS00395,NRI_RS00400,NHE_RS00400	NAD+ synthetase	Biosynthesis of cofactors, prosthetic groups, and carriers | Pyridine nucleotides
NSE_RS00470,NRI_RS00515,NHE_RS00465	nicotinate-nucleotide pyrophosphorylase	Biosynthesis of cofactors, prosthetic groups, and carriers | Pyridine nucleotides

NSE_RS02890,NRI_RS02980,NHE_RS04215	putative nicotinate (nicotinamide) nucleotide adenylyltransferase	Biosynthesis of cofactors, prosthetic groups, and carriers | Pyridine nucleotides
NSE_RS01330,NRI_RS01380,NHE_RS01340	pyridoxal phosphate biosynthetic protein PdxJ	Biosynthesis of cofactors, prosthetic groups, and carriers | Pyridoxine
NSE_RS01515,NRI_RS01565,NHE_RS01560	putative pyridoxamine 5-phosphate oxidase	Biosynthesis of cofactors, prosthetic groups, and carriers | Pyridoxine
NSE_RS00170,NRI_RS00155,NHE_RS00165	riboflavin biosynthesis protein RibF	Biosynthesis of cofactors, prosthetic groups, and carriers | Riboflavin, FMN, and FAD
NSE_RS00365,NRI_RS00360,NHE_RS00375	cytidine/deoxycytidylate deaminase family protein	Biosynthesis of cofactors, prosthetic groups, and carriers | Riboflavin, FMN, and FAD
NSE_RS01630,NRI_RS01680,NHE_RS01680	6,7-dimethyl-8-ribityllumazine synthase	Biosynthesis of cofactors, prosthetic groups, and carriers | Riboflavin, FMN, and FAD
NSE_RS02020,NRI_RS02065,NHE_RS02075	riboflavin biosynthesis protein RibD	Biosynthesis of cofactors, prosthetic groups, and carriers | Riboflavin, FMN, and FAD
NSE_RS02635,NRI_RS02705,NHE_RS02755	3,4-dihydroxy-2-butanone 4-phosphate synthase	Biosynthesis of cofactors, prosthetic groups, and carriers | Riboflavin, FMN, and FAD
NSE_RS03025,NRI_RS03115,NHE_RS03170	GTP cyclohydrolase II	Biosynthesis of cofactors, prosthetic groups, and carriers | Riboflavin, FMN, and FAD
NSE_RS03480,NRI_RS03560,NHE_RS03640	riboflavin synthase, alpha subunit	Biosynthesis of cofactors, prosthetic groups, and carriers | Riboflavin, FMN, and FAD
NSE_RS00190,NRI_RS00175,NHE_RS00185	thiamine biosynthesis protein ThiS	Biosynthesis of cofactors, prosthetic groups, and carriers | Thiamine
NSE_RS00875,NRI_RS00915,NHE_RS00870	putative thiamine-phosphate pyrophosphorylase	Biosynthesis of cofactors, prosthetic groups, and carriers | Thiamine
NSE_RS01955,NRI_RS02000,NHE_RS02005	thiamin biosynthesis ThiG	Biosynthesis of cofactors, prosthetic groups, and carriers | Thiamine
NSE_RS01995,NRI_RS02035,NHE_RS02040	coenzyme PQQ synthesis protein C	Biosynthesis of cofactors, prosthetic groups, and carriers | Thiamine
NSE_RS03880,NRI_RS03960,NHE_RS04080	putative phosphomethylpyrimidine kinase	Biosynthesis of cofactors, prosthetic groups, and carriers | Thiamine
**Cell envelope**		
NSE_RS01935,NRI_RS01980,NHE_RS01985	UDP-N-acetylmuramoyl-tripeptide--D-alanyl-D-alanine ligase truncation, partial	Cell envelope | Biosynthesis and degradation of murein sacculus and peptidoglycan
NSE_RS02415,NRI_RS02470,NHE_RS02520	putative UDP-N-acetylenolpyruvoylglucosamine reductase	Cell envelope | Biosynthesis and degradation of murein sacculus and peptidoglycan
NSE_RS03755,NRI_RS03840,NHE_RS03930	S-adenosyl-methyltransferase MraW	Cell envelope | Biosynthesis and degradation of murein sacculus and peptidoglycan
NSE_RS00820,NRI_RS00860,NHE_RS00815	exopolysaccharide synthesis protein	Cell envelope | Biosynthesis and degradation of surface polysaccharides and lipopolysaccharides
NSE_RS03845,NRI_RS03925,NHE_RS04040	undecaprenyl diphosphate synthase	Cell envelope | Biosynthesis and degradation of surface polysaccharides and lipopolysaccharides
NSE_RS00220,NRI_RS00205,NHE_RS00215	putative membrane protein	Cell envelope | Other
NSE_RS00295,NRI_RS00285,NHE_RS00295	putative membrane protein	Cell envelope | Other
NSE_RS00300,NRI_RS00290,NHE_RS00305	putative lipoprotein	Cell envelope | Other
NSE_RS00465,NRI_RS00510,NHE_RS00460	putative membrane protein	Cell envelope | Other
NSE_RS00815,NRI_RS00855,NHE_RS00810	hypothetical protein	Cell envelope | Other
NSE_RS00965,NRI_RS01005,NHE_RS00965	51 kDa major antigen (P51)	Cell envelope | Other
NSE_RS01635,NRI_RS01685,NHE_RS01685	inner membrane protein, 60 kDa	Cell envelope | Other

NSE_RS02220,NRI_RS02265,NHE_RS02300	putative membrane protein	Cell envelope | Other
NSE_RS02265,NRI_RS02310,NHE_RS02345	major surface protein	Cell envelope | Other
NSE_RS02305,NRI_RS02355,NHE_RS02395	membrane protein, MviN family	Cell envelope | Other
NSE_RS02355,NRI_RS02405,NHE_RS02445	putative membrane protein	Cell envelope | Other
NSE_RS02995,NRI_RS03085,NHE_RS03135	putative membrane protein	Cell envelope | Other
NSE_RS03220,NRI_RS03305,NHE_RS03385	membrane protein, TerC family	Cell envelope | Other
NSE_RS03550,NRI_RS03630,NHE_RS03715	*Neorickettsia* surface protein 1	Cell envelope | Other
NSE_RS03555,NRI_RS03635,NHE_RS03720	*Neorickettsia* surface protein 2	Cell envelope | Other
NSE_RS03560,NRI_RS03640,NHE_RS03725	*Neorickettsia* surface protein 3	Cell envelope | Other
NSE_RS03620,NRI_RS03705,NHE_RS03785	putative peptidoglycan-associated lipoprotein	Cell envelope | Other
NSE_RS03690,NRI_RS03775,NRI_RS03775,NRI_RS03775,NRI_RS03780,NRI_RS03780,NRI_RS03780,NRI_RS03785,NRI_RS03785,NHE_RS03855	strain-specific surface antigen	Cell envelope | Other
NSE_RS03700,NRI_RS03785,NRI_RS03775,NRI_RS03775,NRI_RS03775,NRI_RS03775,NRI_RS03780,NRI_RS03780,NRI_RS03780,NHE_RS03855	strain-specific surface antigen	Cell envelope | Other
NSE_RS03775,NRI_RS03860,NHE_RS03965,NRI_RS03865,NHE_RS03970	putative membrane protein	Cell envelope | Other
NSE_RS03780,NRI_RS03865,NHE_RS03970,NRI_RS03860,NHE_RS03965	putative membrane protein	Cell envelope | Other
NSE_RS03810,NRI_RS03890,NHE_RS04005	putative vacJ lipoprotein	Cell envelope | Other
**Cellular processes**		
NSE_RS00245,NRI_RS00230,NHE_RS00240	putative osmotically inducible protein	Cellular processes | Adaptations to atypical conditions
NSE_RS01530,NRI_RS01580,NHE_RS01580	acid phosphatase SurE	Cellular processes | Adaptations to atypical conditions
NSE_RS00015,NRI_RS00005,NHE_RS00025	chromosome partitioning protein, ParB family	Cellular processes | Cell division
NSE_RS00460,NRI_RS00505,NHE_RS00455	ribonuclease, Rne/Rng family	Cellular processes | Cell division
NSE_RS01420,NRI_RS01470,NHE_RS01450	cell division protein FtsZ	Cellular processes | Cell division
NSE_RS01730,NRI_RS01770,NHE_RS01770	cell division protein FtsA	Cellular processes | Cell division
NSE_RS02400,NRI_RS02455,NHE_RS02500	putative cell division protein	Cellular processes | Cell division
NSE_RS03905,NRI_RS03985,NHE_RS04110	GTP-binding protein Era	Cellular processes | Cell division
NSE_RS03990,NRI_RS04075,NHE_RS04210	putative cell division protein FtsK	Cellular processes | Cell division
NSE_RS02295,NRI_RS02340,NHE_RS02380	antioxidant, AhpC/Tsa family	Cellular processes | Detoxification
NSE_RS03430,NRI_RS03510,NHE_RS03595	superoxide dismutase, Fe	Cellular processes | Detoxification
NSE_RS01690,NRI_RS01740,NHE_RS04195,NHE_RS04190	putative competence protein F	Cellular processes | DNA transformation
NSE_RS03765,NRI_RS03850,NHE_RS03940	putative competence protein ComL	Cellular processes | DNA transformation
NSE_RS01610,NRI_RS01660,NHE_RS01660	ATP synthase F0, C chain	Cellular processes | Pathogenesis
NSE_RS03105,NRI_RS03190,NHE_RS03255	ATP synthase F1, epsilon subunit	Cellular processes | Pathogenesis
NSE_RS00290,NRI_RS00280,NHE_RS00290	drug resistance transporter, Bcr/CflA family	Cellular processes | Toxin production and resistance

NSE_RS00750,NRI_RS00790,NHE_RS00745	transporter, AcrB/AcrD/AcrF family	Cellular processes | Toxin production and resistance
NSE_RS00520,NRI_RS00565,NHE_RS00515	5,10-methenyltetrahydrofolate synthetase	Central intermediary metabolism | One-carbon metabolism
NSE_RS01800,NRI_RS01840,NHE_RS01845, NRI_RS02775,NHE_RS02800	oxidoreductase, short-chain dehydrogenase/reductase family	Central intermediary metabolism | Other
NSE_RS01975,NRI_RS02015,NHE_RS02020	S-adenosylmethionine synthetase	Central intermediary metabolism | Other
NSE_RS02685,NRI_RS02775,NHE_RS02800, NRI_RS01840	3-oxoacyl-[acyl-carrier protein] reductase	Central intermediary metabolism | Other
NSE_RS02980,NRI_RS03070,NHE_RS03110	inorganic pyrophosphatase	Central intermediary metabolism | Phosphorus compounds
**DNA metabolism**		
NSE_RS02595,NRI_RS02665,NHE_RS02715	DNA-binding protein HU	DNA metabolism | Chromosome-associated proteins
NSE_RS00560,NRI_RS00600,NHE_RS00545, NHE_RS01850,NRI_RS01845	tyrosine recombinase XerD	DNA metabolism | DNA replication, recombination, and repair
NSE_RS00620,NRI_RS00660,NHE_RS00605	DnaK suppressor protein	DNA metabolism | DNA replication, recombination, and repair
NSE_RS00645,NRI_RS00685,NHE_RS00630	DNA polymerase III, alpha subunit	DNA metabolism | DNA replication, recombination, and repair
NSE_RS00660,NRI_RS00700,NHE_RS00645	DNA polymerase III, beta subunit	DNA metabolism | DNA replication, recombination, and repair
NSE_RS00780,NRI_RS00820,NHE_RS00775	putative DNA replication and repair protein RecF	DNA metabolism | DNA replication, recombination, and repair
NSE_RS00810,NRI_RS00850,NHE_RS00805	primosomal protein N'	DNA metabolism | DNA replication, recombination, and repair
NSE_RS00915,NRI_RS04065,NHE_RS00910, NRI_RS04060	DNA repair protein RadC	DNA metabolism | DNA replication, recombination, and repair
NSE_RS00975,NRI_RS01015,NHE_RS00975	endonuclease III	DNA metabolism | DNA replication, recombination, and repair
NSE_RS01040,NRI_RS01080,NHE_RS01040	chromosomal replication initiator protein DnaA	DNA metabolism | DNA replication, recombination, and repair
NSE_RS01685,NRI_RS01735,NHE_RS01735	exodeoxyribonuclease III	DNA metabolism | DNA replication, recombination, and repair
NSE_RS01805,NRI_RS01845,NHE_RS01850, NRI_RS00600,NHE_RS00545	site-specific recombinase, phage integrase family	DNA metabolism | DNA replication, recombination, and repair
NSE_RS01855,NRI_RS01895,NHE_RS01900	putative DNA repair protein RecO	DNA metabolism | DNA replication, recombination, and repair
NSE_RS01885,NRI_RS01930,NHE_RS01930	ATP-dependent DNA helicase, UvrD/REP family	DNA metabolism | DNA replication, recombination, and repair
NSE_RS01895,NRI_RS01940,NHE_RS01945	DNA polymerase III, epsilon subunit	DNA metabolism | DNA replication, recombination, and repair
NSE_RS01990,NRI_RS02030,NHE_RS02035	putative DNA polymerase III, gamma/tau subunit	DNA metabolism | DNA replication, recombination, and repair
NSE_RS02025,NRI_RS02070,NHE_RS02080	DNA ligase, NAD-dependent	DNA metabolism | DNA replication, recombination, and repair
NSE_RS02170,NRI_RS02215,NHE_RS02250	recA protein	DNA metabolism | DNA replication, recombination, and repair
NSE_RS02360,NRI_RS02410,NHE_RS02455	holliday junction DNA helicase RuvA	DNA metabolism | DNA replication, recombination, and repair
NSE_RS02365,NRI_RS02415,NHE_RS02460	holliday junction DNA helicase RuvB	DNA metabolism | DNA replication, recombination, and repair
NSE_RS02430,NRI_RS02485,NHE_RS02535	DNA topoisomerase I	DNA metabolism | DNA replication, recombination, and repair
NSE_RS02625,NRI_RS02695,NHE_RS02745	polyA polymerase family protein	DNA metabolism | DNA replication, recombination, and repair
NSE_RS02720,NRI_RS02810,NHE_RS02840	DNA polymerase I	DNA metabolism | DNA replication, recombination, and repair
NSE_RS02795,NRI_RS02885,NHE_RS02915	ATP-dependent DNA helicase RecG	DNA metabolism | DNA replication, recombination, and repair
NSE_RS02895,NRI_RS02985,NHE_RS03020	single-stranded-DNA-specific exonuclease RecJ	DNA metabolism | DNA replication, recombination, and repair
NSE_RS02930,NRI_RS03020,NHE_RS03055	DNA gyrase, B subunit	DNA metabolism | DNA replication, recombination, and repair
NSE_RS03090,NRI_RS03175,NHE_RS03240	single-strand binding protein	DNA metabolism | DNA replication, recombination, and repair

NSE_RS03670,NRI_RS03755,NHE_RS03835	recombination protein RecR	DNA metabolism | DNA replication, recombination, and repair
NSE_RS03805,NRI_RS03885,NHE_RS04000	uracil-DNA glycosylase, family 4	DNA metabolism | DNA replication, recombination, and repair
NSE_RS03885,NRI_RS03965,NHE_RS04085	crossover junction endodeoxyribonuclease RuvC	DNA metabolism | DNA replication, recombination, and repair
NSE_RS03900,NRI_RS03980,NHE_RS04105	DNA gyrase, A subunit	DNA metabolism | DNA replication, recombination, and repair
NSE_RS03915,NRI_RS03995,NHE_RS04120	DNA primase	DNA metabolism | DNA replication, recombination, and repair
**<u>Energy metabolism</u>**		
NSE_RS00910,NRI_RS00950,NHE_RS00905	glycerol-3-phosphate dehydrogenase (NAD(P)+)	Energy metabolism | Aerobic
NSE_RS03315,NRI_RS03395,NHE_RS03480	propionyl-CoA carboxylase, alpha subunit	Energy metabolism | Amino acids and amines
NSE_RS00510,NRI_RS00555,NHE_RS00505, NRI_RS03195,NHE_RS03260	ATP synthase F1, alpha subunit	Energy metabolism | ATP-proton motive force interconversion
NSE_RS00515,NRI_RS00560,NHE_RS00510	ATP synthase F1, delta subunit	Energy metabolism | ATP-proton motive force interconversion
NSE_RS01605,NRI_RS01655,NHE_RS01655	ATP synthase F0, A subunit	Energy metabolism | ATP-proton motive force interconversion
NSE_RS01620,NRI_RS01670,NHE_RS01670	putative ATPase F0, B chain	Energy metabolism | ATP-proton motive force interconversion
NSE_RS02410,NRI_RS02465,NHE_RS02510	ATP synthase F1, gamma subunit	Energy metabolism | ATP-proton motive force interconversion
NSE_RS03110,NRI_RS03195,NHE_RS03260, NRI_RS00555,NHE_RS00505	ATP synthase F1, beta subunit	Energy metabolism | ATP-proton motive force interconversion
NSE_RS00060,NRI_RS00050,NHE_RS00065	NADH dehydrogenase I, J subunit	Energy metabolism | Electron transport
NSE_RS00065,NRI_RS00055,NHE_RS00070	NADH dehydrogenase I, K subunit	Energy metabolism | Electron transport
NSE_RS00070,NRI_RS00060,NHE_RS00075, NHE_RS02400,NRI_RS02360,NRI_RS02890, NHE_RS02920,NRI_RS02955,NHE_RS02990, NRI_RS02895,NHE_RS02925	NADH dehydrogenase I, L subunit	Energy metabolism | Electron transport
NSE_RS00235,NRI_RS00220,NHE_RS00230	NADH dehydrogenase I, G subunit	Energy metabolism | Electron transport
NSE_RS00240,NRI_RS00225,NHE_RS00235	NADH dehydrogenase I, H subunit	Energy metabolism | Electron transport
NSE_RS00905,NRI_RS00945,NHE_RS00900	thioredoxin	Energy metabolism | Electron transport
NSE_RS01035,NRI_RS01075,NHE_RS01035	cytochrome c oxidase assembly protein CtaG	Energy metabolism | Electron transport
NSE_RS01225,NRI_RS01270,NHE_RS01230	iron-sulfur cluster binding protein	Energy metabolism | Electron transport
NSE_RS01290,NRI_RS01335,NHE_RS01295	glutaredoxin 3	Energy metabolism | Electron transport
NSE_RS01370,NRI_RS01420,NHE_RS01380	ferredoxin	Energy metabolism | Electron transport
NSE_RS01545,NRI_RS01595,NHE_RS01595	quinone oxidoreductase	Energy metabolism | Electron transport
NSE_RS01550,NRI_RS01600,NHE_RS01600	putative oxidoreductase	Energy metabolism | Electron transport
NSE_RS01740,NRI_RS01780,NHE_RS01780	NADH dehydrogenase I, A subunit	Energy metabolism | Electron transport
NSE_RS01745,NRI_RS01785,NHE_RS01785	NADH dehydrogenase I, B subunit	Energy metabolism | Electron transport
NSE_RS01750,NRI_RS01790,NHE_RS01790	NADH dehydrogenase I, C subunit	Energy metabolism | Electron transport
NSE_RS01910,NRI_RS01955,NHE_RS01960	cytochrome c	Energy metabolism | Electron transport
NSE_RS02290,NRI_RS02335,NHE_RS02375, NHE_RS03310,NRI_RS03240	thioredoxin-disulfide reductase	Energy metabolism | Electron transport
NSE_RS02325,NRI_RS02375,NHE_RS02415	NADH dehydrogenase I, D subunit	Energy metabolism | Electron transport
NSE_RS02350,NRI_RS02400,NHE_RS02440	cytochrome c-type biogenesis protein, CcmF/CycK/CcsA family	Energy metabolism | Electron transport

NSE_RS02520,NRI_RS02580,NHE_RS02625	cytochrome c oxidase, subunit II	Energy metabolism | Electron transport
NSE_RS02525,NRI_RS02585,NHE_RS02630	cytochrome c oxidase, subunit I	Energy metabolism | Electron transport
NSE_RS02535,NRI_RS02595,NHE_RS02640	ubiquinol-cytochrome c reductase, iron-sulfur subunit	Energy metabolism | Electron transport
NSE_RS02540,NRI_RS02600,NHE_RS02645	ubiquinol-cytochrome c reductase, cytochrome b	Energy metabolism | Electron transport
NSE_RS02545,NRI_RS02605,NHE_RS02650	ubiquinol-cytochrome c reductase, cytochrome c1	Energy metabolism | Electron transport
NSE_RS02580,NRI_RS02650,NHE_RS02700	NADH dehydrogenase I, E subunit	Energy metabolism | Electron transport
NSE_RS02725,NRI_RS02815,NHE_RS02845	cytochrome c oxidase, subunit III	Energy metabolism | Electron transport
NSE_RS02310,NRI_RS02360,NHE_RS02400, NHE_RS02990,NHE_RS00075,NRI_RS00060, NRI_RS02955,NHE_RS02920,NRI_RS02890	NADH-ubiquinone/plastoquinone oxidoreductase family protein	Energy metabolism | Electron transport
NSE_RS02800,NRI_RS02890,NHE_RS02920, NHE_RS00075,NHE_RS02400,NRI_RS02955, NRI_RS00060,NRI_RS02360,NHE_RS02990, NHE_RS02925	NADH dehydrogenase I, M subunit	Energy metabolism | Electron transport
NSE_RS02805,NRI_RS02895,NHE_RS02925, NHE_RS02990,NRI_RS02955,NRI_RS00060, NHE_RS00075,NHE_RS02920	NADH dehydrogenase I, N subunit	Energy metabolism | Electron transport
NSE_RS02865,NRI_RS02955,NHE_RS02990, NRI_RS02360,NRI_RS02890,NHE_RS02920, NHE_RS02400,NHE_RS00075,NHE_RS02925 ,NRI_RS00060,NRI_RS02895	NADH-ubiquinone/plastoquinone oxidoreductase family protein	Energy metabolism | Electron transport
NSE_RS02900,NRI_RS02990,NHE_RS03025	NADH dehydrogenase I, F subunit	Energy metabolism | Electron transport
NSE_RS03155,NRI_RS03240,NHE_RS03310, NHE_RS02375,NRI_RS02335	pyridine nucleotide-disulphide oxidoreductase family protein	Energy metabolism | Electron transport
NSE_RS03335,NRI_RS03415,NHE_RS03500	NADH dehydrogenase I, I subunit	Energy metabolism | Electron transport
NSE_RS03375,NRI_RS03455,NHE_RS03555	putative cytochrome c-type biogenesis protein CcmE	Energy metabolism | Electron transport
NSE_RS03490,NRI_RS03570,NHE_RS03650	putative cytochrome oxidase assembly protein	Energy metabolism | Electron transport
NSE_RS03645,NRI_RS03730,NHE_RS03810	thioredoxin 1	Energy metabolism | Electron transport
NSE_RS03790,NRI_RS03875,NHE_RS03985	cytochrome b561 family protein	Energy metabolism | Electron transport
NSE_RS00555,NRI_RS00595,NHE_RS00550	putative fructose-bisphosphate aldolase, class I	Energy metabolism | Glycolysis/gluconeogenesis
NSE_RS01015,NRI_RS01055,NHE_RS01015	triosephosphate isomerase	Energy metabolism | Glycolysis/gluconeogenesis
NSE_RS01760,NRI_RS01800,NHE_RS01800	glyceraldehyde-3-phosphate dehydrogenase, type I	Energy metabolism | Glycolysis/gluconeogenesis
NSE_RS01765,NRI_RS01805,NHE_RS01805	phosphoglycerate kinase	Energy metabolism | Glycolysis/gluconeogenesis
NSE_RS02975,NRI_RS03065,NHE_RS03105	enolase	Energy metabolism | Glycolysis/gluconeogenesis
NSE_RS03630,NRI_RS03715,NHE_RS03795	2,3-bisphosphoglycerate-independent phosphoglycerate mutase	Energy metabolism | Glycolysis/gluconeogenesis
NSE_RS01510,NRI_RS01560,NHE_RS01555	pyruvate, phosphate dikinase	Energy metabolism | Other
NSE_RS00860,NRI_RS00900,NHE_RS00855	ribose 5-phosphate isomerase B	Energy metabolism | Pentose phosphate pathway
NSE_RS01395,NRI_RS01445,NHE_RS01420	ribulose-phosphate 3-epimerase	Energy metabolism | Pentose phosphate pathway
NSE_RS02860,NRI_RS02950,NHE_RS02985, NHE_RS02985	transketolase	Energy metabolism | Pentose phosphate pathway
NSE_RS03100,NRI_RS03185,NHE_RS03250	putative transaldolase	Energy metabolism | Pentose phosphate pathway
NSE_RS01865,NRI_RS01910,NHE_RS01915, NHE_RS02820,NRI_RS02795	dihydrolipoamide dehydrogenase	Energy metabolism | Pyruvate dehydrogenase
NSE_RS02705,NRI_RS02795,NHE_RS02820, NHE_RS01915,NRI_RS01910	dihydrolipoamide dehydrogenase	Energy metabolism | Pyruvate dehydrogenase

NSE_RS03030,NRI_RS03120,NHE_RS03185	putative pyruvate dehydrogenase complex, E1 component, beta subunit	Energy metabolism | Pyruvate dehydrogenase
NSE_RS03255,NRI_RS03335,NHE_RS03420	pyruvate dehydrogenase complex, E1 component, pyruvate dehydrogenase alpha subunit	Energy metabolism | Pyruvate dehydrogenase
NSE_RS00205,NRI_RS00190,NHE_RS00200	succinate dehydrogenase, cytochrome b556 subunit	Energy metabolism | TCA cycle
NSE_RS00210,NRI_RS00195,NHE_RS00205	putative succinate dehydrogenase, hydrophobic membrane anchor protein	Energy metabolism | TCA cycle
NSE_RS00250,NRI_RS00235,NHE_RS00245	fumarate hydratase, class II	Energy metabolism | TCA cycle
NSE_RS00670,NRI_RS00710,NHE_RS00655	dehydrogenase, isocitrate/isopropylmalate family	Energy metabolism | TCA cycle
NSE_RS00995,NRI_RS01035,NHE_RS00995	succinyl-CoA synthetase, alpha subunit	Energy metabolism | TCA cycle
NSE_RS01000,NRI_RS01040,NHE_RS01000	succinyl-CoA synthetase, beta subunit	Energy metabolism | TCA cycle
NSE_RS02185,NRI_RS02230,NHE_RS02265	succinate dehydrogenase and fumarate reductase iron-sulfur protein	Energy metabolism | TCA cycle
NSE_RS02255,NRI_RS02300,NHE_RS02335, NHE_RS04075,NRI_RS03955	2-oxoglutarate dehydrogenase, E2 component, dihydrolipoamide succinyltransferase	Energy metabolism | TCA cycle
NSE_RS02370,NRI_RS02420,NHE_RS02465	2-oxoglutarate dehydrogenase, E1 component	Energy metabolism | TCA cycle
NSE_RS02445,NRI_RS02500,NHE_RS02550	aconitate hydratase 1	Energy metabolism | TCA cycle
NSE_RS02965,NRI_RS03055,NHE_RS03090	citrate synthase	Energy metabolism | TCA cycle
NSE_RS03875,NRI_RS03955,NHE_RS04075, NRI_RS02300,NHE_RS02335	pyruvate dehydrogenase complex, E2 component, dihydrolipoamide acetyltransferase	Energy metabolism | TCA cycle
NSE_RS03890,NRI_RS03970,NHE_RS04090	malate dehydrogenase, NAD-dependent	Energy metabolism | TCA cycle
**<u>Fatty acid and phospholipid metabolism</u>**		
NSE_RS00055,NRI_RS00045,NHE_RS00060	CDP-diacylglycerol--glycerol-3-phosphate 3-phosphatidyltransferase	Fatty acid and phospholipid metabolism | Biosynthesis
NSE_RS00185,NRI_RS00170,NHE_RS00180	enoyl-(acyl-carrier-protein) reductase	Fatty acid and phospholipid metabolism | Biosynthesis
NSE_RS00675,NRI_RS00715,NHE_RS00660	putative transporter	Fatty acid and phospholipid metabolism | Biosynthesis
NSE_RS00980,NRI_RS01020,NHE_RS00980	putative CDP-diacylglycerol--serine O-phosphatidyltransferase	Fatty acid and phospholipid metabolism | Biosynthesis
NSE_RS01650,NRI_RS01700,NHE_RS01700	1-acyl-sn-glycerol-3-phosphate acyltransferase family protein	Fatty acid and phospholipid metabolism | Biosynthesis
NSE_RS01820,NRI_RS01860,NHE_RS01865	acyl carrier protein	Fatty acid and phospholipid metabolism | Biosynthesis
NSE_RS01825,NRI_RS01865,NHE_RS01870	3-oxoacyl-(acyl-carrier-protein) synthase II	Fatty acid and phospholipid metabolism | Biosynthesis
NSE_RS02235,NRI_RS02280,NHE_RS02315	enoyl-(acyl-carrier-protein) reductase II	Fatty acid and phospholipid metabolism | Biosynthesis
NSE_RS02565,NRI_RS02625,NHE_RS02680	3-oxoacyl-(acyl-carrier-protein) synthase III	Fatty acid and phospholipid metabolism | Biosynthesis
NSE_RS02570,NRI_RS02635,NHE_RS02685	fatty acid/phospholipid synthesis protein PlsX	Fatty acid and phospholipid metabolism | Biosynthesis
NSE_RS02675,NRI_RS02765,NHE_RS02785	beta-hydroxyacyl-[acyl carrier protein] dehydratase FabZ	Fatty acid and phospholipid metabolism | Biosynthesis
NSE_RS03840,NRI_RS03920,NHE_RS04035	putative phosphatidate cytidylyltransferase	Fatty acid and phospholipid metabolism | Biosynthesis
NSE_RS03910,NRI_RS03990,NHE_RS04115	malonyl CoA-acyl carrier protein transacylase	Fatty acid and phospholipid metabolism | Biosynthesis
NSE_RS03920,NRI_RS04000,NHE_RS04125	holo-(acyl-carrier protein) synthase	Fatty acid and phospholipid metabolism | Biosynthesis
NSE_RS00900,NRI_RS00940,NHE_RS00895	putative phosphatidylglycerophosphatase A	Fatty acid and phospholipid metabolism | Degradation
NSE_RS00970,NRI_RS01010,NHE_RS00970	conserved hypothetical protein	Fatty acid and phospholipid metabolism | Degradation

NSE_RS01200,NRI_RS01245,NHE_RS01205	phosphatidylglycerophosphatase A	Fatty acid and phospholipid metabolism | Degradation
NSE_RS03230,NRI_RS03310,NHE_RS03390	propionyl-CoA carboxylase, beta subunit	Fatty acid and phospholipid metabolism | Degradation
NSE_RS03535,NRI_RS03615,NHE_RS03700	patatin-like phospholipase family protein	Fatty acid and phospholipid metabolism | Degradation
**Protein Fate**		
**Sec-dependent pathway:**		
NSE_RS02215,NRI_RS02260,NHE_RS02295	signal recognition particle protein SRP	Protein fate | Protein and peptide secretion and trafficking
NSE_RS02240,NRI_RS02285,NHE_RS02320	signal recognition particle-docking protein FtsY	Protein fate | Protein and peptide secretion and trafficking
NSE_RS00925,NRI_RS00965,NHE_RS00920	preprotein translocase, SecA subunit	Protein fate | Protein and peptide secretion and trafficking
NSE_RS01475,NRI_RS01525,NHE_RS01515	putative protein-export protein SecB	Protein fate | Protein and peptide secretion and trafficking
NSE_RS02765,NRI_RS02855,NHE_RS02885	preprotein translocase, SecE subunit	Protein fate | Protein and peptide secretion and trafficking
NSE_RS01165,NRI_RS01210,NHE_RS01170	preprotein translocase, SecY subunit	Protein fate | Protein and peptide secretion and trafficking
NSE_RS03565,NRI_RS03645,NHE_RS03730	preprotein translocase, SecG subunit	Protein fate | Protein and peptide secretion and trafficking
NSE_RS01700,NRI_RS01745,NHE_RS01745	putative protein-export membrane protein SecF	Protein fate | Protein and peptide secretion and trafficking
NSE_RS02550,NRI_RS02610,NHE_RS02655	protein-export membrane protein SecD	Protein fate | Protein and peptide secretion and trafficking
NSE_RS01325,NRI_RS01375,NHE_RS01335	preprotein translocase, YajC subunit	Protein fate | Protein and peptide secretion and trafficking
**Tat pathway:**		
NSE_RS01950,NRI_RS01995,NHE_RS02000	twin-arginine translocation protein, TatA/E family	Protein fate | Protein and peptide secretion and trafficking
NSE_RS02090,NRI_RS02135,NHE_RS02160	twin-arginine translocation protein, TatB	Protein fate | Protein and peptide secretion and trafficking
NSE_RS00495,NRI_RS00540,NHE_RS00490	Sec-independent protein translocase TatC	Protein fate | Protein and peptide secretion and trafficking
**T1SS:**		
NSE_RS03825,NRI_RS03905,NHE_RS04020	type I secretion membrane fusion protein, HlyD family	Protein fate | Protein and peptide secretion and trafficking
NSE_RS00180,NRI_RS00165,NHE_RS00175	type I secretion system ATPase HlyB	Protein fate | Protein and peptide secretion and trafficking
NSE_RS03240,NRI_RS03320,NHE_RS03400	outer membrane efflux protein TolC	Protein fate | Protein and peptide secretion and trafficking || Transport and binding proteins | Unknown substrate
**T4SS:**		
NSE_RS03000,NRI_RS03090,NHE_RS03145	type IV secretion system protein VirD4	Protein fate | Protein and peptide secretion and trafficking
NSE_RS03005,NRI_RS03095,NHE_RS03150	type IV secretion system protein VirB11	Protein fate | Protein and peptide secretion and trafficking
NSE_RS03010,NRI_RS03100,NHE_RS03155	type IV secretion system protein VirB10	Protein fate | Protein and peptide secretion and trafficking
NSE_RS03015,NRI_RS03105,NHE_RS03160	type IV secretion system protein VirB9 (VirB9-1)	Protein fate | Protein and peptide secretion and trafficking
NSE_RS03020,NRI_RS03110,NHE_RS03165	type IV secretion system protein VirB8 (VirB8-1)	Protein fate | Protein and peptide secretion and trafficking
NSE_RS03120,NRI_RS03205,NHE_RS03270	type IV secretion system protein VirB4 (VirB4-2)	Protein fate | Protein and peptide secretion and trafficking
NSE_RS03125,NRI_RS03210,NHE_RS03285	type IV secretion system protein VirB2 (VirB2-2)	Protein fate | Protein and peptide secretion and trafficking
NSE_RS03130,NRI_RS03215,NHE_RS03285	type IV secretion system protein VirB2 (VirB2-1)	Protein fate | Protein and peptide secretion and trafficking
NSE_RS00825,NRI_RS00865,NHE_RS00820	type IV secretion system protein VirB9 (VirB9-2)	Protein fate | Protein and peptide secretion and trafficking

NSE_RS00830,NRI_RS00870,NHE_RS00825	type IV secretion system protein VirB8 (VirB8-2)	Protein fate | Protein and peptide secretion and trafficking
NSE_RS03500,NRI_RS03580,NHE_RS03665	type IV secretion system protein, VirB6 family (VirB6-4)	Protein fate | Protein and peptide secretion and trafficking
NSE_RS03505,NRI_RS03585,NHE_RS03670	type IV secretion system protein, VirB6 family (VirB6-3)	Protein fate | Protein and peptide secretion and trafficking
NSE_RS03510,NRI_RS03590,NHE_RS03675	type IV secretion system protein, VirB6 family (VirB6-2)	Protein fate | Protein and peptide secretion and trafficking
NSE_RS03515,NRI_RS03595,NHE_RS03680	type IV secretion system protein VirB6 (VirB6-1)	Protein fate | Protein and peptide secretion and trafficking
NSE_RS03520,NRI_RS03600,NHE_RS03685	type IV secretion system protein VirB4 (VirB4-1)	Protein fate | Protein and peptide secretion and trafficking
NSE_RS04020,NRI_RS04090,NHE_RS03236	type IV secretion system protein VirB7	Protein fate | Protein and peptide secretion and trafficking
NSE_RS03525,NRI_RS03605,NHE_RS03690	type IV secretion system protein VirB3	Protein fate | Protein and peptide secretion and trafficking
**Chaperones:**		
NSE_RS02605,NRI_RS02675,NHE_RS02725	60 kDa chaperonin GroEL	Protein fate | Protein folding and stabilization
NSE_RS02610,NRI_RS02680,NHE_RS02730	10 kDa chaperonin GroES	Protein fate | Protein folding and stabilization
NSE_RS02190,NRI_RS02235,NHE_RS02270	chaperone protein DnaJ	Protein fate | Protein folding and stabilization
NSE_RS03330,NRI_RS03410,NHE_RS03495	DnaJ domain protein	Protein fate | Protein folding and stabilization
NSE_RS00085,NRI_RS00075,NHE_RS00095	chaperone protein DnaK	Protein fate | Protein folding and stabilization
NSE_RS01235,NRI_RS01280,NHE_RS01240	putative chaperone protein HscB	Protein fate | Protein folding and stabilization
NSE_RS00835,NRI_RS00875,NHE_RS00830	co-chaperone GrpE	Protein fate | Protein folding and stabilization
NSE_RS02000,NRI_RS02040,NHE_RS02045	heat shock protein HtpG	Protein fate | Protein folding and stabilization
NSE_RS01230,NRI_RS01275,NHE_RS01235	putative chaperone protein HscA	Protein fate | Protein folding and stabilization
**Other functions:**		
NSE_RS00630,NRI_RS00670,NHE_RS00615, NHE_RS00620	HflK protein	Protein fate | Degradation of proteins, peptides, and glycopeptides
NSE_RS00635,NRI_RS00675,NHE_RS00620	HflC protein	Protein fate | Degradation of proteins, peptides, and glycopeptides
NSE_RS00680,NRI_RS00720,NHE_RS00670, NHE_RS03895,NRI_RS03805,NRI_RS03800, NHE_RS03890	peptidase, M16 family	Protein fate | Degradation of proteins, peptides, and glycopeptides
NSE_RS00940,NRI_RS00980,NHE_RS00935	putative metalloendopeptidase, glycoprotease family	Protein fate | Degradation of proteins, peptides, and glycopeptides
NSE_RS01440,NRI_RS01490,NHE_RS01475	ATP-dependent protease La	Protein fate | Degradation of proteins, peptides, and glycopeptides
NSE_RS01660,NRI_RS01710,NHE_RS01710	signal peptide peptidase SppA, 36K type	Protein fate | Degradation of proteins, peptides, and glycopeptides
NSE_RS01720,NRI_RS01760,NHE_RS01760	ATP-dependent metalloprotease FtsH	Protein fate | Degradation of proteins, peptides, and glycopeptides
NSE_RS01900,NRI_RS01945,NHE_RS01950	metallopeptidase, M24 family	Protein fate | Degradation of proteins, peptides, and glycopeptides
NSE_RS01920,NRI_RS01965,NHE_RS01970	cytosol aminopeptidase	Protein fate | Degradation of proteins, peptides, and glycopeptides
NSE_RS02920,NRI_RS03010,NHE_RS03045	putative membrane-associated zinc metalloprotease	Protein fate | Degradation of proteins, peptides, and glycopeptides
NSE_RS03055,NRI_RS03145,NHE_RS03210	ATP-dependent Clp protease, proteolytic subunit ClpP	Protein fate | Degradation of proteins, peptides, and glycopeptides
NSE_RS03435,NRI_RS03515,NHE_RS03600	glycoprotease family protein	Protein fate | Degradation of proteins, peptides, and glycopeptides
NSE_RS03720,NRI_RS03800,NHE_RS03890, NHE_RS00670,NRI_RS00720	peptidase, M16 family	Protein fate | Degradation of proteins, peptides, and glycopeptides
NSE_RS03725,NRI_RS03805,NHE_RS03895	peptidase, M16 family	Protein fate | Degradation of proteins, peptides, and glycopeptides

NSE_RS03735,NRI_RS03815,NHE_RS03905	putative carboxypeptidase	Protein fate | Degradation of proteins, peptides, and glycopeptides
NSE_RS01310,NRI_RS01360,NHE_RS01315, NHE_RS02955,NRI_RS02925,NHE_RS01995, NRI_RS01990,NHE_RS03450,NRI_RS03360, NRI_RS03610,NHE_RS02960	putative lipoprotein releasing system ATP-binding protein LolD	Protein fate | Protein and peptide secretion and trafficking
NSE_RS01665,NRI_RS01715,NHE_RS01715, NRI_RS03610,NHE_RS03695,NHE_RS01315, NHE_RS02955,NRI_RS03360,NHE_RS03450	putative ABC transporter, ATP-binding/permease protein	Protein fate | Protein and peptide secretion and trafficking
NSE_RS01945,NRI_RS01990,NHE_RS01995, NHE_RS03450,NRI_RS01360,NRI_RS01715, NHE_RS03695	ABC transporter, ATP-binding protein	Protein fate | Protein and peptide secretion and trafficking
NSE_RS02835,NRI_RS02925,NHE_RS02955, NRI_RS01990,NHE_RS03450,NRI_RS03360, NHE_RS01315,NHE_RS01995,NRI_RS01360, NHE_RS03695,NRI_RS03610	ABC transporter, ATP-binding protein	Protein fate | Protein and peptide secretion and trafficking
NSE_RS02885,NRI_RS02975,NHE_RS03010	conserved hypothetical protein	Protein fate | Protein and peptide secretion and trafficking
NSE_RS02915,NRI_RS03005,NHE_RS03040	outer membrane protein, OMP85 family	Protein fate | Protein and peptide secretion and trafficking
NSE_RS03175,NRI_RS03260,NHE_RS03335	signal peptidase I	Protein fate | Protein and peptide secretion and trafficking
NSE_RS03285,NRI_RS03360,NHE_RS03450, NRI_RS01990,NHE_RS01995,NHE_RS02955, NRI_RS02925,NHE_RS01315,NHE_RS03695, NRI_RS01360,NRI_RS03610	putative phosphate ABC transporter, ATP-binding protein	Protein fate | Protein and peptide secretion and trafficking
NSE_RS03530,NRI_RS03610,NHE_RS03695, NHE_RS01715,NHE_RS00175,NRI_RS00165, NRI_RS01715,NHE_RS01995,NRI_RS02925, NRI_RS03360,NHE_RS02955	putative ABC transporter, ATP-binding protein/permease protein	Protein fate | Protein and peptide secretion and trafficking
NSE_RS03730,NRI_RS03810,NHE_RS03900	signal peptidase II	Protein fate | Protein and peptide secretion and trafficking
NSE_RS00455,NRI_RS00500,NHE_RS00450, NHE_RS01305,NHE_RS01305,NRI_RS01350	ClpB protein	Protein fate | Protein folding and stabilization
NSE_RS00640,NRI_RS00680,NHE_RS00625	periplasmic serine protease, DO/DeqQ family	Protein fate | Protein folding and stabilization
NSE_RS00685,NRI_RS00725,NHE_RS00675	heat shock protein HslVU, HslV subunit	Protein fate | Protein folding and stabilization
NSE_RS00690,NRI_RS00730,NHE_RS00680, NHE_RS03215,NHE_RS03215,NRI_RS03150, NRI_RS03150	heat shock protein HslVU, ATPase subunit HslU	Protein fate | Protein folding and stabilization
NSE_RS01300,NRI_RS01350,NHE_RS01305, NHE_RS00450,NHE_RS00450,NRI_RS00500	ATP-dependent Clp protease, ATP-binding subunit ClpA	Protein fate | Protein folding and stabilization
NSE_RS01410,NRI_RS04070,NHE_RS01440	disulfide bond formation protein, DsbB family	Protein fate | Protein folding and stabilization
NSE_RS02620,NRI_RS02690,NHE_RS02740	rotamase family protein	Protein fate | Protein folding and stabilization
NSE_RS03050,NRI_RS03140,NHE_RS03205	putative trigger factor	Protein fate | Protein folding and stabilization
NSE_RS03060,NRI_RS03150,NHE_RS03215, NHE_RS00680,NHE_RS00680,NRI_RS00730	ATP-dependent Clp protease, ATP-binding subunit ClpX	Protein fate | Protein folding and stabilization
NSE_RS03470,NRI_RS03550,NHE_RS03630	peptidyl-prolyl cis-trans isomerase, cyclophilin-type	Protein fate | Protein folding and stabilization
NSE_RS03600,NRI_RS03685,NHE_RS03765	conserved hypothetical protein	Protein fate | Protein folding and stabilization
NSE_RS01400,NRI_RS01450,NHE_RS01425	methionine aminopeptidase, type I	Protein fate | Protein modification and repair
NSE_RS01585,NRI_RS01635,NHE_RS01635	peptide deformylase	Protein fate | Protein modification and repair
NSE_RS02010,NRI_RS02055,NHE_RS02065	apolipoprotein N-acyltransferase	Protein fate | Protein modification and repair
NSE_RS02810,NRI_RS02900,NHE_RS02930	biotin--acetyl-CoA-carboxylase ligase	Protein fate | Protein modification and repair
NSE_RS03485,NRI_RS03565,NHE_RS03645	prolipoprotein diacylglyceryl transferase	Protein fate | Protein modification and repair
NSE_RS03680,NRI_RS03765,NHE_RS03845	disulfide oxidoreductase	Protein fate | Protein modification and repair

NSE_RS00890,NRI_RS00930,NHE_RS00885, NRI_RS02305	TldD protein	Protein fate | Other
NSE_RS02260,NRI_RS02305,NHE_RS02340, NHE_RS00885,NRI_RS00930	pmbA protein	Protein fate | Other
**Protein synthesis**		
NSE_RS01270,NRI_RS01315,NHE_RS01275	peptidyl-tRNA hydrolase	Protein synthesis | Other
NSE_RS01795,NRI_RS01835,NHE_RS03870, NRI_RS03790	GTP-binding protein Obg/CgtA	Protein synthesis | Other
NSE_RS03295,NRI_RS03375,NHE_RS03460	SsrA-binding protein	Protein synthesis | Other
NSE_RS03705,NRI_RS03790,NHE_RS03870, NRI_RS01835	GTP-binding protein YchF	Protein synthesis | Other
NSE_RS00275,NRI_RS00265,NHE_RS00275	ribosomal protein S18	Protein synthesis | Ribosomal proteins: synthesis and modification
NSE_RS00845,NRI_RS00885,NHE_RS00840	ribosomal protein L35	Protein synthesis | Ribosomal proteins: synthesis and modification
NSE_RS00260,NRI_RS00250,NHE_RS00260	ribosomal protein S15	Protein synthesis | Ribosomal proteins: synthesis and modification
NSE_RS00270,NRI_RS00260,NHE_RS00270	ribosomal protein L9	Protein synthesis | Ribosomal proteins: synthesis and modification
NSE_RS00280,NRI_RS00270,NHE_RS00280	ribosomal protein S6	Protein synthesis | Ribosomal proteins: synthesis and modification
NSE_RS00475,NRI_RS00520,NHE_RS00470	ribosomal protein S16	Protein synthesis | Ribosomal proteins: synthesis and modification
NSE_RS00850,NRI_RS00890,NHE_RS00845	ribosomal protein L20	Protein synthesis | Ribosomal proteins: synthesis and modification
NSE_RS01030,NRI_RS01070,NHE_RS01030	ribosomal protein L33	Protein synthesis | Ribosomal proteins: synthesis and modification
NSE_RS01065,NRI_RS01110,NHE_RS01070	ribosomal protein S10	Protein synthesis | Ribosomal proteins: synthesis and modification
NSE_RS01070,NRI_RS01115,NHE_RS01075	ribosomal protein L3	Protein synthesis | Ribosomal proteins: synthesis and modification
NSE_RS01075,NRI_RS01120,NHE_RS01080	ribosomal protein L4	Protein synthesis | Ribosomal proteins: synthesis and modification
NSE_RS01080,NRI_RS01125,NHE_RS01085	ribosomal protein L23	Protein synthesis | Ribosomal proteins: synthesis and modification
NSE_RS01085,NRI_RS01130,NHE_RS01090	ribosomal protein L2	Protein synthesis | Ribosomal proteins: synthesis and modification
NSE_RS01090,NRI_RS01135,NHE_RS01095	ribosomal protein S19	Protein synthesis | Ribosomal proteins: synthesis and modification
NSE_RS01095,NRI_RS01140,NHE_RS01100	ribosomal protein L22	Protein synthesis | Ribosomal proteins: synthesis and modification
NSE_RS01100,NRI_RS01145,NHE_RS01105	ribosomal protein S3	Protein synthesis | Ribosomal proteins: synthesis and modification
NSE_RS01105,NRI_RS01150,NHE_RS01110	ribosomal protein L16	Protein synthesis | Ribosomal proteins: synthesis and modification
NSE_RS01115,NRI_RS01160,NHE_RS01120	ribosomal protein S17	Protein synthesis | Ribosomal proteins: synthesis and modification
NSE_RS01120,NRI_RS01165,NHE_RS01125	ribosomal protein L14	Protein synthesis | Ribosomal proteins: synthesis and modification
NSE_RS01125,NRI_RS01170,NHE_RS01130	ribosomal protein L24	Protein synthesis | Ribosomal proteins: synthesis and modification
NSE_RS01130,NRI_RS01175,NHE_RS01135	ribosomal protein L5	Protein synthesis | Ribosomal proteins: synthesis and modification
NSE_RS01135,NRI_RS01180,NHE_RS01140	ribosomal protein S14	Protein synthesis | Ribosomal proteins: synthesis and modification
NSE_RS01140,NRI_RS01185,NHE_RS01145	ribosomal protein S8	Protein synthesis | Ribosomal proteins: synthesis and modification
NSE_RS01145,NRI_RS01190,NHE_RS01150	ribosomal protein L6	Protein synthesis | Ribosomal proteins: synthesis and modification
NSE_RS01150,NHE_RS01155,NRI_RS01195	ribosomal protein L18	Protein synthesis | Ribosomal proteins: synthesis and modification
NSE_RS01155,NRI_RS01200,NHE_RS01160	ribosomal protein S5	Protein synthesis | Ribosomal proteins: synthesis and modification
NSE_RS01160,NRI_RS01205,NHE_RS01165	ribosomal protein L15	Protein synthesis | Ribosomal proteins: synthesis and modification

NSE_RS01175,NRI_RS01220,NHE_RS01180	ribosomal protein S13	Protein synthesis | Ribosomal proteins: synthesis and modification
NSE_RS01180,NRI_RS01225,NHE_RS01185	ribosomal protein S11	Protein synthesis | Ribosomal proteins: synthesis and modification
NSE_RS01190,NRI_RS01235,NHE_RS01195	ribosomal protein L17	Protein synthesis | Ribosomal proteins: synthesis and modification
NSE_RS01265,NRI_RS01310,NHE_RS01270	ribosomal 5S rRNA E-loop binding protein Ctc/L25/TL5	Protein synthesis | Ribosomal proteins: synthesis and modification
NSE_RS01335,NRI_RS01385,NHE_RS01345	ribosomal protein L28	Protein synthesis | Ribosomal proteins: synthesis and modification
NSE_RS01655,NRI_RS01705,NHE_RS01705	ribosomal protein S1	Protein synthesis | Ribosomal proteins: synthesis and modification
NSE_RS02040,NRI_RS02085,NHE_RS02095	conserved hypothetical protein	Protein synthesis | Ribosomal proteins: synthesis and modification
NSE_RS02395,NRI_RS02450,NHE_RS02490	ribosomal protein S4	Protein synthesis | Ribosomal proteins: synthesis and modification
NSE_RS02740,NRI_RS02830,NHE_RS02860	ribosomal protein L7/L12	Protein synthesis | Ribosomal proteins: synthesis and modification
NSE_RS02745,NRI_RS02835,NHE_RS02865	50S ribosomal protein L10	Protein synthesis | Ribosomal proteins: synthesis and modification
NSE_RS02750,NRI_RS02840,NHE_RS02870	ribosomal protein L1	Protein synthesis | Ribosomal proteins: synthesis and modification
NSE_RS02755,NRI_RS02845,NHE_RS02875	ribosomal protein L11	Protein synthesis | Ribosomal proteins: synthesis and modification
NSE_RS02785,NRI_RS02875,NHE_RS02905	ribosomal protein S7	Protein synthesis | Ribosomal proteins: synthesis and modification
NSE_RS02790,NRI_RS02880,NHE_RS02910	ribosomal protein S12	Protein synthesis | Ribosomal proteins: synthesis and modification
NSE_RS03210,NRI_RS03295,NHE_RS03370	ribosomal protein S20	Protein synthesis | Ribosomal proteins: synthesis and modification
NSE_RS03370,NRI_RS03450,NHE_RS03550	ribosomal protein S21	Protein synthesis | Ribosomal proteins: synthesis and modification
NSE_RS03410,NRI_RS03490,NHE_RS03575	ribosomal protein S9	Protein synthesis | Ribosomal proteins: synthesis and modification
NSE_RS03415,NRI_RS03495,NHE_RS03580	ribosomal protein L13	Protein synthesis | Ribosomal proteins: synthesis and modification
NSE_RS03650,NRI_RS03735,NHE_RS03815	ribosomal protein L19	Protein synthesis | Ribosomal proteins: synthesis and modification
NSE_RS03660,NRI_RS03745,NHE_RS03825	ribosomal protein L27	Protein synthesis | Ribosomal proteins: synthesis and modification
NSE_RS03665,NRI_RS03750,NHE_RS03830	ribosomal protein L21	Protein synthesis | Ribosomal proteins: synthesis and modification
NSE_RS00765,NRI_RS00805,NHE_RS00760	translation elongation factor P	Protein synthesis | Translation factors
NSE_RS01205,NRI_RS01250,NHE_RS01210	translation initiation factor IF-3	Protein synthesis | Translation factors
NSE_RS01425,NRI_RS01475,NHE_RS01460	ribosomal subunit interface protein	Protein synthesis | Translation factors
NSE_RS01645,NRI_RS01695,NHE_RS01695, NHE_RS02565	peptide chain release factor 1	Protein synthesis | Translation factors
NSE_RS02130,NRI_RS02175,NHE_RS02205, NRI_RS03220,NHE_RS03290,NRI_RS02805, NHE_RS02895,NRI_RS02865	translation initiation factor IF-2	Protein synthesis | Translation factors
NSE_RS02715,NRI_RS02805,NHE_RS02835, NHE_RS03290,NRI_RS03220,NHE_RS02895, NRI_RS02865,NHE_RS02900,NRI_RS02870, NRI_RS02870,NRI_RS02175,NHE_RS02205	GTP-binding protein TypA	Protein synthesis | Translation factors
NSE_RS02775,NRI_RS02865,NHE_RS02895, NRI_RS02805,NHE_RS02835,NRI_RS03220, NRI_RS02175	translation elongation factor Tu	Protein synthesis | Translation factors
NSE_RS02780,NRI_RS02870,NHE_RS02900, NHE_RS02835,NHE_RS02835,NRI_RS02805, NRI_RS02805,NHE_RS03290,NHE_RS03290, NRI_RS03220,NRI_RS03220	translation elongation factor G	Protein synthesis | Translation factors
NSE_RS03135,NRI_RS03220,NHE_RS03290, NHE_RS02835,NRI_RS02805,NHE_RS02900, NHE_RS02900,NRI_RS02870,NRI_RS02870, NHE_RS02205,NHE_RS02895,NRI_RS02175	GTP-binding protein LepA	Protein synthesis | Translation factors

NSE_RS03595,NRI_RS03675,NHE_RS03760	translation initiation factor IF-1	Protein synthesis | Translation factors
NSE_RS03850,NRI_RS03930,NHE_RS04045	ribosome recycling factor	Protein synthesis | Translation factors
NSE_RS03860,NRI_RS03940,NHE_RS04055	translation elongation factor Ts	Protein synthesis | Translation factors
NSE_RS00215,NRI_RS00200,NHE_RS00210	arginyl-tRNA synthetase	Protein synthesis | tRNA aminoacylation
NSE_RS00360,NRI_RS00355,NHE_RS00370	glutamyl-tRNA(Gln) amidotransferase, B subunit	Protein synthesis | tRNA aminoacylation
NSE_RS00385,NRI_RS00385,NHE_RS00385	methionyl-tRNA formyltransferase	Protein synthesis | tRNA aminoacylation
NSE_RS00600,NRI_RS00640,NHE_RS00585	alanyl-tRNA synthetase	Protein synthesis | tRNA aminoacylation
NSE_RS00840,NRI_RS00880,NHE_RS00835	tryptophanyl-tRNA synthetase	Protein synthesis | tRNA aminoacylation
NSE_RS00855,NRI_RS00895,NHE_RS00850	phenylalanyl-tRNA synthetase, alpha subunit	Protein synthesis | tRNA aminoacylation
NSE_RS01025,NRI_RS01065,NHE_RS01025	glutamyl-tRNA(Gln) amidotransferase, A subunit	Protein synthesis | tRNA aminoacylation
NSE_RS01210,NRI_RS01255,NHE_RS01215	threonyl-tRNA synthetase	Protein synthesis | tRNA aminoacylation
NSE_RS01380,NRI_RS01430,NHE_RS01390	cysteinyl-tRNA synthetase	Protein synthesis | tRNA aminoacylation
NSE_RS01430,NRI_RS01480,NHE_RS01465	tyrosyl-tRNA synthetase	Protein synthesis | tRNA aminoacylation
NSE_RS01500,NRI_RS01550,NHE_RS01540, NHE_RS01215	prolyl-tRNA synthetase	Protein synthesis | tRNA aminoacylation
NSE_RS01725,NRI_RS01765,NHE_RS01765	putative phenylalanyl-tRNA synthetase, beta subunit	Protein synthesis | tRNA aminoacylation
NSE_RS01930,NRI_RS01975,NHE_RS01980	aspartyl-tRNA synthetase	Protein synthesis | tRNA aminoacylation
NSE_RS02055,NRI_RS02100,NHE_RS02110, NHE_RS02560,NRI_RS02510	leucyl-tRNA synthetase	Protein synthesis | tRNA aminoacylation
NSE_RS02100,NRI_RS02145,NHE_RS02170	putative glutamyl-tRNA(Gln) amidotransferase, C subunit	Protein synthesis | tRNA aminoacylation
NSE_RS02110,NHE_RS02180,NHE_RS03125 ,NRI_RS03080	glutamyl-tRNA synthetase	Protein synthesis | tRNA aminoacylation
NSE_RS02200,NRI_RS02245,NHE_RS02280, NRI_RS02510,NHE_RS02560	isoleucyl-tRNA synthetase	Protein synthesis | tRNA aminoacylation
NSE_RS02335,NRI_RS02385,NHE_RS02425	seryl-tRNA synthetase	Protein synthesis | tRNA aminoacylation
NSE_RS02455,NRI_RS02510,NHE_RS02560, NHE_RS02560,NHE_RS02280,NRI_RS02245, NRI_RS02100,NHE_RS02110	putative valyl-tRNA synthetase	Protein synthesis | tRNA aminoacylation
NSE_RS02990,NRI_RS03080,NHE_RS03125, NHE_RS02180	glutamyl-tRNA synthetase	Protein synthesis | tRNA aminoacylation
NSE_RS03075,NRI_RS03160,NHE_RS03225	glycyl-tRNA synthetase, beta subunit	Protein synthesis | tRNA aminoacylation
NSE_RS03080,NRI_RS03165,NHE_RS03230	glycyl-tRNA synthetase, alpha subunit	Protein synthesis | tRNA aminoacylation
NSE_RS03140,NRI_RS03225,NHE_RS03295	lysyl-tRNA synthetase	Protein synthesis | tRNA aminoacylation
NSE_RS03160,NRI_RS03245,NHE_RS03315	histidyl-tRNA synthetase	Protein synthesis | tRNA aminoacylation
NSE_RS03640,NRI_RS03725,NHE_RS03805	methionyl-tRNA synthetase	Protein synthesis | tRNA aminoacylation
NSE_RS00080,NRI_RS00070,NHE_RS00085	queuine tRNA-ribosyltransferase	Protein synthesis | tRNA and rRNA base modification
NSE_RS00090,NRI_RS00080,NHE_RS00100	tRNA pseudouridine synthase A	Protein synthesis | tRNA and rRNA base modification
NSE_RS00405,NRI_RS00410,NHE_RS00410	tRNA pseudouridine synthase B	Protein synthesis | tRNA and rRNA base modification
NSE_RS00570,NRI_RS00610,NHE_RS00535, NHE_RS02370,NRI_RS02330	ribosomal large subunit pseudouridine synthases, RluA family	Protein synthesis | tRNA and rRNA base modification
NSE_RS01050,NRI_RS01095,NHE_RS01055	RNA methyltransferase, TrmH family, group 3	Protein synthesis | tRNA and rRNA base modification
NSE_RS01485,NRI_RS01535,NHE_RS01525	dimethyladenosine transferase	Protein synthesis | tRNA and rRNA base modification

NSE_RS01890,NRI_RS01935,NHE_RS01940	tRNA (5-methylaminomethyl-2-thiouridylate)-methyltransferase	Protein synthesis | tRNA and rRNA base modification
NSE_RS02245,NRI_RS02290,NHE_RS02325	ribosomal RNA large subunit methyltransferase J	Protein synthesis | tRNA and rRNA base modification
NSE_RS02285,NRI_RS02330,NHE_RS02370, NHE_RS00535,NRI_RS00610	ribosomal large subunit pseudouridine synthase C	Protein synthesis | tRNA and rRNA base modification
NSE_RS02340,NRI_RS02390,NHE_RS02430	tRNA delta(2)-isopentenylpyrophosphate transferase	Protein synthesis | tRNA and rRNA base modification
NSE_RS02870,NRI_RS02960,NHE_RS02995	glucose inhibited division protein A	Protein synthesis | tRNA and rRNA base modification
NSE_RS03655,NRI_RS03740,NHE_RS03820	tRNA (guanine-N1)-methyltransferase	Protein synthesis | tRNA and rRNA base modification
NSE_RS03870,NRI_RS03950,NHE_RS04065	ubiquinone/menaquinone biosynthesis methlytransferase UbiE	Protein synthesis | tRNA and rRNA base modification
**Purines, pyrimidines, nucleosides, and nucleotides biosynthesis**		
NSE_RS00625,NRI_RS00665,NHE_RS00610	thymidylate synthase, flavin-dependent	Purines, pyrimidines, nucleosides, and nucleotides | 2'-Deoxyribonucleotide metabolism
NSE_RS01670,NRI_RS01720,NHE_RS01720	ribonucleoside-diphosphate reductase, alpha subunit	Purines, pyrimidines, nucleosides, and nucleotides | 2'-Deoxyribonucleotide metabolism
NSE_RS02120,NRI_RS02165,NHE_RS02190	ribonucleoside-diphosphate reductase, beta subunit	Purines, pyrimidines, nucleosides, and nucleotides | 2'-Deoxyribonucleotide metabolism
NSE_RS03895,NRI_RS03975,NHE_RS04095	deoxyuridine 5'triphosphate nucleotidohydrolase	Purines, pyrimidines, nucleosides, and nucleotides | 2'-Deoxyribonucleotide metabolism
NSE_RS03930,NRI_RS04010,NHE_RS04140	putative deoxycytidine triphosphate deaminase	Purines, pyrimidines, nucleosides, and nucleotides | 2'-Deoxyribonucleotide metabolism
NSE_RS01170,NRI_RS01215,NHE_RS01175	adenylate kinase	Purines, pyrimidines, nucleosides, and nucleotides | Nucleotide and nucleoside interconversions
NSE_RS01850,NRI_RS01890,NHE_RS01895	putative deoxyguanosinetriphosphate triphosphohydrolase	Purines, pyrimidines, nucleosides, and nucleotides | Nucleotide and nucleoside interconversions
NSE_RS02250,NRI_RS02295,NHE_RS02330	thymidylate kinase	Purines, pyrimidines, nucleosides, and nucleotides | Nucleotide and nucleoside interconversions
NSE_RS02300,NRI_RS02345,NHE_RS02390	nucleoside diphosphate kinase	Purines, pyrimidines, nucleosides, and nucleotides | Nucleotide and nucleoside interconversions
NSE_RS02950,NRI_RS03040,NHE_RS03075	guanylate kinase	Purines, pyrimidines, nucleosides, and nucleotides | Nucleotide and nucleoside interconversions
NSE_RS03855,NRI_RS03935,NHE_RS04050	uridylate kinase	Purines, pyrimidines, nucleosides, and nucleotides | Nucleotide and nucleoside interconversions
NSE_RS00130,NRI_RS00115,NHE_RS00125	phosphoribosylformylglycinamidine cyclo-ligase	Purines, pyrimidines, nucleosides, and nucleotides | Purine ribonucleotide biosynthesis
NSE_RS00265,NRI_RS00255,NHE_RS00265	adenylosuccinate lyase	Purines, pyrimidines, nucleosides, and nucleotides | Purine ribonucleotide biosynthesis
NSE_RS00725,NRI_RS00760,NHE_RS00715	phosphoribosylaminoimidazolecarboxamide formyltransferase/IMP cyclohydrolase	Purines, pyrimidines, nucleosides, and nucleotides | Purine ribonucleotide biosynthesis
NSE_RS00755,NRI_RS00795,NHE_RS00750, NHE_RS02150	amidophosphoribosyltransferase	Purines, pyrimidines, nucleosides, and nucleotides | Purine ribonucleotide biosynthesis
NSE_RS00895,NRI_RS00935,NHE_RS00890	adenylosuccinate synthetase	Purines, pyrimidines, nucleosides, and nucleotides | Purine ribonucleotide biosynthesis
NSE_RS00935,NRI_RS00975,NHE_RS00930	phosphoribosylaminoimidazole carboxylase, catalytic subunit	Purines, pyrimidines, nucleosides, and nucleotides | Purine ribonucleotide biosynthesis

NSE_RS01445,NRI_RS01495,NHE_RS01480	conserved hypothetical protein	Purines, pyrimidines, nucleosides, and nucleotides | Purine ribonucleotide biosynthesis
NSE_RS01810,NRI_RS01850,NHE_RS01855	putative phosphoribosylformylglycinamidine synthase I	Purines, pyrimidines, nucleosides, and nucleotides | Purine ribonucleotide biosynthesis
NSE_RS01915,NRI_RS01960,NHE_RS01965	phosphoribosylglycinamide formyltransferase	Purines, pyrimidines, nucleosides, and nucleotides | Purine ribonucleotide biosynthesis
NSE_RS02145,NRI_RS02190,NHE_RS02220	inosine-5'-monophosphate dehydrogenase	Purines, pyrimidines, nucleosides, and nucleotides | Purine ribonucleotide biosynthesis
NSE_RS03300,NRI_RS03380,NHE_RS03465	putative phosphoribosylformylglycinamidine synthase II	Purines, pyrimidines, nucleosides, and nucleotides | Purine ribonucleotide biosynthesis
NSE_RS03320,NRI_RS03400,NHE_RS03485	ribose-phosphate pyrophosphokinase	Purines, pyrimidines, nucleosides, and nucleotides | Purine ribonucleotide biosynthesis
NSE_RS03475,NRI_RS03555,NHE_RS03635	phosphoribosylaminoimidazole-succinocarboxamide synthase	Purines, pyrimidines, nucleosides, and nucleotides | Purine ribonucleotide biosynthesis
NSE_RS03610,NRI_RS03695,NHE_RS03775	GMP synthase	Purines, pyrimidines, nucleosides, and nucleotides | Purine ribonucleotide biosynthesis
NSE_RS03770,NRI_RS03855,NHE_RS03945	phosphoribosylamine--glycine ligase	Purines, pyrimidines, nucleosides, and nucleotides | Purine ribonucleotide biosynthesis
NSE_RS03935,NRI_RS04015,NHE_RS04150	phosphoribosylaminoimidazole carboxylase, ATPase subunit	Purines, pyrimidines, nucleosides, and nucleotides | Purine ribonucleotide biosynthesis
NSE_RS00595,NRI_RS00635,NHE_RS00580	dihydroorotase, multifunctional complex type	Purines, pyrimidines, nucleosides, and nucleotides | Pyrimidine ribonucleotide biosynthesis
NSE_RS00700,NRI_RS00740,NHE_RS00690	dihydroorotate dehydrogenase	Purines, pyrimidines, nucleosides, and nucleotides | Pyrimidine ribonucleotide biosynthesis
NSE_RS00880,NRI_RS00920,NHE_RS00875	carbamoyl-phosphate synthase, large subunit	Purines, pyrimidines, nucleosides, and nucleotides | Pyrimidine ribonucleotide biosynthesis
NSE_RS02035,NRI_RS02080,NHE_RS02090	carbamoyl-phosphate synthase, small subunit	Purines, pyrimidines, nucleosides, and nucleotides | Pyrimidine ribonucleotide biosynthesis
NSE_RS02075,NRI_RS02120,NHE_RS02130	aspartate carbamoyltransferase	Purines, pyrimidines, nucleosides, and nucleotides | Pyrimidine ribonucleotide biosynthesis
NSE_RS02205,NRI_RS02250,NHE_RS02285	orotate phosphoribosyltransferase	Purines, pyrimidines, nucleosides, and nucleotides | Pyrimidine ribonucleotide biosynthesis
NSE_RS03215,NRI_RS03300,NHE_RS03380	orotidine 5'-phosphate decarboxylase	Purines, pyrimidines, nucleosides, and nucleotides | Pyrimidine ribonucleotide biosynthesis
NSE_RS03570,NRI_RS03650,NHE_RS03735	CTP synthase	Purines, pyrimidines, nucleosides, and nucleotides | Pyrimidine ribonucleotide biosynthesis
**Regulatory functions**		
NSE_RS00025,NRI_RS00015,NHE_RS00035	sensor histidine kinase PleC	Regulatory functions | Protein interactions
NSE_RS02175,NRI_RS02220,NHE_RS02255	Sensor histidine kinase, PleC-like	Regulatory functions | Protein interactions
NSE_RS02085,NRI_RS02130,NHE_RS02155	response regulator/GGDEF domain protein PleD	Regulatory functions | Other
NSE_RS01495,NRI_RS01545,NHE_RS01535	Sensor histidine kinase/response regulator, CckA	Regulatory functions | Protein interactions
NSE_RS00930,NRI_RS00970,NHE_RS00925	DNA-binding response regulator CtrA	Regulatory functions | DNA interactions
NSE_RS01785,NRI_RS01825,NHE_RS01830	EAL domain protein	Regulatory functions | Other

NSE_RS03985,NRI_RS02020,NHE_RS04205	Transposase and inactivated derivatives	Regulatory functions | Other
NSE_RS01195,NRI_RS01240,NHE_RS01200	transcriptional regulator, MerR family protein	Regulatory functions | DNA interactions
NSE_RS01460,NRI_RS01510,NHE_RS01495	ATP cone domain protein	Regulatory functions | DNA interactions
NSE_RS03325,NRI_RS03405,NHE_RS03490	NifU-like domain protein	Regulatory functions | Other
**Transcription**		
NSE_RS01295,NRI_RS01345,NHE_RS01300	RNA polymerase sigma factor RpoD	Transcription | Transcription factors
NSE_RS01415,NRI_RS01465,NHE_RS01445	RNA polymerase sigma-32 factor RpoH	Transcription | Transcription factors
NSE_RS00160,NRI_RS00145,NHE_RS00155	Neorickettsia expression regulator NhxR	Transcription | Transcription factors
NSE_RS00920,NRI_RS00960,NHE_RS00915	putative transcriptional regulator Tr1	Transcription | Transcription factors
NSE_RS02065,NRI_RS02110,NHE_RS02120	SOS-response transcriptional repressor LexA	Unknown function | General
NSE_RS02690,NRI_RS02780,NHE_RS02805	ribonuclease HI	Transcription | Degradation of RNA
NSE_RS02850,NRI_RS02940,NHE_RS02970	ribonuclease HII	Transcription | Degradation of RNA
NSE_RS01185,NRI_RS01230,NHE_RS01190	DNA-directed RNA polymerase, alpha subunit	Transcription | DNA-dependent RNA polymerase
NSE_RS02735,NRI_RS02825,NHE_RS02855	DNA-directed RNA polymerase, beta subunit	Transcription | DNA-dependent RNA polymerase
NSE_RS03975,NRI_RS00150,NHE_RS00160	DNA-directed RNA polymerase, omega subunit	Transcription | DNA-dependent RNA polymerase
NSE_RS03380,NRI_RS03460,NHE_RS03560	metallo-beta-lactamase family, beta-CASP subfamily	Transcription | Other
NSE_RS00480,NRI_RS00525,NHE_RS00475	putative 16S rRNA processing protein RimM	Transcription | RNA processing
NSE_RS02135,NRI_RS02180,NHE_RS02210	putative ribosome-binding factor A	Transcription | RNA processing
NSE_RS02165,NRI_RS02210,NHE_RS02245	3'-5' exonuclease family protein	Transcription | RNA processing
NSE_RS03495,NRI_RS03575,NHE_RS04225	ribonuclease P protein component	Transcription | RNA processing
NSE_RS03745,NRI_RS03830,NHE_RS03915	ribonuclease III	Transcription | RNA processing
NSE_RS00305,NRI_RS00295,NHE_RS00310	transcription termination factor Rho	Transcription | Transcription factors
NSE_RS02125,NRI_RS02170,NHE_RS02200	N utilization substance protein A	Transcription | Transcription factors
NSE_RS02450,NRI_RS02505,NHE_RS02555	conserved hypothetical protein	Transcription | Transcription factors
NSE_RS02700,NRI_RS02790,NHE_RS02815	transcription elongation factor GreA	Transcription | Transcription factors
NSE_RS02760,NRI_RS02850,NHE_RS02880	putative transcription termination/antitermination factor NusG	Transcription | Transcription factors
NSE_RS03585,NRI_RS03665,NHE_RS03750	putative N utilization substance protein B	Transcription | Transcription factors
**Transport and binding proteins**		
NSE_RS01435,NRI_RS01485,NHE_RS01470	**bacterioferritin**	Transport and binding proteins | Cations and iron carrying compounds
NSE_RS00590,NRI_RS00630,NHE_RS00575	sodium:alanine symporter family protein	Transport and binding proteins | Amino acids, peptides and amines
NSE_RS02940,NRI_RS03030,NHE_RS03065	putative sodium:proline symporter	Transport and binding proteins | Amino acids, peptides and amines
NSE_RS00285,NRI_RS00275,NHE_RS00285	putative phosphate ABC transporter, periplasmic phosphate-binding protein	Transport and binding proteins | Anions
NSE_RS00800,NRI_RS00840,NHE_RS00795, NHE_RS01990,NRI_RS01985	phosphate ABC transporter, permease protein PstC	Transport and binding proteins | Anions
NSE_RS01940,NRI_RS01985,NHE_RS01990, NRI_RS00840,NHE_RS00795	phosphate ABC transporter, permease protein PstA	Transport and binding proteins | Anions

NSE_RS00035,NRI_RS00025,NHE_RS00045	Fe(3+) ABC transporter substrate-binding protein	Transport and binding proteins | Cations and iron carrying compounds
NSE_RS00135,NRI_RS00120,NHE_RS00130	Na(+)/H(+) antiporter subunit C	Transport and binding proteins | Cations and iron carrying compounds
NSE_RS00140,NRI_RS00125,NHE_RS00135	multisubunit Na+/H+ antiporter, MnhB subunit	Transport and binding proteins | Cations and iron carrying compounds
NSE_RS00145,NRI_RS00130,NHE_RS00140	multisubunit Na+/H+ antiporter, MnhB subunit	Transport and binding proteins | Cations and iron carrying compounds
NSE_RS00150,NRI_RS00135,NHE_RS00145	monovalent cation/proton antiporter, MnhG/PhaG subunit	Transport and binding proteins | Cations and iron carrying compounds
NSE_RS01875,NRI_RS01920,NHE_RS01920	magnesium transporter	Transport and binding proteins | Cations and iron carrying compounds
NSE_RS03605,NRI_RS03690,NHE_RS03770	glutathione-regulated potassium-efflux system protein	Transport and binding proteins | Cations and iron carrying compounds
NSE_RS00535,NRI_RS00580,NHE_RS00530	putative permease	Transport and binding proteins | Other
NSE_RS02070,NRI_RS02115,NHE_RS02125	heme exporter protein, CcmC family	Transport and binding proteins | Other
NSE_RS00585,NRI_RS00625,NHE_RS00570	efflux transporter, RND family, MFP subunit	Transport and binding proteins | Unknown substrate
NSE_RS00605,NRI_RS00645,NHE_RS00590	putative transporter	Transport and binding proteins | Unknown substrate
NSE_RS00715,NRI_RS00755,NHE_RS00705	Multiple resistance and pH regulation protein (MrpF/PhaF)	Transport and binding proteins | Unknown substrate
NSE_RS00745,NRI_RS00785,NHE_RS00740	permease, PerM family	Transport and binding proteins | Unknown substrate
NSE_RS00775,NRI_RS00815,NHE_RS00770	putative transporter	Transport and binding proteins | Unknown substrate
NSE_RS00945,NRI_RS00985,NHE_RS00940	TRAP transporter solute receptor, TAXI family	Transport and binding proteins | Unknown substrate
NSE_RS01260,NRI_RS01305,NHE_RS01265	putative transporter	Transport and binding proteins | Unknown substrate
NSE_RS01285,NRI_RS01330,NHE_RS01290	putative ATP-NAD kinase	Transport and binding proteins | Unknown substrate
NSE_RS01615,NRI_RS01665,NHE_RS01665	ATP synthase F0, B' chain	Transport and binding proteins | Unknown substrate
NSE_RS02225,NRI_RS02270,NHE_RS02305	RDD family protein	Transport and binding proteins | Unknown substrate
NSE_RS02830,NRI_RS02920,NHE_RS02950	putative ABC transporter, permease protein	Transport and binding proteins | Unknown substrate
NSE_RS02840,NRI_RS02930,NHE_RS02960, NHE_RS02960	ABC transporter, ATP-binding protein	Transport and binding proteins | Unknown substrate
NSE_RS03170,NRI_RS03255,NHE_RS03325	major facilitator family transporter	Transport and binding proteins | Unknown substrate
NSE_RS03310,NRI_RS03390,NHE_RS03475	putative permease	Transport and binding proteins | Unknown substrate
NSE_RS03425,NRI_RS03505,NHE_RS03590	TRAP transporter, 4TM/12TM fusion protein	Transport and binding proteins | Unknown substrate
NSE_RS03445,NRI_RS03525,NHE_RS03605	putative membrane protein	Transport and binding proteins | Unknown substrate
NSE_RS03450,NRI_RS03530,NHE_RS03610	mechanosensitive ion channel family protein	Transport and binding proteins | Unknown substrate
**Unknown functions**		
NSE_RS02155,NRI_RS02200,NHE_RS02235	mce-related protein	Unclassified | Role category not yet assigned
NSE_RS00195,NRI_RS00180,NHE_RS00190	hexapeptide transferase family protein	Unknown function | Enzymes of unknown specificity
NSE_RS00735,NRI_RS00775,NHE_RS00730	conserved hypothetical protein	Unknown function | Enzymes of unknown specificity
NSE_RS00785,NRI_RS00825,NHE_RS00780	putative methyltransferase	Unknown function | Enzymes of unknown specificity
NSE_RS01320,NRI_RS01370,NHE_RS01330	aminomethyl transferase family protein	Unknown function | Enzymes of unknown specificity
NSE_RS01470,NRI_RS01520,NHE_RS01510	hydrolase, TatD family	Unknown function | Enzymes of unknown specificity
NSE_RS01565,NRI_RS01615,NHE_RS01615	NADH-ubiquinone oxidoreductase family protein	Unknown function | Enzymes of unknown specificity

NSE_RS01905,NRI_RS01950,NHE_RS01955	putative hydrolase	Unknown function | Enzymes of unknown specificity
NSE_RS02095,NRI_RS02140,NHE_RS02165	NAD-glutamate dehydrogenase family protein	Unknown function | Enzymes of unknown specificity
NSE_RS02230,NRI_RS02275,NHE_RS02310	S-adenosylmethionine-dependent methyltransferases	Unknown function | Enzymes of unknown specificity
NSE_RS02380,NRI_RS02430,NHE_RS02475	metallo-beta-lactamase family protein	Unknown function | Enzymes of unknown specificity
NSE_RS02440,NRI_RS02495,NHE_RS02545	O-methyltransferase family protein	Unknown function | Enzymes of unknown specificity
NSE_RS02660,NRI_RS02750,NHE_RS02770	acetyltransferase, GNAT family	Unknown function | Enzymes of unknown specificity
NSE_RS02695,NRI_RS02785,NHE_RS02810	conserved hypothetical protein	Unknown function | Enzymes of unknown specificity
NSE_RS03280,NRI_RS03355,NHE_RS03440	flavin reductase family protein	Unknown function | Enzymes of unknown specificity
NSE_RS03355,NRI_RS03435,NHE_RS03530	Ser/Thr protein phosphatase family protein	Unknown function | Enzymes of unknown specificity
NSE_RS03545,NRI_RS03625,NHE_RS03710	hydrolase, alpha/beta fold family	Unknown function | Enzymes of unknown specificity
NSE_RS03800,NRI_RS03880,NHE_RS03995	HAD-superfamily hydrolase, subfamily IA, variant 1	Unknown function | Enzymes of unknown specificity
NSE_RS02935,NRI_RS03025,NHE_RS03060	Alkyl hydroperoxide reductase subunit AhpC (bacterioferritin comigratory protein)	Transport and binding proteins | Cations and iron carrying compounds
NSE_RS00075,NRI_RS00065,NHE_RS00080	ankyrin repeat protein	Unknown function | General
NSE_RS00485,NRI_RS00530,NHE_RS00480	RmuC domain protein	Unknown function | General
NSE_RS00500,NRI_RS00545,NHE_RS00495	modification methylase, HemK family	Unknown function | General
NSE_RS00505,NRI_RS00550,NHE_RS00500	hypothetical protein	Unknown function | General
NSE_RS00790,NRI_RS00830,NHE_RS00785	BolA family protein	Unknown function | General
NSE_RS00795,NRI_RS00835,NHE_RS00790	glutaredoxin-related protein	Unknown function | General
NSE_RS00955,NRI_RS00995,NHE_RS00955	putative membrane protein	Unknown function | General
NSE_RS01020,NRI_RS01060,NHE_RS01020	rhodanese domain protein	Unknown function | General
NSE_RS01315,NRI_RS01365,NHE_RS01325, NHE_RS04175	ComEC/Rec2 family protein	Unknown function | General
NSE_RS01345,NRI_RS01395,NHE_RS01355	aromatic rich family protein	Unknown function | General
NSE_RS01360,NRI_RS01410,NHE_RS01370	CBS/transporter associated domain protein	Unknown function | General
NSE_RS01525,NRI_RS01575,NHE_RS01575	putative tRNA-dihydrouridine synthase	Unknown function | General
NSE_RS01600,NRI_RS01650,NHE_RS01650	YihY family protein	Unknown function | General
NSE_RS01640,NRI_RS01690,NHE_RS01690	CBS domain protein	Unknown function | General
NSE_RS01680,NRI_RS01730,NHE_RS01730	HIT domain protein	Unknown function | General
NSE_RS02005,NRI_RS02050,NHE_RS02050, NRI_RS02045,NHE_RS02055	ankyrin repeat protein	Unknown function | General
NSE_RS02105,NRI_RS02150,NHE_RS02175	hypothetical protein	Unknown function | General
NSE_RS02195,NRI_RS02240,NHE_RS02275, NRI_RS03000,NHE_RS03035,NHE_RS03035	putative GTP-binding protein EngA	Unknown function | General
NSE_RS02375,NRI_RS02425,NHE_RS02470	Sua5/YciO/YrdC/YwlC family protein	Unknown function | General
NSE_RS02425,NRI_RS02480,NHE_RS02530	fructose-1,6-bisphosphatase, class II	Unknown function | General
NSE_RS02910,NRI_RS03000,NHE_RS03035, NHE_RS03035,NRI_RS02240,NHE_RS02275, NHE_RS02275	tRNA modification GTPase TrmE	Unknown function | General
NSE_RS02945,NRI_RS03035,NHE_RS03070	pentapeptide repeat domain protein	Unknown function | General

NSE_RS03095,NRI_RS03180,NHE_RS03245	conserved hypothetical protein	Unknown function | General
NSE_RS03145,NRI_RS03230,NHE_RS03300	hypothetical protein	Unknown function | General
NSE_RS03165,NRI_RS03250,NHE_RS03320	BolA family protein	Unknown function | General
NSE_RS03250,NRI_RS03330,NHE_RS03410	inositol monophosphatase family protein	Unknown function | General
NSE_RS03420,NRI_RS03500,NHE_RS03585	CvpA family protein	Unknown function | General
NSE_RS03460,NRI_RS03540,NHE_RS03620	class II aldolase/adducin domain protein	Unknown function | General
NSE_RS03615,NRI_RS03700,NHE_RS03780	ATP-binding protein, Mrp/Nbp35 family	Unknown function | General
NSE_RS03750,NRI_RS03835,NHE_RS03920	iojap-related protein	Unknown function | General
NSE_RS03835,NRI_RS03915,NHE_RS04030	conserved hypothetical protein	Unknown function | General
NSE_RS03980,NRI_RS00695,NHE_RS04170	Smr domain protein	Unknown function | General
NSE_RS00020,NRI_RS00010,NHE_RS00030	hypothetical protein	Hypothetical proteins | Conserved
NSE_RS00030,NRI_RS00020,NHE_RS00040	hypothetical protein	Hypothetical proteins | Conserved
NSE_RS00040,NRI_RS00030,NHE_RS00050	conserved hypothetical protein	Hypothetical proteins | Conserved
NSE_RS00105,NRI_RS00090,NHE_RS00105	Ankyrin-repeat protein	Hypothetical proteins | Conserved
NSE_RS00110,NRI_RS00100,NHE_RS00110	hypothetical protein	Hypothetical proteins | Conserved
NSE_RS00115,NRI_RS00105,NHE_RS00115	hypothetical protein	Hypothetical proteins | Conserved
NSE_RS00125,NRI_RS00110,NHE_RS00120	putative membrane protein	Hypothetical proteins | Conserved
NSE_RS00155,NRI_RS00140,NHE_RS00150	conserved hypothetical protein	Hypothetical proteins | Conserved
NSE_RS00175,NRI_RS00160,NHE_RS00170	hypothetical protein	Hypothetical proteins | Conserved
NSE_RS00230,NRI_RS00215,NHE_RS00225	conserved hypothetical protein	Hypothetical proteins | Conserved
NSE_RS00490,NRI_RS00535,NHE_RS00485	conserved hypothetical protein	Hypothetical proteins | Conserved
NSE_RS00710,NRI_RS00750,NHE_RS00700	hypothetical protein	Hypothetical proteins | Conserved
NSE_RS00740,NRI_RS00780,NHE_RS00735	conserved hypothetical protein	Hypothetical proteins | Conserved
NSE_RS00770,NRI_RS00810,NHE_RS00765	putative lipoprotein	Hypothetical proteins | Conserved
NSE_RS00805,NRI_RS00845,NHE_RS00800	hypothetical protein	Hypothetical proteins | Conserved
NSE_RS00960,NRI_RS01000,NHE_RS00960	conserved hypothetical protein	Hypothetical proteins | Conserved
NSE_RS00990,NRI_RS01030,NHE_RS00990	conserved hypothetical protein TIGR00043	Hypothetical proteins | Conserved
NSE_RS01220,NRI_RS01265,NHE_RS01225	hypothetical protein	Hypothetical proteins | Conserved
NSE_RS01305,NRI_RS01355,NHE_RS01310	hypothetical protein	Hypothetical proteins | Conserved
NSE_RS01350,NRI_RS01400,NHE_RS01360	conserved hypothetical protein	Hypothetical proteins | Conserved
NSE_RS01365,NRI_RS01415,NHE_RS04180	hypothetical protein	Hypothetical proteins | Conserved
NSE_RS01375,NRI_RS01425,NHE_RS01385	conserved hypothetical protein	Hypothetical proteins | Conserved
NSE_RS01450,NRI_RS01500,NHE_RS01485	putative membrane protein	Hypothetical proteins | Conserved
NSE_RS01480,NRI_RS01530,NHE_RS01520	Tim44-like domain protein	Hypothetical proteins | Conserved

NSE_RS01490,NRI_RS01540,NHE_RS01530	hypothetical protein	Hypothetical proteins | Conserved
NSE_RS01520,NRI_RS01570,NHE_RS01570	hypothetical protein	Hypothetical proteins | Conserved
NSE_RS01540,NRI_RS01590,NHE_RS01590	hypothetical protein	Hypothetical proteins | Conserved
NSE_RS01575,NRI_RS01625,NHE_RS01625	conserved hypothetical protein	Hypothetical proteins | Conserved
NSE_RS01625,NRI_RS01675,NHE_RS01675	hypothetical protein	Hypothetical proteins | Conserved
NSE_RS01735,NRI_RS01775,NHE_RS01775	hypothetical protein	Hypothetical proteins | Conserved
NSE_RS01775,NRI_RS01815,NHE_RS01820	hypothetical protein	Hypothetical proteins | Conserved
NSE_RS01780,NRI_RS01820,NHE_RS01825	hypothetical protein	Hypothetical proteins | Conserved
NSE_RS01815,NRI_RS01855,NHE_RS01860	hypothetical protein	Hypothetical proteins | Conserved
NSE_RS01835,NRI_RS01875,NHE_RS01880	conserved hypothetical protein	Hypothetical proteins | Conserved
NSE_RS01840,NRI_RS01880,NHE_RS01885	Protein of unknown function (DUF3442)	Hypothetical proteins | Conserved
NSE_RS01860,NRI_RS01905,NHE_RS01905	conserved hypothetical protein	Hypothetical proteins | Conserved
NSE_RS01985,NRI_RS02025,NHE_RS02030	conserved hypothetical protein TIGR00103	Hypothetical proteins | Conserved
NSE_RS02015,NRI_RS02060,NHE_RS02070	hypothetical protein	Hypothetical proteins | Conserved
NSE_RS02050,NRI_RS02095,NHE_RS02105	conserved hypothetical protein	Hypothetical proteins | Conserved
NSE_RS02080,NRI_RS02125,NHE_RS02135, NHE_RS02140	hypothetical protein	Hypothetical proteins | Conserved
NSE_RS02115,NRI_RS02160,NHE_RS02185	conserved hypothetical protein	Hypothetical proteins | Conserved
NSE_RS02140,NRI_RS02185,NHE_RS02215	conserved domain protein	Hypothetical proteins | Conserved
NSE_RS02150,NRI_RS02195,NHE_RS02230	hypothetical protein	Hypothetical proteins | Conserved
NSE_RS02160,NRI_RS02205,NHE_RS02240	hypothetical protein	Hypothetical proteins | Conserved
NSE_RS02210,NRI_RS02255,NHE_RS02290	hypothetical protein	Hypothetical proteins | Conserved
NSE_RS02315,NRI_RS02365,NHE_RS02405	hypothetical protein	Hypothetical proteins | Conserved
NSE_RS02320,NRI_RS02370,NHE_RS02410	hypothetical protein	Hypothetical proteins | Conserved
NSE_RS02345,NRI_RS02395,NHE_RS02435	hypothetical protein	Hypothetical proteins | Conserved
NSE_RS02385,NRI_RS02435,NHE_RS02480	conserved hypothetical protein	Hypothetical proteins | Conserved
NSE_RS02420,NRI_RS02475,NHE_RS02525	hypothetical protein	Hypothetical proteins | Conserved
NSE_RS02435,NRI_RS02490,NHE_RS02540	hypothetical protein	Hypothetical proteins | Conserved
NSE_RS02490,NRI_RS02550,NHE_RS02595	hypothetical protein	Hypothetical proteins | Conserved
NSE_RS02575,NRI_RS02645,NHE_RS02695	conserved domain protein	Hypothetical proteins | Conserved
NSE_RS02590,NRI_RS02660,NHE_RS02710	conserved hypothetical protein	Hypothetical proteins | Conserved
NSE_RS02600,NRI_RS02670,NHE_RS02720	conserved hypothetical protein TIGR01033	Hypothetical proteins | Conserved
NSE_RS02615,NRI_RS02685,NHE_RS02735	hypothetical protein	Hypothetical proteins | Conserved
NSE_RS02630,NRI_RS02700,NHE_RS02750	hypothetical protein	Hypothetical proteins | Conserved
NSE_RS02665,NRI_RS02755,NHE_RS02775	hypothetical protein	Hypothetical proteins | Conserved

NSE_RS02680,NRI_RS02770,NHE_RS02790	OmpH-like outer membrane protein	Hypothetical proteins | Conserved
NSE_RS02710,NRI_RS02800,NHE_RS02830	hypothetical protein	Hypothetical proteins | Conserved
NSE_RS02845,NRI_RS02935,NHE_RS02965	conserved hypothetical protein TIGR00150	Hypothetical proteins | Conserved
NSE_RS02855,NRI_RS02945,NHE_RS02975	hypothetical protein	Hypothetical proteins | Conserved
NSE_RS02875,NRI_RS02965,NHE_RS03000	hypothetical protein	Hypothetical proteins | Conserved
NSE_RS02880,NRI_RS02970,NHE_RS03005	conserved hypothetical protein	Hypothetical proteins | Conserved
NSE_RS02955,NRI_RS04080,NHE_RS03080	hypothetical protein	Hypothetical proteins | Conserved
NSE_RS02960,NRI_RS03050,NHE_RS03085	hypothetical protein	Hypothetical proteins | Conserved
NSE_RS02970,NRI_RS03060,NHE_RS03100	hypothetical protein	Hypothetical proteins | Conserved
NSE_RS03035,NRI_RS03125,NHE_RS03190	hypothetical protein	Hypothetical proteins | Conserved
NSE_RS03040,NRI_RS03130,NHE_RS03195	hypothetical protein	Hypothetical proteins | Conserved
NSE_RS03115,NRI_RS03200,NHE_RS03265	hypothetical protein	Hypothetical proteins | Conserved
NSE_RS03185,NRI_RS03270,NHE_RS03345	hypothetical protein	Hypothetical proteins | Conserved
NSE_RS03190,NRI_RS03275,NHE_RS03350	hypothetical protein	Hypothetical proteins | Conserved
NSE_RS03195,NRI_RS03280,NHE_RS03355	hypothetical protein	Hypothetical proteins | Conserved
NSE_RS03205,NRI_RS03290,NHE_RS03365	hypothetical protein	Hypothetical proteins | Conserved
NSE_RS03235,NRI_RS03315,NHE_RS03395	hypothetical protein	Hypothetical proteins | Conserved
NSE_RS03180,NSE_RS03390,NRI_RS03465, NHE_RS03565,NRI_RS03265,NHE_RS03340	tRNA-i(6)A37 modification enzyme MiaB	Hypothetical proteins | Conserved
NSE_RS03540,NRI_RS03620,NHE_RS03705	hypothetical protein	Hypothetical proteins | Conserved
NSE_RS03580,NRI_RS03660,NHE_RS03745	hypothetical protein	Hypothetical proteins | Conserved
NSE_RS03590,NRI_RS03670,NHE_RS03755	maf protein	Hypothetical proteins | Conserved
NSE_RS03625,NRI_RS03710,NHE_RS03790	conserved hypothetical protein	Hypothetical proteins | Conserved
NSE_RS03635,NRI_RS03720,NHE_RS03800	conserved hypothetical protein	Hypothetical proteins | Conserved
NSE_RS03675,NRI_RS03760,NHE_RS03840	conserved hypothetical protein	Hypothetical proteins | Conserved
NSE_RS03685,NRI_RS03770,NHE_RS03850	hypothetical protein	Hypothetical proteins | Conserved
NSE_RS03740,NHE_RS03910,NRI_RS03820, NRI_RS03825	hypothetical protein	Hypothetical proteins | Conserved
NSE_RS03815,NRI_RS03895,NHE_RS04010	conserved hypothetical protein	Hypothetical proteins | Conserved
NSE_RS03820,NRI_RS03900,NHE_RS04015	conserved hypothetical protein	Hypothetical proteins | Conserved
NSE_RS03925,NRI_RS04005,NHE_RS04130	hypothetical protein	Hypothetical proteins | Conserved
NSE_RS03940,NRI_RS04020,NHE_RS04155	conserved domain protein	Hypothetical proteins | Conserved
NSE_RS03965,NRI_RS04045,NHE_RS00020	conserved hypothetical protein	Hypothetical proteins | Conserved
