# Supplementary material for: Analysis of complete genome sequence and major surface antigens of Neorickettsia helminthoeca, causative agent of salmon poisoning disease
Source: Microb Biotechnol. 2017 Jun 6;10(4):933–57. doi: 10.1111/1751-7915.12731 (PMC5481527; doi:10.1111/1751-7915.12731)
Supplement: Supplementary file 7 — Table S2. N. helminthoeca‐specific proteins compared to N. sennetsu and N. risticii [file MBT2-10-933-s007.pdf]

Supplementary Table 2. *N. helminthoeca*-specific proteins compared to *N. sennetsu* and *N. ristici*i [1]

Locus_ID	Protein Name	AA#	Main Role	Sub Role	Top Hits Species (Class, E-value) [2]
NHE_RS00250	aspartate kinase domain protein	397	Amino acid biosynthesis	Aspartate family	*Bacillus muralis* (Bacilli, $3e^{-55}$)
NHE_RS02825	succinyl-diaminopimelate desuccinylase	371	Amino acid biosynthesis	Aspartate family	*Wolbachia* sp. of *Drosophila simulans* (α-Proteobacteria, $1e^{-94}$)
NHE_RS03445	dihydrodipicolinate reductase, family protein	151	Amino acid biosynthesis	Aspartate family	*Campylobacter ureolyticus* (ε-Proteobacteria, $2e^{-20}$)
NHE_RS03415	magnesium chelatase, subunit ChlI family protein	1049	Biosynthesis of cofactors and prosthetic groups	Chlorophyll and bacteriochlorphyll	
NHE_RS00710	UDP-N-acetylglucosamine diphosphorylase (glucosamine-1-phosphate N-acetyltransferase)	432	Cell envelope	Biosynthesis and degradation of murein sacculus and peptidoglycan	*Paracoccus tibetensis* (α-Proteobacteria, $4e^{-122}$)
NHE_RS03095	phosphoglucosamine mutase	439	Cell envelope	Biosynthesis and degradation of murein sacculus and peptidoglycan	*Magnetospirillum marisnigri* (α-Proteobacteria, $2e^{-143}$)
NHE_RS01455	penicillin binding transpeptidase domain protein	528	Cell envelope	Biosynthesis and degradation of murein sacculus and peptidoglycan	*Ca.* Neoehrlichia lotoris (α-Proteobacteria, $2e^{-126}$)
NHE_RS03220	rod shape-determining MreC family protein	256	Cell envelope	Biosynthesis and degradation of murein sacculus and peptidoglycan	
NHE_RS02450	D-alanyl-D-alanine carboxypeptidase family protein	399	Cell envelope	Biosynthesis and degradation of murein sacculus and peptidoglycan	*Wolbachia* sp. of *Cimex lectularius* (α-Proteobacteria, e^{-114})
NHE_RS02495	D-ala D-ala ligase family protein	313	Cell envelope	Biosynthesis and degradation of murein sacculus and peptidoglycan	*Wolbachia* sp. of *Cimex lectularius* (α-Proteobacteria, $5e^{-60}$)
NHE_RS03375	UDP-N-acetylmuramate--alanine ligase	424	Cell envelope	Biosynthesis and degradation of murein sacculus and peptidoglycan	*Thermodesulfovibrio* sp. N1 (Nitrospirales, $2e^{-79}$)
NHE_RS04135	UDP-N-acetylglucosamine 1-carboxyvinyltransferase	418	Cell envelope	Biosynthesis and degradation of murein sacculus and peptidoglycan	*Ca.* Pelagibacter sp. IMCC9063 (α-Proteobacteria, $3e^{-109}$)
NHE_RS02795	phospho-N-acetylmuramoyl-pentapeptide-transferase	324	Cell envelope	Biosynthesis and degradation of murein	*Bacillus bataviensis* (Bacilli, $4e^{-68}$)

1 *N. helminthoeca*-specific proteins were identified by comparison with *N. sennetsu* and *N. risticii* protein databases using BLASTP algorithm with E-value < $1e^{-10}$.

2 *N. helminthoeca*-specific proteins were blasted against NCBI protein database NR excluding *Neorickettsia* spp. with E-value < $1e^{-10}$. The species, class, and E-value of the top matches to the *N. helminthoeca* proteins were listed. Blank fields, no matches were identified based on the search criteria.

				sacculus and peptidoglycan	
NHE_RS02980	D-alanyl-D-alanine carboxypeptidase family protein	284	Cell envelope	Biosynthesis and degradation of murein sacculus and peptidoglycan	*Crenothrix polyspora* (γ-Proteobacteria, 8e^{-68})
NHE_RS00945	UDP-N-acetylmuramoylalanine--D-glutamate ligase	468	Cell envelope	Biosynthesis and degradation of murein sacculus and peptidoglycan	*Robiginitomaculum antarcticum* (α-Proteobacteria, 3e^{-57})
NHE_RS00380	undecaprenyldiphospho-muramoylpentapeptide β-N-acetyl-glucosaminyl-transferase	338	Cell envelope	Biosynthesis and degradation of murein sacculus and peptidoglycan	*Caedibacter varicaedens* (α-Proteobacteria, 3e^{-57})
NHE_RS03115	penicillin binding transpeptidase domain protein	566	Cell envelope	Biosynthesis and degradation of murein sacculus and peptidoglycan	*Anaplasma marginale* (α-Proteobacteria, 2e^{-108})
NHE_RS03175	diaminopimelate epimerase DapF	266	Cell envelope	Biosynthesis and degradation of murein sacculus and peptidoglycan	*Prochlorococcus marinus* (Synechococcales, 9e^{-30})
NHE_RS03990	D-alanine aminotransferase	310	Cell envelope	Biosynthesis and degradation of murein sacculus and peptidoglycan	*Ralstonia solanacearum* (β-Proteobacteria, 1e^{-58})
NHE_RS01430	putative membrane protein	164	Cell envelope	Other	
NHE_RS04200	rare lipoA family protein	226	Cell envelope	Other	*Wolbachia pipientis* (α-Proteobacteria, 1e^{-43})
NHE_RS01810	cell division protein FtsW	369	Cellular processes	Cell division	*Caedibacter varicaedens* (α-Proteobacteria, 4e^{-91})
NHE_RS02385	rod shape-determining protein RodA	377	Cellular processes	Cell division	Rhodospirillaceae bacterium BRH_c57 (α-Proteobacteria, 4e^{-77})
NHE_RS02100	hydroxyacylglutathione hydrolase	247	Cellular processes	Detoxification	*Vibrio halioticoli* (γ-Proteobacteria, 2e^{-67})
NHE_RS00090	carbonic anhydrase family protein	213	Central intermediary metabolism	Other	*Desulfovibrio vulgaris* (δ-Proteobacteria, 1e^{-62})
NHE_RS01115	ribosomal protein L29	63	Protein synthesis	Ribosomal proteins: synthesis and modification	
NHE_RS03975	ribosomal L36 family protein	44	Protein synthesis	Ribosomal proteins: synthesis and modification	
NHE_RS03330	putative Mg chelatase-like domain protein	116	Transport and binding proteins	Unknown substrate	
NHE_RS03535	tol-Pal system beta propeller repeat protein TolB	426	Transport and binding proteins	Other	*Thalassospira profundimaris* (α-Proteobacteria, 1e^{-126})
NHE_RS02060	hexapeptide transferase family protein	611	Transport and binding proteins	Unknown substrate	

NHE_RS03955	biopolymer transport ExbD/TolR family protein	136	Transport and binding proteins	Cations and iron carrying compounds	*Xanthobacter* sp. 126 (α-Proteobacteria, $2e^{-29}$)
NHE_RS03960	motA/TolQ/ExbB proton channel family protein	218	Transport and binding proteins	Cations and iron carrying compounds	*Rickettsia prowazekii* (α-Proteobacteria, $1e^{-42}$)
NHE_RS02515	BON domain protein	535	Unknown function	General	
NHE_RS01320	quinolinate synthetase A family protein	236	Unknown function	Enzymes of unknown specificity	
NHE_RS04220	bacterial SH3 domain protein	148	Unknown function	General	*Wolbachia* sp. of *Cimex lectularius* (α-Proteobacteria, $5e^{-20}$)
NHE_RS01565	bacterial transferase hexapeptide family protein	280	Unknown function	Enzymes of unknown specificity	*Ehrlichia chaffeensis* (α-Proteobacteria, $7e^{-113}$)
NHE_RS02225	inhibitor of apoptosis-promoting Bax1 family protein	321	Unclassified		
NHE_RS00600	ccmB family protein	203	Unclassified		
NHE_RS00220	conserved hypothetical protein	841	Hypothetical proteins	Conserved	*Klebsormidium flaccidum* (Streptophyta, 8^{e-35})
NHE_RS00330	hypothetical protein	123	Unknown function	General	
NHE_RS01050	hypothetical protein	259	Hypothetical proteins	General	
NHE_RS03510	hypothetical protein	407	Hypothetical proteins	General	
NHE_RS00300	hypothetical protein	67	Hypothetical proteins	General	
NHE_RS00555	hypothetical protein	224	Hypothetical proteins	General	
NHE_RS03875	hypothetical protein	84	Hypothetical proteins	General	
NHE_RS00420	hypothetical protein	257	Hypothetical proteins	General	
NHE_RS02660	hypothetical protein	401	Hypothetical proteins	General	
NHE_RS02195	hypothetical protein	59	Hypothetical proteins	General	
NHE_RS00350	hypothetical protein	208	Hypothetical proteins	General	
NHE_RS01395	hypothetical protein	60	Hypothetical proteins	General	
NHE_RS03520	hypothetical protein	403	Hypothetical proteins	General	
NHE_RS00320	hypothetical protein	272	Hypothetical proteins	General	
NHE_RS03885	hypothetical protein	70	Hypothetical proteins	General	
NHE_RS02765	hypothetical protein	133	Hypothetical proteins	General	
NHE_RS00425	hypothetical protein	286	Hypothetical proteins	General	
NHE_RS00665	hypothetical protein	292	Hypothetical proteins	General	
NHE_RS02365	hypothetical protein	434	Hypothetical proteins	General	

NHE_RS00355	hypothetical protein	118	Hypothetical proteins	General	
NHE_RS01500	hypothetical protein	287	Hypothetical proteins	General	
NHE_RS03525	hypothetical protein	601	Hypothetical proteins	General	
NHE_RS00325	hypothetical protein	201	Hypothetical proteins	General	
NHE_RS03925	hypothetical protein	99	Hypothetical proteins	General	
NHE_RS03130	hypothetical protein	60	Hypothetical proteins	General	
NHE_RS00430	hypothetical protein	91	Hypothetical proteins	General	
NHE_RS00725	hypothetical protein	138	Hypothetical proteins	General	
NHE_RS00360	hypothetical protein	98	Hypothetical proteins	General	
NHE_RS02570	hypothetical protein	711	Hypothetical proteins	General	
NHE_RS03950	hypothetical protein	285	Hypothetical proteins	General	*Ca.* Neoehrlichia lotoris (α-Proteobacteria, $1e^{-31}$)
NHE_RS01550	hypothetical protein	93	Hypothetical proteins	General	
NHE_RS03860	hypothetical protein	126	Hypothetical proteins	General	
NHE_RS00335	hypothetical protein	92	Hypothetical proteins	General	
NHE_RS00435	hypothetical protein	111	Hypothetical proteins	General	
NHE_RS00755	hypothetical protein	74	Hypothetical proteins	General	
NHE_RS00365	hypothetical protein	260	Hypothetical proteins	General	
NHE_RS02575	hypothetical protein	561	Hypothetical proteins	General	
NHE_RS04070	hypothetical protein	441	Hypothetical proteins	General	
NHE_RS01935	hypothetical protein	182	Hypothetical proteins	General	
NHE_RS03865	hypothetical protein	70	Hypothetical proteins	General	
NHE_RS00340	hypothetical protein	247	Hypothetical proteins	General	
NHE_RS00865	hypothetical protein	141	Hypothetical proteins	General	
NHE_RS03180	hypothetical protein	103	Hypothetical proteins	General	
NHE_RS00540	hypothetical protein	373	Hypothetical proteins	General	
NHE_RS00415	hypothetical protein	277	Hypothetical proteins	General	
NHE_RS02580	hypothetical protein	699	Hypothetical proteins	General	
NHE_RS04100	hypothetical protein	101	Hypothetical proteins	General	
NHE_RS02145	hypothetical protein	93	Hypothetical proteins	General	
NHE_RS00345	hypothetical protein	201	Hypothetical proteins	General	

NHE_RS00440	hypothetical protein	163	Unknown function	General
NHE_RS00445	hypothetical protein	152	Unknown function	General
