# Supplementary material for: Analysis of complete genome sequence and major surface antigens of Neorickettsia helminthoeca, causative agent of salmon poisoning disease
Source: Microb Biotechnol. 2017 Jun 6;10(4):933–57. doi: 10.1111/1751-7915.12731 (PMC5481527; doi:10.1111/1751-7915.12731)
Supplement: Supplementary file 8 — Table S3. N. risticii‐specific proteins compared to N. helminthoeca and N. sennetsu [file MBT2-10-933-s008.pdf]

Supplementary Table 3. ***N. ristici*i-specific proteins compared to *N. helminthoeca* and *N. sennetsu*** [1]

Locus_ID	Protein Name	Protein Length	Main Role	Sub Role
NRI_RS00085	hypothetical protein	83	Unknown function	General
NRI_RS00095	hypothetical protein	76	Unknown function	General
NRI_RS00240	hypothetical protein	60	Unknown function	General
NRI_RS00315	hypothetical protein	219	Unknown function	General
NRI_RS00325	hypothetical protein	62	Unknown function	General
NRI_RS00365	hypothetical protein	210	Unknown function	General
NRI_RS00370	hypothetical protein	208	Unknown function	General
NRI_RS00415	hypothetical protein	74	Unknown function	General
NRI_RS00440	hypothetical protein	111	Unknown function	General
NRI_RS00460	hypothetical protein	81	Unknown function	General
NRI_RS00485	hypothetical protein	82	Unknown function	General
NRI_RS00615	hypothetical protein	205	Unknown function	General
NRI_RS00770	hypothetical protein	76	Unknown function	General
NRI_RS01090	hypothetical protein	91	Unknown function	General
NRI_RS01340	hypothetical protein	63	Unknown function	General
NRI_RS01900	hypothetical protein	60	Unknown function	General
NRI_RS02350	hypothetical protein	104	Unknown function	General
NRI_RS02630	hypothetical protein	61	Unknown function	General
NRI_RS02740	hypothetical protein	118	Unknown function	General
NRI_RS03370	hypothetical protein	65	Unknown function	General
NRI_RS03385	hypothetical protein	60	Unknown function	General
NRI_RS03470	hypothetical protein	59	Unknown function	General
NRI_RS00320	conserved hypothetical protein	352	Hypothetical proteins	Conserved
NRI_RS00380	conserved hypothetical protein	179	Hypothetical proteins	Conserved
NRI_RS02090	conserved hypothetical protein	77	Hypothetical proteins	Conserved
NRI_RS02530	conserved hypothetical protein	310	Hypothetical proteins	Conserved
NRI_RS02730	conserved hypothetical protein	278	Hypothetical proteins	Conserved
NRI_RS03680	conserved hypothetical protein	71	Hypothetical proteins	Conserved

[1] *N. risticii*-specific proteins were identified by comparison with *N. helminthoeca* and *N. sennetsu* protein databases using BLASTP algorithm with E-value < $1e^{-10}$.
