# Supplementary material for: Analysis of complete genome sequence and major surface antigens of Neorickettsia helminthoeca, causative agent of salmon poisoning disease
Source: Microb Biotechnol. 2017 Jun 6;10(4):933–57. doi: 10.1111/1751-7915.12731 (PMC5481527; doi:10.1111/1751-7915.12731)
Supplement: Supplementary file 9 — Table S4. N. sennetsu‐specific proteins compared to N. helminthoeca and N. risticii [file MBT2-10-933-s009.pdf]

Supplementary Table 4. *N. sennetsu*-specific proteins compared to *N. helminthoeca* and *N. risticii* [1]

Locus_ID	Protein Name	Protein Length	Main Role	Sub Role
NSE_RS00095	hypothetical protein	85	Unknown function	General
NSE_RS00100	hypothetical protein	92	Unknown function	General
NSE_RS00120	hypothetical protein	69	Unknown function	General
NSE_RS00325	hypothetical protein	72	Unknown function	General
NSE_RS00330	hypothetical protein	118	Unknown function	General
NSE_RS00370	hypothetical protein	180	Unknown function	General
NSE_RS00425	hypothetical protein	79	Unknown function	General
NSE_RS00545	hypothetical protein	59	Unknown function	General
NSE_RS00565	hypothetical protein	335	Unknown function	General
NSE_RS00575	hypothetical protein	72	Unknown function	General
NSE_RS00720	hypothetical protein	70	Unknown function	General
NSE_RS01695	hypothetical protein	59	Unknown function	General
NSE_RS01705	hypothetical protein	62	Unknown function	General
NSE_RS01715	hypothetical protein	132	Unknown function	General
NSE_RS01960	hypothetical protein	59	Unknown function	General
NSE_RS02045	hypothetical protein	83	Unknown function	General
NSE_RS03065	hypothetical protein	64	Unknown function	General
NSE_RS03225	hypothetical protein	62	Unknown function	General
NSE_RS03270	hypothetical protein	69	Unknown function	General
NSE_RS03305	hypothetical protein	77	Unknown function	General
NSE_RS03385	hypothetical protein	68	Unknown function	General
NSE_RS03710	hypothetical protein	61	Unknown function	General
NSE_RS03795	hypothetical protein	100	Unknown function	General

[1] *N. sennetsu*-specific proteins were identified by comparison with *N. helminthoeca* and *N. risticii* protein databases using BLASTP algorithm with E-value < $1e^{-10}$.
