# Supplementary material for: Analysis of complete genome sequence and major surface antigens of Neorickettsia helminthoeca, causative agent of salmon poisoning disease
Source: Microb Biotechnol. 2017 Jun 6;10(4):933–57. doi: 10.1111/1751-7915.12731 (PMC5481527; doi:10.1111/1751-7915.12731)
Supplement: Supplementary file 10 — Table S5. Amino acid and cofactor biosynthesis in Family Anaplasmataceae [file MBT2-10-933-s010.pdf]

Supplementary Table 5. Amino acid and cofactor biosynthesis in Family Anaplasmataceae

Organisms [1]	NHO	NRI	NES	APH	ECH
**Amino Acids:** [2]					
**Alanine**	+	+	+	+	+
Arginine	-	-	-	+ [3]	**+**
Asparagine	-	-	-	+	+
**Aspartate**	+	+	+	+	+
Cysteine	-	-	-	-	-
**Glycine**	+	+	+	+	+
**Glutamate** [4]	+	+	+	+	+
**Glutamine**	+	+	+	+	+
Histidine	-	-	-	-	-
Leucine	-	-	-	-	-
Lysine	- [5]	-	-	-	**+**
Isoleucine	-	-	-	-	-
Methionine	-	-	-	-	-
Phenylalanine	-	-	-	-	-
Proline	-	-	-	-	-
Serine	-	-	-	-	-
Threonine	-	-	-	-	-
Tryptophan	-	-	-	-	-
Tyrosine	-	-	-	-	-
Valine	-	-	-	-	-
**Cofactors:**					
Biotin	+	+	+	+	+
FAD	+	+	+	+	+
Folate	+	+	+	+	+
Lipoate	+	+	+	+	+
NAD	+	+	+	+	+
CoA [6]	+	+	+	+	+
Protoheme	+	+	+	+	+
Pyridoxine phosphate (Vitamin B6)	+	+	+	+	+
Thiamine	+	+	+	+	+
Ubiquinone	+	+	+	+	+

[1] Abbreviations: ECH, *Ehrlichia chaffeensis* Arkansas; APH, *Anaplasma phagocytophilum* HZ; NSE, *N. sennetsu* Miyayama; NRI, *N. risticii* Illinois; NHO, *N. helminthoeca* Oregon.

[2] *N. helminthoeca* encodes genes for 5 amino acids biosynthesis, which are converted from other AAs or metabolic intermediates.

[3] Only partial enzymes are identified in Arginine biosynthesis pathway in APH.

[4] *Ech* and *APH* can convert Pro to Glu through PutA (bifunctional proline dehydrogenase/pyrroline-5-carboxylate dehydrogenase). All Anaplasmataceae can convert Gln to Glu by CarA/B (carbamoyl phosphate synthase) or GS/PH (bifunctional glutamate synthase subunit beta/2-polyprenylphenol hydroxylase).

[5] *N. helminthoeca* encodes complete pathways to synthesize meso-2,6-diaminopimelate (*m*DAP) from L-Asp, but lacks diaminopimelate decarboxylase (LysA) at the last step to produce lysine.

[6] ECH and APH can synthesize CoA from pantothenate, however, all *Neorickettsia* spp. can only convert 4'-phosphopantetheine to CoA.
