# Supplementary material for: Analysis of complete genome sequence and major surface antigens of Neorickettsia helminthoeca, causative agent of salmon poisoning disease
Source: Microb Biotechnol. 2017 Jun 6;10(4):933–57. doi: 10.1111/1751-7915.12731 (PMC5481527; doi:10.1111/1751-7915.12731)
Supplement: Supplementary file 11 — Table S6. Potential pathogenic genes in Neorickettsia species [file MBT2-10-933-s011.pdf]

Supplementary Table 6. Potential pathogenic genes in *Neorickettsia* species

Organisms [1]	NHO	NRI	NSE
**Type I Secretion System (T1SS):**			
ATP-binding cassette (ABC) transporter HlyB	+	+	+
Membrane fusion protein (MFP) HlyD	+	+	+
Outer membrane channel protein TolC	+	+	+
**TAT Pathway:**			
twin-arginine translocation protein, TatA/E family	+	+	+
twin-arginine translocation protein, TatB	+	+	+
Sec-independent protein translocase TatC	+	+	+
**Type IV Secretion System (T4SS):**			
VirB1	-	-	-
VirB2	+ (3)	+ (2)	+ (2)
VirB3	+	+	+
VirB4	+ (2)	+ (2)	+ (2)
VirB5	-	-	-
VirB6	+ (4)	+ (4)	+ (4)
VirB7	+	+	+
VirB8	+ (2)	+ (2)	+ (2)
VirB9	+ (2)	+ (2)	+ (2)
VirB10	+	+	+
VirB11	+	+	+
VirD4	+	+	+
**Two-component Systems:**			
PleC/PleD [2]	+	+	+
CckA/CtrA	+	+	+
NtrY/NtrX	-	-	-
**Putative Secreted Effectors:**			
Ankyrin-repeat domain proteins	4	4	3

[1] Numbers inside parentheses indicate the copy numbers of the genes; otherwise, only a single copy is present. Abbreviations: NHO, *N. helminthoeca* Oregon; NRI, *N. risticii* Illinois; NSE, *N. sennetsu* Miyayama.

[2] All *Neorickettsia* spp. encodes two copies of sensor histidine kinase PleC.
