# Supplementary material for: Analysis of complete genome sequence and major surface antigens of Neorickettsia helminthoeca, causative agent of salmon poisoning disease
Source: Microb Biotechnol. 2017 Jun 6;10(4):933–57. doi: 10.1111/1751-7915.12731 (PMC5481527; doi:10.1111/1751-7915.12731)
Supplement: Supplementary file 12 — Table S7. Putative Transporters of N. helminthoeca [file MBT2-10-933-s012.pdf]

Supplementary Table 7. Putative Transporters of *N. helminthoeca*

Locus ID	Protein Name	Gene	Gene Family	Transporter Family / Subfamily	Substrate/Function
NHE_RS00575	sodium:alanine symporter family protein		AGCS	The Alanine or Glycine:Cation Symporter (AGCS) Family	sodium ion:alanine symporter
NHE_RS00175	ABC-type protease/lipase transport system		ABC	The ATP-binding Cassette (ABC) Superfamily / ABC+ membrane	protease secretion
NHE_RS01715	ABC-type multidrug transport system MdlB		ABC	The ATP-binding Cassette (ABC) Superfamily / ABC+ membrane	multidrug
NHE_RS02960	heme ABC exporter ATP-binding protein CcmA	CcmA	ABC	The ATP-binding Cassette (ABC) Superfamily / binding	heme
NHE_RS00600	ccmB family protein	CcmB	ABC	The ATP-binding Cassette (ABC) Superfamily / membrane	heme export
NHE_RS02125	heme exporter protein, CcmC family	CcmC	ABC	The ATP-binding Cassette (ABC) Superfamily / membrane	heme export
NHE_RS00045	iron-binding protein FbpA	FbpA	ABC	The ATP-binding Cassette (ABC) Superfamily / Binding	iron(III)
NHE_RS01265	putative transporter	FbpB	ABC	The ATP-binding Cassette (ABC) Superfamily / membrane	iron(III)
NHE_RS01995	ABC-type Fe3+/spermidine/putrescine transport systems	FbpC	ABC	The ATP-binding Cassette (ABC) Superfamily / binding	Polyamine or iron(III)
NHE_RS01315	ABC-type lipoprotein export system		ABC	The ATP-binding Cassette (ABC) Superfamily / binding	lipoprotein
NHE_RS01370	CBS domain protein, putative		ABC	The ATP-binding Cassette (ABC) Superfamily / binding	glycine betaine
NHE_RS02220	inosine-5'-monophosphate dehydrogenase		ABC	The ATP-binding Cassette (ABC) Superfamily / binding	glycine betaine
NHE_RS02955	ABC transporter ATP-binding protein		ABC	The ATP-binding Cassette (ABC) Superfamily / binding	phosphate
NHE_RS01990	phosphate ABC transporter, permease protein PstA	PstA	ABC	The ATP-binding Cassette (ABC) Superfamily / membrane	phosphate
NHE_RS03450	phosphate ABC transporter ATP-binding protein	PstB	ABC	The ATP-binding Cassette (ABC) Superfamily / binding	phosphate
NHE_RS00795	phosphate ABC transporter, permease protein PstC	PstC	ABC	The ATP-binding Cassette (ABC) Superfamily / membrane	phosphate
NHE_RS03695	ABC transporter (iron-sulfur clusters)		ABC	The ATP-binding Cassette (ABC) Superfamily / binding	lipid A
NHE_RS04010	conserved hypothetical protein		ABC	The ATP-binding Cassette (ABC) Superfamily / binding	toluene tolerance
NHE_RS02235	mce-related protein		ABC	The ATP-binding Cassette (ABC) Superfamily / binding protein	toluene tolerance
NHE_RS04005	putative VacJ lipoprotein		ABC	The ATP-binding Cassette (ABC) Superfamily / binding protein	?
NHE_RS00530	lipoprotein releasing system transmembrane protein LolE		ABC	The ATP-binding Cassette (ABC) Superfamily / membrane	lipoprotein releasing
NHE_RS02950	ABC transporter permease protein		ABC	The ATP-binding Cassette (ABC) Superfamily / membrane	toluene tolerance
NHE_RS00740	permease, PerM family		AI-2E	The Autoinducer-2 Exporter (AI-2E) Family (Formerly the PerM Family, TC #9.B.22)	Autoinducer-2 export
NHE_RS00660	auxin Efflux Carrier		AEC	The Auxin Efflux Carrier (AEC) Family	
NHE_RS01325	ComEC/Rec2 family protein		DNA-T	The Bacterial Competence-related DNA Transformation Transporter (DNA-T) Family	
NHE_RS00335	hypothetical protein		CaCA	The Ca2+:Cation Antiporter (CaCA) Family	proton:calcium ion antiporter

NHE_RS00345	hypothetical protein		CDF	The Cation Diffusion Facilitator (CDF) Family	cation efflux
NHE_RS01685	inner membrane protein, 60 kDa		Oxa1	The Cytochrome Oxidase Biogenesis (Oxa1) Family	60 KD inner membrane protein OxaA homolog
NHE_RS00770	putative transporter		DAACS	The Dicarboxylate/Amino Acid:Cation (Na+ or H+) Symporter (DAACS) Family	proton/sodium ion:glutamate/aspartate symporter
NHE_RS00590	putative transporter		DASS	The Divalent Anion:Na+ Symporter (DASS) Family	sodium ion:dicarboxylate/sulfate
NHE_RS00295	integral membrane protein DUF6		DMT	The Drug/Metabolite Transporter (DMT) Superfamily	drug/metabolite?
NHE_RS03960	motA/TolQ/ExbB proton channel family protein		Mot/ Exb	The H+- or Na+-translocating Bacterial Flagellar Motor 1ExbBD Outer Membrane Transport Energizer (Mot/Exb)	
NHE_RS00505	ATP synthase F1, alpha subunit		F-ATPase	The H+- or Na+-translocating F-type, V-type and A-type ATPase (F-ATPase) Superfamily	protons
NHE_RS00510	ATP synthase F1, delta subunit		F-ATPase	The H+- or Na+-translocating F-type, V-type and A-type ATPase (F-ATPase) Superfamily	protons
NHE_RS01655	ATP synthase F0, A subunit		F-ATPase	The H+- or Na+-translocating F-type, V-type and A-type ATPase (F-ATPase) Superfamily	protons
NHE_RS01660	conserved domain protein		F-ATPase	The H+- or Na+-translocating F-type, V-type and A-type ATPase (F-ATPase) Superfamily	protons
NHE_RS01665	ATP synthase F0, B' chain		F-ATPase	The H+- or Na+-translocating F-type, V-type and A-type ATPase (F-ATPase) Superfamily	protons
NHE_RS01670	putative ATPase F0, B chain		F-ATPase	The H+- or Na+-translocating F-type, V-type and A-type ATPase (F-ATPase) Superfamily	protons
NHE_RS02510	ATP synthase F1, gamma subunit		F-ATPase	The H+- or Na+-translocating F-type, V-type and A-type ATPase (F-ATPase) Superfamily	protons
NHE_RS03260	ATP synthase F1, alpha subunit		F-ATPase	The H+- or Na+-translocating F-type, V-type and A-type ATPase (F-ATPase) Superfamily	protons
NHE_RS01690	CBS domain protein, putative		HCC	The HlyC/CorC (HCC) Family	heavy metal ion
NHE_RS00290	drug resistance transporter, Bcr/CflA family		MFS	The Major Facilitator Superfamily (MFS)	multidrug efflux
NHE_RS03325	major facilitator family transporter		MFS	The Major Facilitator Superfamily (MFS)	multidrug efflux
NHE_RS03475	putative permease		MFS	The Major Facilitator Superfamily (MFS)	Acetyl-CoA:CoA antiporter
NHE_RS03605	major facilitator family transporter		MFS	The Major Facilitator Superfamily (MFS)	glycerol-3-phosphate
NHE_RS01920	magnesium transporter		MgtE	The Mg2+ Transporter-E (MgtE) Family	magnesium ion
NHE_RS03965	membrane protein, putative		MC	The Mitochondrial Carrier (MC) Family	
NHE_RS03970	membrane protein, putative		MC	The Mitochondrial Carrier (MC) Family	
NHE_RS00075	NADH-quinone oxidoreductase chain l	MnhA	CPA3	The Monovalent Cation (K+ or Na+):Proton Antiporter-3 (CPA3) Family	multicomponent sodium ion:proton antiporter
NHE_RS02400	NADH-quinone oxidoreductase chain l	MnhA	CPA3	The Monovalent Cation (K+ or Na+):Proton Antiporter-3 (CPA3) Family	multicomponent sodium ion:proton antiporter

NHE_RS00135	Domain of unknown function (DUF4040)	MnhB	CPA3	The Monovalent Cation (K+ or Na+):Proton Antiporter-3 (CPA3) Family	multicomponent sodium ion:proton antiporter
NHE_RS00140	multisubunit Na+/H+ antiporter, MnhB subunit	MnhB	CPA3	The Monovalent Cation (K+ or Na+):Proton Antiporter-3 (CPA3) Family	multicomponent sodium ion:proton antiporter
NHE_RS00130	monovalent cation/proton antiporter, MnhC/PhaC subunit family	MnhC	CPA3	The Monovalent Cation (K+ or Na+):Proton Antiporter-3 (CPA3) Family	multicomponent sodium ion:proton antiporter
NHE_RS02990	NADH-quinone oxidoreductase chain l	MnhD	CPA3	The Monovalent Cation (K+ or Na+):Proton Antiporter-3 (CPA3) Family	multicomponent sodium ion:proton antiporter
NHE_RS02185	conserved hypothetical protein	MnhE	CPA3	The Monovalent Cation (K+ or Na+):Proton Antiporter-3 (CPA3) Family	multicomponent sodium ion:proton antiporter
NHE_RS00145	monovalent cation/proton antiporter, MnhG/PhaG subunit	MnhG	CPA3	The Monovalent Cation (K+ or Na+):Proton Antiporter-3 (CPA3) Family	multicomponent sodium ion:proton antiporter
NHE_RS00705	multiple resistance and pH regulation protein F (MrpF / PhaF)		CPA3	The Monovalent Cation (K+ or Na+):Proton Antiporter-3 (CPA3) Family	sodium ion:proton antiporter
NHE_RS03770	glutathione-regulated potassium-efflux system protein		CPA2	The Monovalent Cation:Proton Antiporter-2 (CPA2) Family	potassium/sodium ion:proton antiporter
NHE_RS02395	membrane protein, MviN family	MviN	MOP	The Multidrug/Oligosaccharidyl-lipid/Polysaccharide (MOP) Flippase Superfamily / MVF	virulence factor MviN
NHE_RS03705	conserved hypothetical protein		OAT	The Organo Anion Transporter (OAT) Family	organic anion
NHE_RS00745	transporter, AcrB/AcrD/AcrF family	HAE1	RND	The Resistance-Nodulation-Cell Division (RND) Superfamily	multidrug/solvent efflux (HAE1 subfamily)
NHE_RS03610	mechanosensitive ion channel family protein		MscS	The Small Conductance Mechanosensitive Ion Channel (MscS) Family	small-conductance mechanosensitive ion channel
NHE_RS03065	putative sodium:proline symporter		SSS	The Solute:Sodium Symporter (SSS) Family	sodium ion:proline symporter
NHE_RS03385	membrane protein, TerC family		TerC	The Tellurium Ion Resistance (TerC) Family	tellurium ion efflux
NHE_RS03590	trap transporter, 4tm/12tm fusion protein		TRAP-T	The Tripartite ATP-independent Periplasmic Transporter (TRAP-T) Family	C4-dicarboxylate
NHE_RS02000	Twin-arginine translocation protein, TatA/E family	TatA	Tat	The Twin Arginine Targeting (Tat) Family	protein export
NHE_RS00490	twin arginine-targeting protein translocase TatC	TatC	Tat	The Twin Arginine Targeting (Tat) Family	protein export
NHE_RS03165	type IV secretion system protein VirB8 (VirB8-1)	VirB8-1	IVSP	The Type IV (Conjugal DNA-Protein Transfer or VirB) Secretory Pathway (IVSP) Family	
NHE_RS03145	type IV secretion system protein VirD4	VirD4	IVSP	The Type IV (Conjugal DNA-Protein Transfer or VirB) Secretory Pathway (IVSP) Family	
NHE_RS03335	signal peptidase I	LepB	IVSP	Protein and peptide secretion and trafficking family	
NHE_RS03675	type IV secretion system protein, VirB6 family (VirB6-2)	VirB6-2	YggT	The YggT or Fanciful K+ Uptake-B (FkuB; YggT) Family	potassium ion uptake?
NHE_RS03670	type IV secretion system protein, VirB6 family (VirB6-3)	VirB6-3	YggT	The YggT or Fanciful K+ Uptake-B (FkuB; YggT) Family	potassium ion uptake?
NHE_RS03665	type IV secretion system protein, VirB6 family (VirB6-4)	VirB6-4	YggT	The YggT or Fanciful K+ Uptake-B (FkuB; YggT) Family	potassium ion uptake?
