# Supplementary material for: Analysis of complete genome sequence and major surface antigens of Neorickettsia helminthoeca, causative agent of salmon poisoning disease
Source: Microb Biotechnol. 2017 Jun 6;10(4):933–57. doi: 10.1111/1751-7915.12731 (PMC5481527; doi:10.1111/1751-7915.12731)
Supplement: Supplementary file 13 — Table S8. Genes involved in DNA repair and homologous recombination [file MBT2-10-933-s013.pdf]

Supplementary Table 8. Genes involved in DNA repair and homologous recombination [1]

	ECH	APH	NSE	NRI	NHO
**Direct Repair**					
Photolyase			NSE_RS03995 / NSE_RS04000 *	NRI_RS03480	
DNA ligase ligA	ECH0301	APH0138	NSE_RS02025	NRI_RS02070	NHE_RS02080
**AP Endonuclease**					
Xth	ECH0675	APH0505	NSE_RS01685	NRI_RS01735	NHE_RS01735
**Base Excision Repair Glycosylases (BER)**					
3mg	ECH0277				
Ung Family 4	ECH0074	APH1371	NSE_RS03805	NRI_RS03885	NHE_RS04000
Fpg	ECH0602	APH0411			
Nth	ECH0857	APH0897	NSE_RS00975	NRI_RS01015	NHE_RS00975
**Nucleotide Excision Repair (NER)**					
UvrA	ECH0785	APH0537			
UvrB		APH1367			
UvrC		APH0884			
UvrD	ECH0860	APH0903	NSE_RS01465		NHE_RS01505
UvrD family	ECH0387	APH0258	NSE_RS01885	NRI_RS01930	NHE_RS01930
**Transcription Coupling Repair (TCR)**					
Mfd	ECH0250	APH0107			
**Mismatch Repair (MMR)**					
MutL	ECH0884	APH0939	NSE_RS02475	NRI_RS02535	
MutS	ECH0824	APH0857	NSE_RS01390	NRI_RS01440	NHE_RS04185
**Homologous Recombination**					
<u>RecF Pathway</u>					
RecF	ECH0076	APH1409	NSE_RS00780	NRI_RS00820	NHE_RS00775
RecJ	ECH1115	APH1165	NSE_RS02895	NRI_RS02985	NHE_RS03020
RecO	ECH0536	APH0736	NSE_RS01855	NRI_RS01895	NHE_RS01900
RecR	ECH0843	APH0988	NSE_RS03670	NRI_RS03755	NHE_RS03835

[1] Abbreviations: ECH, *Ehrlichia chaffeensis* Arkansas; APH, *Anaplasma phagocytophilum* HZ; NSE, *N. sennetsu* Miyayama; NRI, *N. risticii* Illinois; NHO, *N. helminthoeca* Oregon.

* Proteins are truncations due to an internal mutation.

Recombinase					
RecA	ECH1109	APH1354	NSE_RS02170	NRI_RS02215	NHE_RS02250
Holliday junction resolution					
RuvA	ECH0320	APH0167	NSE_RS02360	NRI_RS02410	NHE_RS02455
RuvB	ECH0319	APH0166	NSE_RS02365	NRI_RS02415	NHE_RS02460
RuvC	ECH0028	APH0018	NSE_RS03885	NRI_RS03965	NHE_RS04085
RecG	ECH0062	APH1298	NSE_RS02795	NRI_RS02885	NHE_RS02915
Other recombination					
RadA	ECH0305				
**Other**					
RadC	ECH0363	APH0242	NSE_RS00915	NRI_RS04065/ NRI_RS04060*	NHE_RS00910*
XseL	ECH0056	APH1322			
XseS	ECH0214	APH0079			
Hu		APH0784	NSE_RS02595	NRI_RS02665	NHE_RS02715
RmuC	ECH0577	APH0428	NSE_RS00485	NRI_RS00530	NHE_RS00480
