# Supplementary material for: Analysis of complete genome sequence and major surface antigens of Neorickettsia helminthoeca, causative agent of salmon poisoning disease
Source: Microb Biotechnol. 2017 Jun 6;10(4):933–57. doi: 10.1111/1751-7915.12731 (PMC5481527; doi:10.1111/1751-7915.12731)
Supplement: Supplementary file 14 — Table S9. Proteins with tandem repeats in N. helminthoeca [file MBT2-10-933-s014.pdf]

Supplementary Table 9. Proteins with tandem repeats in *N. helminthoeca* [1]

Locus ID	Protein Name	Location of Repeats	Repeat Length	Number of Repeats
**NHE_RS00170**	conserved hypothetical protein	284 - 346	30	2
Repeats:	AGPRGEDARANVGDPNLPRSSSLPNPNVSHGQE			
**NHE_RS00220**	hypothetical protein	449 - 788	20	17
Repeats:	TRSHGDLTEMRKALSREPSP			
**NHE_RS00965**	51 kDa antigen (**P51**)	39 - 56	6	3
Repeats:	CGCKKT			
**NHE_RS04180**	conserved hypothetical protein	219 - 468	25	10
Repeats:	VEVQTDAPEEPERSTGAASTQTMSE			
**NHE_RS01860**	conserved hypothetical protein			
Repeats 1:	PIPSAEVAQQPAAEPVQQATE	147 – 209	21	3
Repeats 2:	VEQGSDDNTGADNIEEAIEPIPPAEVAQQPAAEPVQQATEPIPS	18 – 107	46	2
**NHE_RS02060**	hexapeptide transferase family protein	109 - 284	35	5
Repeats:	GEISTGPEAITEATEVQDEVKLNPEVITEASGIVD			
**NHE_RS02225**	inhibitor of apoptosis-promoting Bax1 family protein	94 – 159	33	2
Repeats:	DRVSDAMPGIQKGAKSTVVWTADAAGRVGAVML			
**NHE_RS02305**	RDD family protein	18 – 31	7	2
Repeats:	FPHKVFS			
**NHE_RS02365**	hypothetical protein	201 – 224	8	3
Repeats:	EIMNTTNK			
**NHE_RS02540**	conserved hypothetical protein	346 - 526	36	5
Repeats:	SSTGSCRPIAAPILNGASLHGVYTSLFEGNKDPGTV			
**NHE_RS02570**	hypothetical protein	478 – 702	75	3
Repeats:	LRKVGIKEKPFTGDDLIAELKARIEKRSEKNPGKPTVSDSRKRMVTSDAKDSKQRETQGEKSGN PRTITTETTLE			
**NHE_RS02695**	conserved hypothetical protein	329 – 402	37	2
Repeats:	VPATSAVMKSIASTGEGGEVVGLSPTLTKFLKEVGEV			
**NHE_RS03510**	conserved hypothetical protein	123 – 272	30	5
Repeats:	AKYYSAHRDEILQRRRESRARDPERFCEYG			
**NHE_RS03520**	hypothetical protein	52 – 175	30	4
Repeats 1:	PEKFREYKAKHYSAHRDEILQRRRESRARD			
Repeats 2:	KYYSAHRDEILQRRRESRARDPEKFRGYGA	229 - 288	30	2

[1] Tandem repeats in protein were analyzed by T-REKS (http://bioinfo.montp.cnrs.fr/?r=t-reks) (Jorda and Kajava, 2009).

**NHE_RS03525**	hypothetical protein	15 – 231	74	3
Repeats 1:	KKKAEQPIQGTSSSSAPGPSTADLSTSSGSTTVLAPKRRKLTPEEKRERNRISQAKYYSAHRDE IIQRQREQRA			
Repeats 2:	ERFREYKAKHYSAHRDEILQRRRESRARDP	313 – 402	30	3
**NHE_RS03670**	type IV secretion system protein, VirB6-3	1007 - 1243	47	5
Repeats:	KPKTGEGMVENPIYESGDPVQGAESTENPYSLRGAEGQEEPIYATVD			
**NHE_RS03855**	Neorickettsia strain-specific surface antigen (**SSA**)	43 - 137	23	5
Repeats:	AAEVLKNTTAGDILKNST			
**NHE_RS04070**	hypothetical protein	130 – 353	56	4
Repeats:	NAPPESLQIELTLDQSEDSSEKQPITPPQQTEPVSLQHQIEPTAPPEPHKTEPVIV			
