# Supplementary material for: Analysis of complete genome sequence and major surface antigens of Neorickettsia helminthoeca, causative agent of salmon poisoning disease
Source: Microb Biotechnol. 2017 Jun 6;10(4):933–57. doi: 10.1111/1751-7915.12731 (PMC5481527; doi:10.1111/1751-7915.12731)
Supplement: Supplementary file 15 — Table S10. Lipoprotein‐processing enzymes and putative lipoproteins in N. helminthoeca [file MBT2-10-933-s015.pdf]

Supplementary Table 10. Lipoprotein processing enzymes and putative lipoproteins in *N. helminthoeca*

Locus ID	Protein Name	Protein Length	LipoBox Sequences [1]
**Lipoprotein processing enzymes:**			
NHE_RS03645	prolipoprotein diacylglyceryl transferase (Lgt)	264	n/a
NHE_RS03900	signal peptidase II (LspA)	167	n/a
NHE_RS02065	apolipoprotein N-acyltransferase (Lnt)	472	n/a
**Predicted Lipoproteins:**			
NHE_RS00570	efflux transporter, RND family, MFP subunit	342	IFLCS|CLKDS
NHE_RS00745	acriflavine resistance protein AcrB	1023	FGSYA|CFVIP
NHE_RS01690	CBS domain protein	421	SLLLS|CVFSG
NHE_RS01870	beta-ketoacyl-[acyl-carrier-protein] synthase II	416	LGLVT|CLSSK
NHE_RS02525	conserved hypothetical protein	323	FSLSS|CAKRG
NHE_RS02980	D-alanyl-D-alanine carboxypeptidase	284	SSLAH|CTSAI
NHE_RS03040	outer membrane protein assembly complex YaeT	744	LFLDP|CLAEN
NHE_RS03070	pentapeptide repeat domain protein	552	CSSAD|CSHTS
NHE_RS03100	conserved hypothetical protein	304	LCFAP|CHSLE
NHE_RS03665	type IV secretion system protein VirB6-4	1069	FTFSG|CDHCE
NHE_RS03670	type IV secretion system protein VirB6-3	1243	FLFNG|CDIEC
NHE_RS03785	peptidoglycan-associated lipoprotein (PAL/OmpA)	200	LLMSG|CFKKG
NHE_RS03940	BamD lipoprotein	235	LVVSG|CTPGK

[1] Putative lipoprotein was predicted by LipoP 1.0 (http://www.cbs.dtu.dk/services/LipoP). "|" indicate the predicted signal peptidase II cleavage site.
