# Supplementary material for: Analysis of complete genome sequence and major surface antigens of Neorickettsia helminthoeca, causative agent of salmon poisoning disease
Source: Microb Biotechnol. 2017 Jun 6;10(4):933–57. doi: 10.1111/1751-7915.12731 (PMC5481527; doi:10.1111/1751-7915.12731)
Supplement: Supplementary file 16 — Table S11. Oligonucleotide primers used for cloning N. helminthoeca outer membrane proteins [file MBT2-10-933-s016.pdf]

Supplementary Table 11. Oligonucleotide primers used for cloning *N. helminthoeca* outer membrane proteins

Target genes	Primer Sequence (5' → 3') [1]	Amplicon size
p51 (NHE_RS00965)	F: ATAGGCCATGG CTTCTGTAGAGAACCCATCAA<br>R: CTAGAGAATTC GTATATGATACTTTGAGACCTGAAG	1,422 bp
nsp1 (NHE_RS03715)	F: ATAGGCCATGG CGCTTTTCGGAATAAACGC<br>R: CTAGAGAATTC AATATTCCAAGCTGGATCTTGATTCC	703 bp
nsp2 (NHE_RS03720)	F: ATAGGCCATGG CCAAAGTAGAAGAAGCGGCGAATGC<br>R: CTAGAGCGGCCGC GGCGTCAAGTGAAAAAGTAAC	870 bp
nsp3 (NHE_RS03725)	F: ATAGGCCATGG CGCAAGATGCCCTAGAGGATG<br>R: CTAGAGCGGCCGC ATTCATAGGTAGCATTAG	624 bp
ssa (NHE_RS03855)	F: ATAGGCCATGG ATCTGCTTAAGCATGATACCTCAAG<br>R: CTAGAGCGGCCGC TTTTTTGGGGATAGTTATCTCTTTAAGTC	1,002 bp

[1] Underlined sequences indicate restriction enzymes' recognition sites: F, Forward (*Nco*I); R, Reverse complement (*EcoR*I for *p51* and *nsp*1, *Not*I for *nsp*2/3 and *ssa*). Stop codons and approximate 80 bp from 5'-end of these genes that encodes signal peptides were excluded in the amplicon.
