# Supplementary material for: Analysis of complete genome sequence and major surface antigens of Neorickettsia helminthoeca, causative agent of salmon poisoning disease
Source: Microb Biotechnol. 2017 Jun 6;10(4):933–57. doi: 10.1111/1751-7915.12731 (PMC5481527; doi:10.1111/1751-7915.12731)
Supplement: Supplementary file 17 [file MBT2-10-933-s017.docx]

# Supplementary Figure Legends

**Fig. S1. Synteny plots between *Neorickettsia* spp.** The entire genomes of *N. helminthoeca* and *N. risticii* or *N. sennetsu* were aligned using MUMmer3 with default parameters. Each axis represents the genomic coordinates for the respective organisms with red points reflecting matches on the forward strand and blue points reflecting matches on the reverse strand.

**Fig. S2. Secondary Structure of *N. helminthoeca* P51 Protein.** The two-dimensional structure of the *N. helminthoeca* P51 protein were predicted using PRED-TMBB analysis and image drawn by TMRPres2D (<http://biophysics.biol.uoa.gr/PRED-TMBB/>). The discrimination value for *N. helminthoeca* P51 is 2.949, which is below the threshold value of 2.965, suggesting that it is a β-barrel protein localized to the outer membrane with 18 transmembrane domains.

**Fig. S3. Phylogenetic tree of VirB2 proteins in the family Anaplasmataceae and α-proteobacteria.** Protein sequences of VirB2 from members of the family Anaplasmataceae and representative α-proteobacteria were aligned using the ClustalW method, and a phylogenetic tree was built using the MegAlign program of the Lasergene DNAstar package. Nho VirB2s, analyzed in this study from *N. helminthoeca* based on sequence homology to other *Neorickettsia* VirB2; Nse, *N. sennetsu* Miyayama; Nri, *N. risticii* Illinois; APH, *A. phagocytophilum* HZ; ECH, *E. chaffeensis* Arkansas; ATU6168, *Agrobacterium tumefaciens* C58 pilin subunit VirB2 (Accession No. NP_396488); RP192, *Rickettsia prowazekii* Madrid E VirB2 (Accession No. NP_359878); RC241, *Rickettsia conorii* Malish 7 VirB2 (Accession No. NP_359878); CC2417, *Caulobacter crescentus* CB15 VirB2 (Accession No. NP_421220).

**Fig. S4. One-component regulatory systems of *N. helminthoeca***. The presence of genes encoding one-component regulatory systems in *N. helminthoeca* was predicted based on Microbial Signal Transduction Database (http://mistdb.com/). Domain architecture of each protein is predicted using the Pfam database. * Not identified in *E. chaffeensis* and *A. phagocytophilum.*

**Domain abbreviations and functions:** HTH, DNA-binding helix-turn helix domain; MerR, MerR family regulatory domain (DNA-binding, winged helix-turn-helix domain of about 70 residues present in the merR family of transcriptional regulators); Rrf2, Transcriptional regulator; Aminotran_5, Aminotransferase class V; EAL, EAL domain (diguanylate phosphodiesterase activity for degradation of a second messenger, cyclic di-GMP. Together with the GGDEF domain, EAL might be involved in regulating cell surface adhesiveness in bacteria); HD, HD domain (metal-dependent phosphohydrolases).

**Fig. S5. Phylogenetic analysis of AnkA or Ank200 homologous proteins in the family Anaplasmataceae.** Homologies of *A. phagocytophilum* HZ AnkA (GenBank accession No. WP_011450840) or *E. chaffeensis* Arkansas Ank200 (GenBank # WP_011452759) from representative members of the family Anaplasmataceae were first determined by Blast searches using *E. chaffeensis* Arkansas Ank200. Protein sequences were aligned using the ClustalW method, and a phylogenetic tree was built using the MegAlign program of the Lasergene DNAstar package. GenBank accession numbers for AnkA/Ank200 homologies are: *N. helminthoeca* Oregon, WP_038558671; *N. sennetsu* Miyayama, WP_011451432; *N. risticii* Illinois, WP_012779418; *A. marginale* St Maries, WP_011114402; *E. canis* Jake, WP_011304486; *E. ruminantium* Gardel, WP_011255523; *Wolbachia pipientis* wMel, WP_010962493.
